# Hybrid Thoracoscopic‐Endocardial Versus Endocardial Catheter Ablation for Persistent Atrial Fibrillation: A Systematic Review and Meta‐Analysis

**DOI:** 10.1002/joa3.70416

**Published:** 2026-07-10

**Authors:** Annelyse Vitória Barbosa, Bárbara Reis Silva, Pedro Lucas Alves Alencar, Silvia Marçal Botelho, Aguinaldo Figueredo Freitas Junior, Antônio da Silva Menezes Júnior

**Affiliations:** ^1^ Internal Medicine Department, Medical Sciences and Life School Pontifical Catholic University of Goiás Goiás Brazil; ^2^ Faculty of Medicine, Internal Medicine Department Federal University of Goiás Goiás Brazil

**Keywords:** anti‐arrhythmia agents, atrial fibrillation, catheter ablation, hospitalization, length of stay, meta‐analysis, randomized controlled trials

## Abstract

**Background:**

Hybrid ablation (HA) addresses the limitations of endocardial catheter ablation (CA) in patients with persistent, long‐standing atrial fibrillation (AF). This review compares hybrid thoracoscopic‐endocardial ablation with endocardial CA in this population to evaluate rhythm‐control outcomes and procedural safety.

**Methods:**

Randomized controlled trials and observational studies comparing HA with CA in adults with non‐paroxysmal AF were identified through searches of PubMed, Embase, and Cochrane databases (November 2025). The review was registered with PROSPERO (CRD42024600526). Primary outcomes included freedom from AF, any atrial arrhythmia, use of antiarrhythmic drugs (AADs), arrhythmia recurrence, and repeat ablation.

**Results:**

Eight studies (907 patients: 478 HA; 429 CA) were included. Compared to CA, HA did not significantly reduce AF (OR 2.38; 95% CI 0.66–8.65) or arrhythmia recurrence (OR 1.66; 95% CI 0.31–9.06). HA significantly reduced any atrial arrhythmia (OR 3.36; 95% CI 2.16–5.23) and AADs (OR 3.25; 95% CI 2.20–4.82) and repeat ablation rates (OR 0.28; 95% CI 0.09–0.90). HA increased risk of significant complications (OR 2.73; 95% CI 1.18–6.27) despite a significantly shorter fluoroscopy time. Heterogeneity reflected variations in lesion sets, epicardial energy modality, surgical access, timing, and comparator CA strategies. Recent randomized trials show comparable safety profiles for HA and CA when hybrid procedures are performed in experienced multidisciplinary programs.

**Conclusion:**

HA offers better rhythm control than CA in persistent AF, although it involves greater procedural complexity. In appropriately selected patients and institutions with established hybrid expertise, HA is an effective rhythm‐control strategy that yields clinically significant benefits.

## Graphical abstract

Hybrid thoracoscopic‐endocardial ablation improved overall rhythm control, antiarrhythmic drug independence, and reduced repeat ablation compared with endocardial catheter ablation in persistent atrial fibrillation. These benefits were offset by greater procedural complexity and a higher risk of major complications.

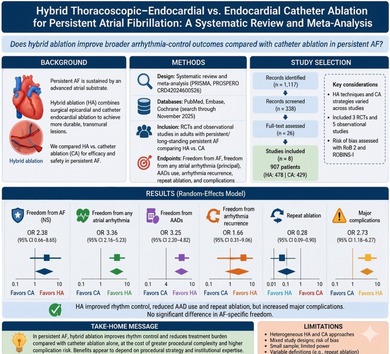

## 1 Introduction

Atrial fibrillation (AF), the most prevalent persistent arrhythmia globally, presents a significant public health challenge due to its association with stroke, heart failure, and elevated mortality rates [1]. Catheter ablation (CA), which involves pulmonary vein isolation, is a recognized rhythm‐management strategy for paroxysmal AF. However, CA is less effective in patients with persistent AF [2]. As AF progresses to more advanced stages, the arrhythmia is increasingly sustained by a modified atrial substrate rather than by triggers from the pulmonary veins alone. Progressive atrial fibrosis, slowed conduction, and the development of re‐entrant circuits diminish the longevity of endocardial lesions and reduce the likelihood of maintaining a sustained sinus rhythm [3, 4]. Despite the use of adjunctive techniques, such as linear lesions or complex fractionated atrial electrogram ablation, the success rates for a single surgery remain low, necessitating multiple procedures. Hybrid ablation (HA) has emerged as a novel technique to address these challenges. HA produces more uniform, transmural lesion sets by integrating surgical epicardial ablation with endocardial catheter imaging and lesion completion, including durable posterior wall isolation and alteration of the left atrial substrate [4, 5]. Numerous prospective randomized trials, including CONVERGE, CEASE‐AF, and HARTCAP‐AF, as well as observational studies, have demonstrated that rhythm control is significantly enhanced with HA compared to CA alone. This suggests that a combined epicardial‐endocardial approach may offer significant clinical benefits in patients with non‐paroxysmal AF.

Despite the encouraging results, the evidence base remains inconclusive and limited. Studies conducted thus far have employed diverse hybrid techniques, including approaches to the epicardium, various energy sources (unipolar vs. bipolar radiofrequency), different lesion configurations (e.g., convergent posterior wall ablation vs. thoracoscopic box lesion), and varying staging strategies (simultaneous vs. sequential) [6]. This heterogeneity is further compounded by differences in patient characteristics, criteria for assessing therapeutic effectiveness, and methods of rhythm monitoring. Consequently, the actual extent, reliability, and safety of HA compared to CA remain uncertain.

The notable gaps in the evidence are as follows: although meta‐analysis demonstrated improved rhythm outcomes with HA, these findings were limited by methodological flaws, small sample sizes, and outdated research. Van der Heijden et al. indicated that, compared to CA, HA (70.7%) was more effective in stabilizing sinus rhythm (49.9%). However, the research assessed the groups separately rather than directly comparing the two treatments [7]. Eranki et al. reported an odds ratio of 2.78 in favor of HA for maintaining independence from AF; however, the synthesis included only four randomized controlled trials and corresponding cohort studies, involving just 422 patients, and was based on data available only until early 2022 [8]. Therefore, these prior analyses exclude some of the most robust and recent studies and do not adequately address procedure‐level variability.

The increasing utilization of HA necessitates the collection and analysis of novel randomized data. Given the ongoing ambiguity surrounding comparative safety and effectiveness, an updated and methodologically sound systematic review and meta‐analysis is necessary. Consequently, we aimed to consolidate the most recent randomized and observational data comparing hybrid thoracoscopic–endocardial ablation with endocardial CA in patients with persistent and long‐standing persistent AF and to evaluate both rhythm‐control outcomes and procedural safety.

## 2 Methods

The protocol for this systematic review and meta‐analysis was registered with the International Prospective Register of Systematic Reviews (PROSPERO) under the registration number CRD42024600526. This study was designed and reported in accordance with the Preferred Reporting Items for Systematic Reviews and Meta‐Analysis (PRISMA) Reporting Guidelines [9].

### 2.1 Study Eligibility

This meta‐analysis included only studies that met all the following eligibility criteria: (1) randomized trials or observational studies; (2) adult patients (age ≥ 18 years) with persistent AF or longstanding persistent AF; and (3) patients who underwent HA or conventional endocardial CA. Studies were included only if they reported at least one of the clinical outcomes of interest.

We excluded studies that (1) lacked a control group; (2) involved pediatric populations (aged < 18 years); and (3) did not involve HA or did not compare HA with conventional endocardial CA. There were no restrictions on the publication date, and only English‐language publications were included.

### 2.2 Search Strategy and Data Extraction

The PubMed, Cochrane, and Embase databases were systematically searched in November 2025 using the following search terms: “atrial fibrillation,” “hybrid ablation,” and “catheter ablation.” Data were extracted on the following outcomes: (1) freedom from AF; (2) freedom from arrhythmia (regardless of antiarrhythmic drugs [AADs]); (3) freedom from AADs; (4) arrhythmia recurrence; and (5) repeat ablation.

Given the multifaceted nature of rhythm control in atrial fibrillation, multiple complementary efficacy endpoints were included. Among these, freedom from atrial arrhythmia was considered the principal measure of treatment effectiveness, as it captures the overall burden of atrial tachyarrhythmias, including atrial fibrillation, atrial flutter, and atrial tachycardia. The remaining endpoints, including freedom from atrial fibrillation, antiarrhythmic drug use, arrhythmia recurrence, and repeat ablation, were analyzed as supportive outcomes to provide a comprehensive assessment of clinical benefit.

The definition of repeat ablation was based on the individual studies' reporting. However, it is important to note that some studies may have included planned staged endocardial “touch‐up” procedures as part of the hybrid strategy, whereas others reported only unplanned reinterventions due to arrhythmia recurrence. This distinction could not be consistently standardized across studies and represents a potential source of misclassification.

The occurrence of significant complications, including cardioversion, cardiac tamponade, phrenic nerve paralysis, stroke, and death, was evaluated for the safety assessment. To measure procedural efficiency, we evaluated fluoroscopy time, length of hospital stay, and total procedure duration. All identified articles were systematically evaluated based on predetermined inclusion and exclusion criteria. Article selection and data extraction were conducted independently by at least two reviewers (B.S. and A.B.), with any disagreements resolved by consensus.

### 2.3 Quality Assessment

The Cochrane tool for assessing risk of bias in randomized trials (RoB 2) was employed to evaluate the quality of randomized studies [10], while the Cochrane tool for assessing risk of bias in non‐randomized studies (ROBINS‐I) was used to assess the quality of observational studies [11]. The risk‐of‐bias evaluation was conducted independently by two authors (B.S. and A.B.), and any disagreements were resolved through consensus.

### 2.4 Sensitivity Analysis

To address the variability in procedural definitions across the included studies, we performed a comprehensive set of sensitivity and exploratory analyses. Initially, leave‐one‐out sensitivity analyses were conducted for all efficacy, safety, and procedural outcomes to determine whether the overall results were influenced by any single study. Additionally, we constructed Baujat plots to identify the studies that contributed most to overall heterogeneity and to assess their potential impact on the pooled estimates. Publication bias was evaluated through visual inspection of funnel plots for all efficacy and safety outcomes. Given the relatively small number of studies for each endpoint, funnel‐plot asymmetry was used as the primary method to assess small‐study effects. For safety outcomes, including cardiac tamponade, cardioversion, death, major complications, phrenic nerve paralysis, and stroke, we applied the same framework used for efficacy analyses, incorporating influence diagnostics and heterogeneity exploration to ensure the robustness and consistency of all findings.

Procedural outcomes, including fluoroscopy time, length of hospital stay, and total procedure duration, were assessed using analogous sensitivity analyses, such as leave‐one‐out analyses, subgroup analyses, and heterogeneity diagnostics. Additionally, to further explore potential sources of heterogeneity in the efficacy outcomes, we conducted meta‐regression analyses to examine age, sex, left ventricular ejection fraction, and AF duration as moderator variables. The detailed results, plots, and numerical outputs from these analyses are presented in the Supporting Information.

### 2.5 Data Analysis

Treatment effects for the binary endpoints were compared using pooled odds ratios (ORs) with 95% confidence intervals (CIs), while those for the continuous endpoints were compared using mean differences (MDs) with 95% CIs. The Mantel–Haenszel test was employed for all binary endpoints, and inverse‐variance weighting was applied for the continuous endpoints. Heterogeneity was assessed using Cochran's Q test, *I* ^2^ statistics, and Tau‐squared calculated via the restricted maximum‐likelihood estimator. Heterogeneity was reported as low (*I* ^2^ = 0%–25%), moderate (*I* ^2^ = 26%–50%), or high (*I* ^2^ > 50%). A random‐effects model was utilized for all studies. All statistical analyses were conducted using R version 4.5.1 (R Foundation for Statistical Computing, Vienna, Austria).

## 3 Results

### 3.1 Study Selection and Baseline Characteristics

Our systematic search yielded 1117 potential articles, as seen in Figure S1. After removing duplicate records and excluding studies based on title and abstract review, 26 articles remained and were thoroughly assessed against the inclusion and exclusion criteria. Ultimately, eight studies included three randomized and five non‐randomized, encompassing a total of 907 patients, of whom 478 were assigned to HA and 429 to conventional endocardial CA alone, as detailed in Tables 1, 2, S1, and S2.

**TABLE 1:** Baseline characteristics of included studies.

Study	Design	Patients, N	Age, y† HA/EA	Male, n HA/EA	BMI (kg/m^2^)† HA/EA	LA size, cm † HA/EA	LVEF, (%)† HA/EA	Duration of persistent AF† HA/EA	CHA2DS2‐VASc†, HA/EA
Yu 2024 [5]	Prospective cohort	52/52	60 ± 5.19/60.3 ± 8.89	42/42	26.81 ± 1.96/27.95 ± 3.93	51.83 ± 2.96/50.83 ± 4.8	61.5 ± 5.19/60.0 ± 2.96	4.27 ± 3.93/5.00 ± 6.67^c^	1.33 ± 0.74/1.67 ± 1.48
Van der Heijden 2023 [12]	RCT	19/22	64 ± 9/64 ± 8	18/18	28.4 ± 4/29.2 ± 4	114 ± 35/101 ± 18^b^	55 ± 7/54 ± 8	22/33^d^	10/6^a^
CEASE‐AF 2023	RCT	102/52	60.8 ± 8.1/60.6 ± 7.4	77/38	29.7 ± 3.5/29.8 ± 3.1	4.7 ± 0.5/4.7 ± 0.4	58.3 ± 9.0/57.8 ± 8.5	2.94 ± 3.29/3.34 ± 3.52^c^	22/7^a^
Converge Trial 2020 [4]	RCT	102/51	63.7 ± 9.6/65.1 ± 6.7	80/27	32.9 ± 5.9/35.1 ± 7.1	4.4 ± 0.6/4.3 ± 0.6	55.3 ± 7.8/55.7 ± 6.1	4.4 ± 4.8/4.5 ± 4.7^c^	NI
Maclean 2020 [2]	Retrospective cohort	43/43	68.6 ± 7.7/65.5 ± 7.5	32/32	NI	47.4 ± 6.3/47.5 ± 7.4^e^	50% (15)/50% (20)	36/30^d^	NI
Kress 2017 [13]	Retrospective cohort	64/69	60.7 ± 10.2/62.3 ± 8.0	59/51	35.0 ± 6.2/34.6 ± 7.5	4.8 ± 0.6/4.7 ± 1.2	53.6 ± 11.4/53.4 ± 7.5	16.0 ± 14.8/8.0 ± 5.2^d^	1.8 ± 1.2/2.1 ± 1.4
Edgerton 2016 [6]	Prospective cohort	24/35	63.8 ± 9/63.0 ± 10.4	22/29	30.8 ± 5.00/29.2 ± 4.0	5.15 ± 0.28/5.24 ± 0.47	52.6 ± 8.6/49.2 ± 9.3	6.8 ± 3.5/5.6 ± 3.8^c^	1.13 ± 0.95/1.11 ± 0.99
Hwang 2018 [14]	Non‐randomized observational study	72/105	53.57 ± 8.46/52.02 ± 8.63	71/85	25.29 ± 2.57/25.51 ± 3.09	47.93 ± 14.34/41.60 ± 11.90^f^	59.07 ± 6.48/60.19 ± 6.24	NI	1.39 ± 1.00/1.18 ± 0.99

Abbreviations: EA, endocardial ablation; HA, hybrid ablation; LA, Left atrium; LVEF, Left Ventricular Ejection Fraction; NI, Not Informed; RCT, Randomized Controlled Trial.

^†^ Mean or median.

^a^ N with CHA2DS2‐VASc > 3.

^b^ Volume/ml.

^c^ Years.

^d^ Months.

^e^ Diameter/mm.

^f^ LA volume index (mL/m^2^).

**TABLE 2:** Characteristics of settings and design of included studies.

Study	Design	Sample size	Country	Enrollment period
Chunyu 2024	Prospective Cohort Study	104	China	June 2014 to July 2021
Van der Heijden 2023 [12]	Randomized Controlled Trial	41	Netherlands	October 2016 and December 2018
CEASE‐AF 2023	Randomized Controlled Trial	154	UE	NI
Converge Trial 2020 [4]	Randomized Controlled Trial	153	UK and USA	NI
Maclean 2020 [2]	Retrospective Cohort Study	86	UK	From 2013 to 2018
Kress 2016 [13]	Retrospective Cohort Study	133	USA	June 9, 2010, to August 20, 2014, and June 28, 2011, to August 21, 2014.
Edgerton 2016 [6]	Prospective Cohort Study	59	USA	NI
Hwang 2018 [14]	Non‐randomized observational study	177	USA	From Jan 2012 and April 2015

Abbreviation: NI, Not informed.

### 3.2 Pooled Analysis of All Studies

**FIGURE 1:**
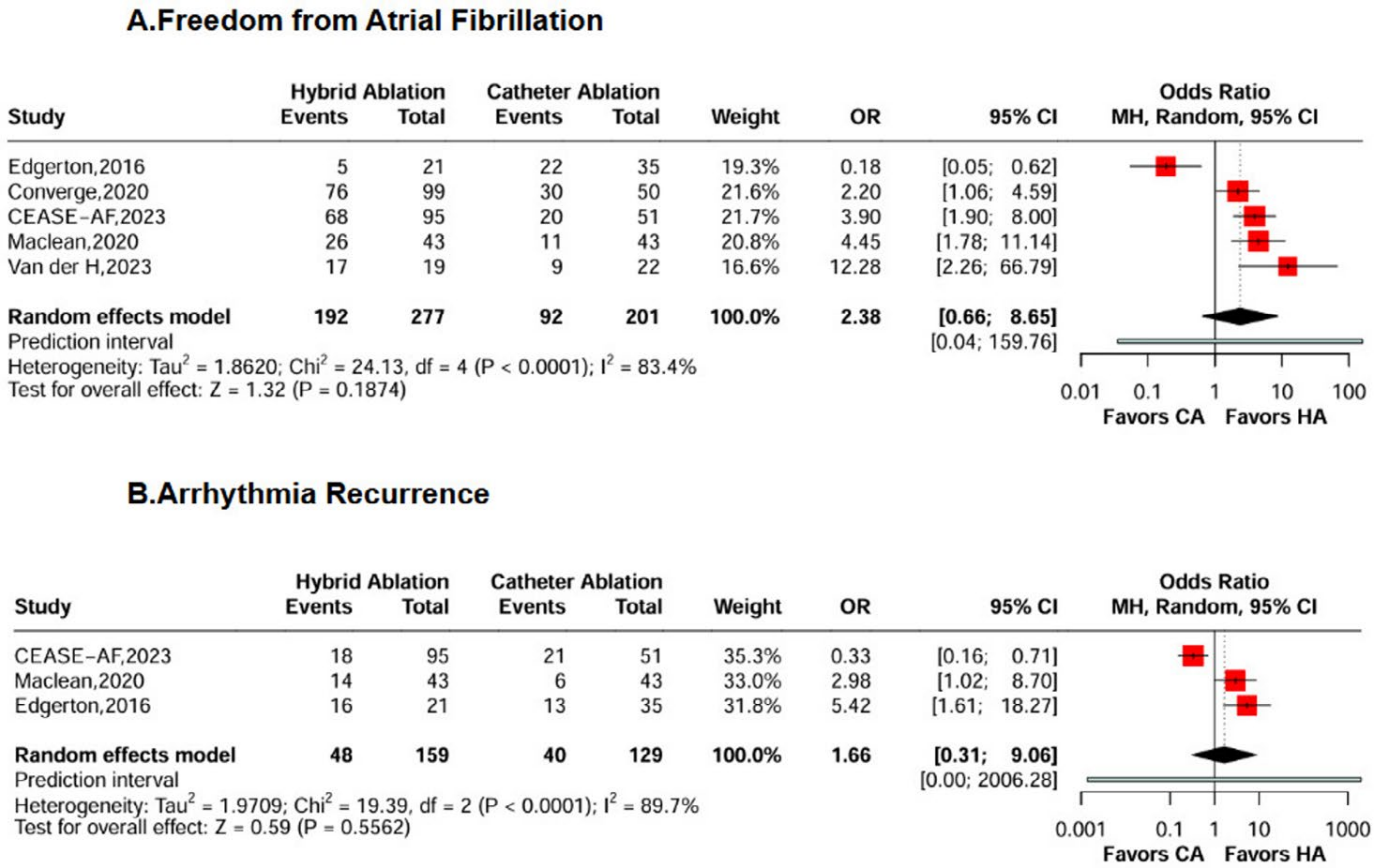
Forest plot of the primary endpoints. (A) Freedom from atrial fibrillation. (B) Arrhythmia recurrence.

**FIGURE 2:**
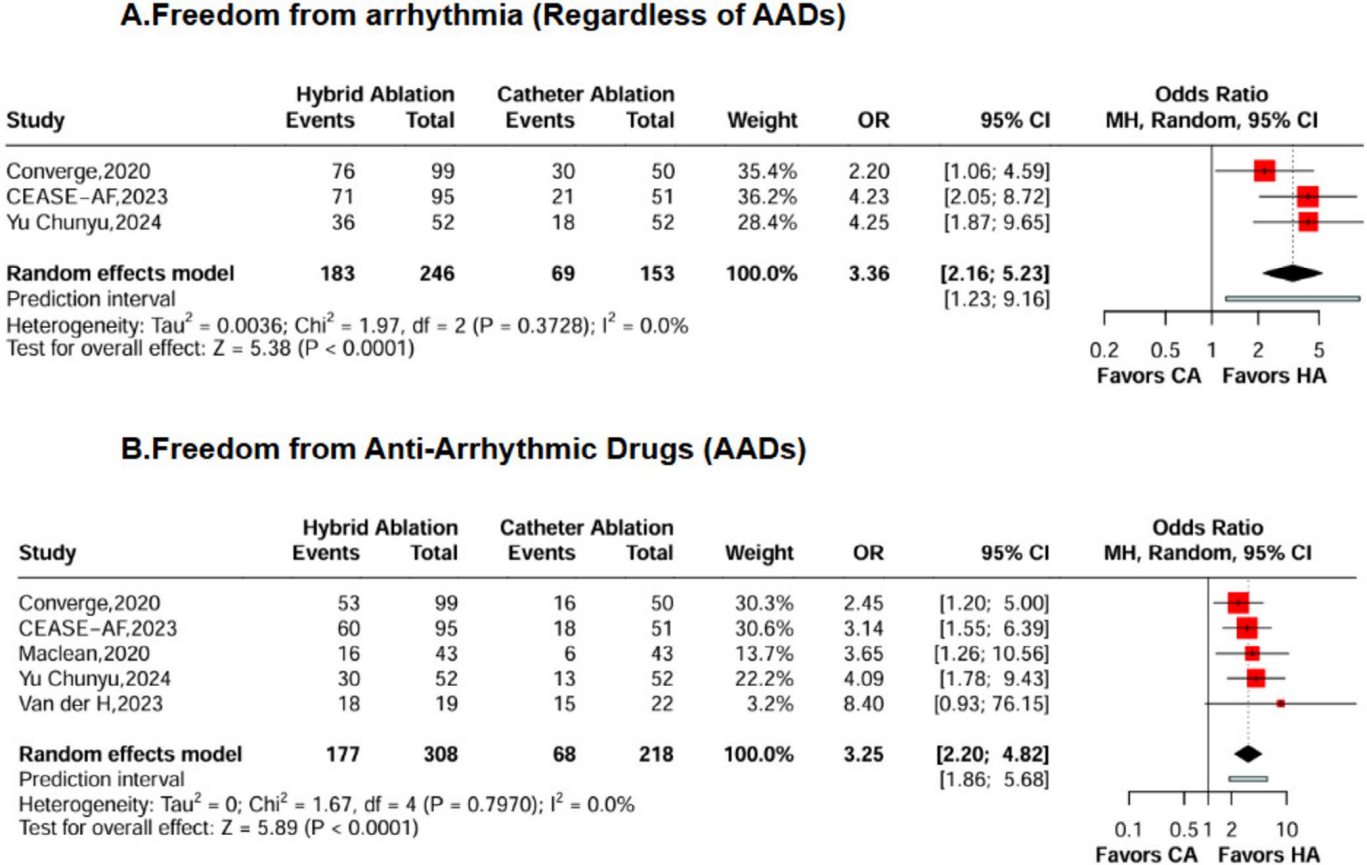
Forest plot of the primary endpoints. (A) Freedom from anti‐arrhythmic drugs (AADs). (B) Repeat ablation.

There was no significant difference between the groups in terms of freedom from AF (OR 2.38; 95% CI 0.66–8.65; *p* = 0.1874) and arrhythmia recurrence (OR 1.66; 95% CI 0.31–9.06; *p* = 0.5562). However, treatment with HA significantly improved freedom from arrhythmia (regardless of AADs; OR 3.36; 95% CI 2.16–5.23; *p* < 0.0001) and freedom from AADs (OR 3.25; 95% CI 2.20–4.82; *p* < 0.0001). Furthermore, HA was associated with a lower rate of repeat ablation compared to CA alone (OR 0.28; 95% CI 0.09–0.90; *p* = 0.0332), as shown in Figures 1 and 2.

### 3.3 Safety Outcomes

No significant differences were observed between groups in terms of cardiac tamponade (OR 0.82; 95% CI 0.22–3.11; *p* = 0.77; *I* ^2^ = 0%), mortality (OR 4.09; 95% CI 0.67–25.11; *p* = 0.13; *I* ^2^ = 0%), phrenic nerve paralysis (OR 3.73; 95% CI 0.38–36.73; *p* = 0.26; *I* ^2^ = 0%), and stroke (OR 0.84; 95% CI 0.22–3.29; *p* = 0.81; *I* ^2^ = 6.6%). The HA group exhibited a significant advantage over the CA group in cardioversion (OR 0.39; 95% CI 0.23–0.65; *p* = 0.0003; *I* ^2^ = 0%), but was associated with a significantly increased risk of serious complications (OR 2.73; 95% CI 1.18–6.27; *p* = 0.018; *I* ^2^ = 0%), as shown in Figure S10A–F.

### 3.4 Procedural Efficiency

In the pooled analysis, compared to CA, HA was associated with a significantly shorter fluoroscopy time (MD −4.72; 95% CI −9.36 to −0.09; *p* = 0.046; *I* ^2^ = 56.9%), but a significantly longer length of hospital stay (MD 2.38; 95% CI 0.52–4.24; *p* = 0.012; *I* ^2^ = 82.1%). Additionally, when compared to CA, HA was consistently associated with a markedly longer total procedure time (MD 80.25; 95% CI 65.32–95.17; *p* < 0.001; *I* ^2^ = 0%), as shown in Figure S14A–D.

### 3.5 Sensitivity Analyses

Sensitivity analyses were conducted using leave‐one‐out exclusion, influence diagnostics, and visual inspection of funnel plots for all efficacy, safety, and procedural outcomes. Throughout the analyses, the direction and magnitude of the pooled estimates remained stable, indicating that no single study influenced the findings.

Sensitivity analyses stratified by study design demonstrated that the direction and magnitude of treatment effects were broadly consistent when analyses were restricted to randomized controlled trials, although statistical precision was reduced due to the limited number of studies. These findings support the overall robustness of the results despite the inclusion of observational data.

### 3.6 Efficacy Outcomes

For freedom from atrial fibrillation, the sequential removal of individual studies did not significantly alter the effect estimate (Figure S1A). Baujat diagnostics showed no disproportionate contribution of any study to the heterogeneity or effect size, and funnel plots showed no significant asymmetry. Similar stability was observed for freedom from AADs, freedom from any arrhythmia, arrhythmia recurrence, and repeat ablation (Figures S1B–E–S3A–E). Across all efficacy outcomes, the consistency of estimates supports the robustness of the observed clinical benefits associated with hybrid ablation.

### 3.7 Safety Outcomes

Leave‐one‐out analyses for safety endpoints—including cardiac tamponade, cardioversion, death, major complications, phrenic nerve paralysis, and stroke—demonstrated that the effect estimates remained unchanged when any individual study was removed (see Figure S11A–F). Baujat plots showed no influential outliers (see Figure S12A–F). Funnel plots were generally symmetrical; however, interpretation was limited due to the rarity of certain events (see Figure 13A–F). Overall, the safety findings appeared consistent and were not influenced by any single dataset.

### 3.8 Procedural Outcomes

For fluoroscopy time, length of stay, and total procedure duration, leave‐one‐out analyses demonstrated minimal variation in pooled estimates (Figure S15A–C). Baujat diagnostics indicated heterogeneity across studies, with no single trial exerting a dominant influence (Figure S17A–C). These findings support the reliability of procedural comparisons between hybrid and endocardial ablation strategies.

### 3.9 Risk of Bias

The risk‐of‐bias assessment utilizing ROBINS‐I for observational studies and RoB 2 for randomized trials showed an overall low‐to‐moderate risk of bias across the included studies (see Figures S18A,B and S19C,D). The primary concerns identified in non‐randomized studies were residual confounding and variability in procedural classification. In contrast, randomized trials demonstrated a low risk of bias in both the randomization process and outcome measurement. No study demonstrated a risk level that would meaningfully alter the treatment effect. These findings, along with the stability of results observed in sensitivity analyses, reinforce the overall robustness of the evidence base.

## 4 Discussion

In this systematic review and meta‐analysis, which included data from 907 patients, we evaluated HA versus conventional endocardial CA for the treatment of persistent AF. HA did not significantly enhance independence from AF, a finding characterized by considerable variability, but it consistently demonstrated superiority in other therapeutically relevant outcomes. Patients undergoing HA achieved higher rates of freedom from any atrial arrhythmia and greater independence from AADs, thereby necessitating fewer repeat ablations than patients receiving CA. These results suggest that HA offers a more sustainable rhythm‐control strategy for non‐paroxysmal AF. Nevertheless, these advantages were associated with an increased incidence of severe complications, prolonged procedural times, and extended hospital stays, despite reductions in fluoroscopy duration and the need for cardioversion.

The observed discrepancy between freedom from atrial fibrillation and freedom from atrial arrhythmia warrants careful interpretation. While atrial fibrillation represents the primary clinical target of ablation, the broader endpoint of atrial arrhythmia includes atrial flutter and atrial tachycardia, which are also clinically relevant and often arise from residual or modified atrial substrate. The greater effect of hybrid ablation on this composite endpoint may reflect its ability to achieve more extensive substrate modification rather than complete elimination of atrial fibrillation alone. From a clinical perspective, a reduction in overall arrhythmia burden and decreased reliance on antiarrhythmic drugs may still translate into meaningful patient benefit, even in the absence of complete freedom from atrial fibrillation.

The therapeutic rationale for HA is based on the structural and electrophysiological remodeling associated with persistent AF. As AF progresses, the arrhythmogenic substrate extends beyond the triggers located in the pulmonary veins. This substrate is characterized by atrial fibrosis, slowed conduction, and a morphology that predisposes individuals to re‐entry phenomena [1, 2, 3, 4, 15, 16]. In this context, CA has inherent limitations due to the challenges in achieving continuous, transmural lesions with endocardial energy delivery, resulting in conduction gaps that may lead to arrhythmia recurrence. HA seeks to address these issues by integrating both epicardial and endocardial approaches. Epicardial ablation allows for direct visualization and the application of bipolar radiofrequency clamps, which can create consistent transmural lesions, thereby facilitating reliable isolation of the posterior wall and treatment of the left atrial appendage [4, 5]. Next, the endocardial step is completed, and the lesion set is assessed by employing high‐resolution mapping to address any remaining gaps. HARTCAP‐AF demonstrated the effectiveness of these components working together, with 42% of patients requiring endocardial touch‐ups following epicardial ablation, thereby affirming the hybrid technique's efficiency [12].

Despite the theoretical benefits of HA, a careful comparison of the results is necessary, given the heterogeneity of the included studies, primarily due to the broad range of hybrid lesion sets. Although hybrid ablation encompasses a spectrum of techniques—including thoracoscopic, convergent, and biatrial approaches—these strategies share a common mechanistic framework that integrates epicardial and endocardial ablation to achieve more durable lesion sets and comprehensive substrate modification. This conceptual similarity supports their inclusion within a single analytical category. However, we acknowledge that these techniques differ substantially in terms of lesion sets, access routes, energy sources, and procedural timing. Such variability may influence both efficacy and safety outcomes and represents an important source of heterogeneity to consider when interpreting the pooled estimates. This heterogeneity may also partly explain the lack of statistically significant differences observed in atrial fibrillation–specific outcomes, despite consistent improvements in broader arrhythmia‐related endpoints.

The methodologies employed in the included studies varied significantly, from comprehensive bi‐atrial ablation as reported by Yu et al. [5] to left‐sided techniques, including the Convergent method, the thoracoscopic box lesion approach in CEASE‐AF, and the phased processes outlined by Hwang et al. [1, 2, 4, 5, 14]. These techniques represent distinct therapeutic approaches with differing anticipated arrhythmias and safety profiles. Bi‐atrial procedures may facilitate more extensive substrate modification, albeit at the expense of greater invasiveness; in contrast, modern left atrial‐only hybrid methods are more standardized and widely adopted.

Furthermore, the timing varied significantly across studies. Some individuals underwent HA as a single integrated treatment, while others completed HA in stages, delaying the endocardial components for several weeks to facilitate lesion maturation and minimize tissue edema. This variation impacts procedural risks, lesion longevity, and the capacity to detect arrhythmias in the short term, thereby complicating direct comparisons across studies. Additionally, the extent of follow‐up monitoring differed among studies, which is known to influence arrhythmia detection rates.

The discrepancies in the CA comparator arm further increased heterogeneity. Some studies incorporated multiple endocardial lesion sets, including posterior wall isolation, mitral and cavotricuspid isthmus ablation, or substrate modification through various methods. Different studies predominantly utilized pulmonary vein isolation with optional lines [4, 5, 12, 14]. These variations further enhance the disparity between HA and CA. When CA is applied to many lesion sets, the added benefit of HA may not be as pronounced; however, restricted CA techniques might make HA appear more successful than it truly is.

Another aspect of variability arises from differences in epicardial access routes and energy modalities. Certain hybrid procedures use thoracoscopic bipolar radiofrequency systems, which are considered the most reliable method for creating transmural surgical lesions. In contrast, others employ subxiphoid or transdiaphragmatic access with unipolar vacuum‐assisted probes, as typically seen in the Convergent procedure (e.g., DeLurgio et al.) [4, 6]. The depth and uniformity of the lesions, along with the risk profiles associated with these treatments, vary significantly.

Therefore, the procedural features varied significantly across studies, contributing to the substantial differences between the primary and secondary results. The sensitivity analyses conducted in our review corroborated this finding: the study by Edgerton et al., which utilized a nonstandard manual epicardial technique, disproportionately influenced the pooled estimates. However, the effects observed in the studies by Maclean and Edgerton were markedly different from those in CEASE‐AF and the research conducted by Kress et al., highlighting the variability of hybrid strategies [1, 2, 6, 13].

In addition, safety results demonstrate this difference. Although HA improved rhythm outcomes, the pooled research indicated more significant complications. This is probably associated with the invasiveness of the surgical component and differences between the procedures, rather than any inherent issues with the hybrid technique itself. Recent standardized randomized studies, such as CONVERGE and CEASE‐AF, found that complication rates were comparable for HA and CA when experienced multidisciplinary teams employed the same workflows. These data suggest that the hybrid technique has evolved and that safety concerns may be mitigated in specialized facilities.

Nonetheless, this study had several limitations that warrant attention. An additional important limitation relates to the inherent risk of bias across the included studies. Blinding is particularly challenging in hybrid ablation due to its surgical component, which may introduce both performance and detection bias. This is especially relevant to outcomes that depend on rhythm monitoring and clinical decision‐making, such as arrhythmia recurrence and repeat interventions. Consequently, outcome assessment may be influenced by differential follow‐up intensity or clinician expectations, potentially leading to an overestimation of treatment effects in favor of hybrid strategies. Furthermore, non‐randomized studies remain susceptible to residual confounding despite methodological adjustments, particularly in patient selection and procedural allocation. The considerable diversity of hybrid techniques, energy sources, access routes, and CA comparators constrains the accuracy of aggregated estimations. The inclusion of both randomized and observational studies increases the risk of bias; however, the subgroup analyses did not reveal any consistent differences in treatment effects when stratified by research type. The paucity of clinical trials, due to the complexity of the procedures under investigation and discrepancies in rhythm‐monitoring methodologies, has significantly impacted the results. Finally, the relatively small number of included studies and overall sample size may limit the statistical power of the meta‐analysis, particularly for less frequent outcomes. Although funnel plot inspection did not suggest substantial asymmetry, the interpretation of small‐study effects remains inherently limited in analyses with few studies. Nevertheless, the consistency of findings across sensitivity analyses and the absence of influential outliers support the overall robustness of the results. Importantly, the definition of repeat ablation varied across studies and did not consistently distinguish between planned staged procedures—commonly inherent to hybrid strategies—and unplanned reinterventions due to treatment failure. This limitation may have biased the pooled estimate in either direction or reduced the interpretability of this endpoint. Therefore, caution is warranted when interpreting the observed reduction in repeat ablation rates.

Further research should concentrate on standardizing hybrid methodologies, providing comprehensive procedural documentation, and employing consistent definitions of success and monitoring approaches. To clarify comparative safety and identify individuals who might benefit most from HA, particularly those with significant atrial enlargement or longstanding AF, large‐scale, multicenter randomized studies with long‐term follow‐up are necessary. As HA becomes increasingly prevalent in specialized AF programs, enhanced patient selection and procedural uniformity will be crucial for maximizing its clinical value.

In conclusion, for patients with non‐paroxysmal AF, HA offers superior rhythm control compared to endocardial CA alone. Specifically, HA is associated with significantly higher rates of freedom from any arrhythmia and freedom from AADs. However, this improved efficacy comes with an increased risk of significant complications, as indicated by pooled data, as well as longer procedure times and hospital stays. Importantly, recent randomized controlled trials have demonstrated comparable safety profiles, suggesting that in experienced centers with a dedicated heart team, these risks may be mitigated. Therefore, HA constitutes an effective therapeutic strategy for this challenging patient population but requires a patient‐centered approach that carefully weighs the substantial rhythm benefits against the procedural risks and complexities.

## Author Contributions

Conceptualization: A.S.M.J.; B.R.S.; A.V.B. and P.L.A.A. Search and data extraction: B.R.S.; A.V.B.; A.S.M.J. Statistical analysis: B.R.S.; A.S.M.J.; A.V.B. and P.L.A.A. Writing – original draft: A.V.B.; B.R.S.; A.S.M.J.; A.F.F.J.; S.M.B. Writing – review and editing: All authors Supervision: A.S.M.J.

## Funding

This research received no external funding.

## Conflicts of Interest

The authors declare no conflicts of interest.

## Supporting information


**Figure S1:** Flow chart of selected studies.
**Table S1:** Main Inclusion and Exclusion Criteria of Included Studies.
**Table S2:** Definitions of Hybrid Ablation and Endocardial Ablation of Included Studies.
**Methods S1:** PRISMA 2020 Checklist.
**Methods S2:** PRISMA 2020 Checklist for Abstracts.
**Methods S3:** Details of Search Strategies.
**Figure S1A:** Leave‐One‐Out Sensitivity Analysis for Freedom from Atrial Fibrillation.
**Figure S1B:** Leave‐One‐Out Sensitivity Analysis for Freedom from Anti‐Arrhythmic Drug (AAD).
**Figure S1C:** Leave‐One‐Out Sensitivity Analysis for Freedom from Arrhythmia (Regardless of AADs).
**Figure S1D:** Leave‐One‐Out Sensitivity Analysis for Repeat Ablation.
**Figure S1E:** Leave‐One‐Out Sensitivity Analysis for Arrhythmia Recurrence.
**Figure S2A:** Baujat Plot for Freedom from Atrial Fibrillation.
**Figure S2B:** Baujat Plot for Freedom from Anti‐Arrhythmic Drug (AAD).
**Figure S2C:** Baujat Plot for Freedom from Arrhythmia (Regardless of AADs).
**Figure S2D:** Baujat Plot for Arrhythmia Recurrence.
**Figure S2E:** Baujat Plot for Repeat Ablation.
**Figure S3A:** Funnel Plot for Freedom from Atrial Fibrillation.
**Figure S3B:** Funnel Plot for Freedom from Anti‐Arrhythmic Drug (AAD).
**Figure S3C:** Funnel Plot for Freedom from Arrhythmia (Regardless of AADs).
**Figure S3D:** Funnel Plot for Arrhythmia Recurrence.
**Figure S3E:** Funnel Plot for Repeat Ablation.
**Figure S4A:** Subgroup Analysis of Type of energy for Freedom from Atrial Fibrillation.
**Figure S4B:** Subgroup Analysis of Type of Energy for Freedom from Anti‐Arrhythmic Drug (AAD).
**Figure S4C:** Subgroup Analysis of Type of energy for Freedom from Arrhythmia (Regardless of AADs).
**Figure S4D:** Subgroup Analysis of the Type of Energy for Arrhythmia Recurrence.
**Figure S4E:** Subgroup Analysis of Type of Energy for Repeat Ablation.
**Figure S5A:** Subgroup Analysis of the Type of Study for Freedom from Atrial Fibrillation.
**Figure S5B:** Subgroup Analysis of the Type of Study for Freedom from Anti‐Arrhythmic Drug (AAD).
**Figure S5C:** Subgroup Analysis of Type of Study for Freedom from Arrhythmia (Regardless of AADs).
**Figure S5D:** Subgroup Analysis of the Type of Study for Arrhythmia Recurrence.
**Figure S5E:** Subgroup Analysis of the Type of Study for Repeat Ablation.
**Figure S6A:** Meta regression analysis of Age for Freedom from Atrial Fibrillation.
**Figure S6B:** Meta regression analysis of Age for Freedom from Anti‐Arrhythmic Drug (AAD).
**Figure S6C:** Meta regression analysis of Age for Freedom from Arrhythmia (Regardless of AADs).
**Figure S6D:** Meta regression analysis of Age for Arrhythmia Recurrence.
**Figure S6E:** Meta regression analysis of Age for Repeat Ablation.
**Figure S7A:** Meta regression analysis of Male sex for Freedom from Atrial Fibrillation.
**Figure S7B:** Meta regression analysis of Male sex for Freedom from Anti‐Arrhythmic Drug (AAD).
**Figure S7C:** Meta regression analysis of Male sex for Freedom from Arrhythmia (Regardless of AADs).
**Figure S7D:** Meta regression analysis of Male sex for Arrhythmia Recurrence.
**Figure S7E:** Meta regression analysis of Male sex for Repeat Ablation.
**Figure S8A:** Meta regression analysis of LVEF for Freedom from Atrial Fibrillation.
**Figure S8B:** Meta regression analysis of LVEF for Freedom from Anti‐Arrhythmic Drug (AAD).
**Figure S8C:** Meta regression analysis of LVEF for Freedom from Arrhythmia (Regardless of AADs).
**Figure S8D:** Meta regression analysis of LVEF for Arrhythmia Recurrence.
**Figure S8E:** Meta regression analysis of LVEF for Repeat Ablation.
**Figure S9A:** Meta regression analysis of Duration of AF for Freedom from Atrial Fibrillation.
**Figure S9B:** Meta regression analysis of Duration of AF for Freedom from Anti‐Arrhythmic Drug (AAD).
**Figure S9C:** Meta regression analysis of Duration of AF for Freedom from Arrhythmia (Regardless of AADs).
**Figure S9D:** Meta regression analysis of Duration of AF for Arrhythmia Recurrence.
**Figure S10A:** Forest Plot for Cardiac Tamponade.
**Figure S10B:** Forest Plot for Cardioversion.
**Figure S10C:** Forest Plot for Death.
**Figure S10D:** Forest Plot for Major Complications.
**Figure S10E:** Forest Plot for Phrenic nerve paralysis.
**Figure S10F:** Forest Plot for Stroke.
**Figure S11A:** Leave‐One‐Out Sensitivity Analysis for Cardiac Tamponade.
**Figure S11B:** Leave‐One‐Out Sensitivity Analysis for Cardioversion.
**Figure S11C:** Leave‐One‐Out Sensitivity Analysis for Death.
**Figure S11D:** Leave‐One‐Out Sensitivity Analysis for Major Complications.
**Figure S11E:** Leave‐One‐Out Sensitivity Analysis for Phrenic nerve paralysis.
**Figure S11F:** Leave‐One‐Out Sensitivity Analysis for Stroke.
**Figure S12A:** Baujat Plot for Cardiac Tamponade.
**Figure S12B:** Baujat Plot for Cardioversion.
**Figure S12C:** Baujat Plot for Death.
**Figure S12D:** Baujat Plot for Major Complications.
**Figure S12E:** Baujat Plot for Phrenic nerve paralysis.
**Figure S12F:** Baujat Plot for Stroke.
**Figure S13A:** Funnel Plot for Cardiac Tamponade.
**Figure S13B:** Funnel Plot for Cardioversion.
**Figure S13C:** Funnel Plot for Death.
**Figure S13D:** Funnel Plot for Major Complications.
**Figure S13E:** Funnel Plot for Phrenic nerve paralysis.
**Figure S13F:** Funnel Plot for Stroke.
**Figure S14A:** Forest Plot for Fluoroscopy time, minutes.
**Figure S14B:** Forest Plot for Length of stay, days.
**Figure S14C:** Forest Plot for Procedure time for the endocardial ablation, minutes.
**Figure S14D:** Forest Plot for Total procedure duration (minutes).
**Figure S15A:** Leave‐One‐Out Sensitivity Analysis for Fluoroscopy time, minutes.
**Figure S15B:** Leave‐One‐Out Sensitivity Analysis for Length of stay, days.
**Figure S15C:** Leave‐One‐Out Sensitivity Analysis for Total procedure duration (minutes).
**Figure S16A:** Subgroup Analysis of type of study for Fluoroscopy time, minutes.
**Figure S16B:** Subgroup Analysis of type of study for Length of stay, days.
**Figure S16C:** Subgroup Analysis of the type of study for Total procedure duration (minutes).
**Figure S17A:** Baujat Plot for Fluoroscopy time, minutes.
**Figure S17B:** Baujat Plot for Length of stay, days.
**Figure S17C:** Baujat Plot for Total procedure duration (minutes).
**Figure S18A:** Traffic Light Plot of ROBINS‐I Assessment.
**Figure S18B:** Summary Bar Plot of ROBINS‐I Assessment.
**Figure S19C:** Traffic Light Plot of RoB2 Assessment.
**Figure S19D:** Summary Bar Plot of ROBINS‐2 Assessment.

## Data Availability

Data supporting the findings of this systematic review and meta‐analysis are included in this article and the related Supporting Information. Additional raw data extracted from the included studies are available upon request from the corresponding author (ASMJ).
