# Supplementary material for: Hybrid Thoracoscopic‐Endocardial Versus Endocardial Catheter Ablation for Persistent Atrial Fibrillation: A Systematic Review and Meta‐Analysis
Source: J Arrhythm. 2026 Jul 10;42(4):e70416. doi: 10.1002/joa3.70416 (PMC13354847; doi:10.1002/joa3.70416)

**Supplementary Materials**

**Hybrid Thoracoscopic-Endocardial vs. Endocardial Catheter Ablation for Persistent Atrial Fibrillation: A Systematic Review and Meta-Analysis**

Annelyse Vitória Barbosa^1^, Bárbara Reis Silva^1^, Pedro Lucas Alves Alencar^2^, Silvia Marçal Botelho M.D., Ph.D.^2^, Aguinaldo Figueredo Freitas Junior, M.D., Ph.D. FACC, FESC^2^ and Antônio da Silva Menezes Júnior, MD, PhD, FESC ^1,2,†^

^1^Internal Medicine Department, Medical Sciences and Life School, Pontifical Catholic University of Goiás, Goiânia, Goiás, Brazil.

^2^ Faculty of Medicine, Internal Medicine Department, Federal University of Goiás, Goiânia, Goiás, Brazil.

Contents

[Supplemental Methods 1. PRISMA 2020 Checklist 3](#_Toc1818175660)

[Supplemental Methods 2. PRISMA 2020 Checklist for Abstracts 7](#_Toc723275563)

[Supplemental Methods 3. Details of Search Strategies 9](#_Toc1172450797)

Supplemental Figure 1. Flow chart of selected studies………………………………………………………….………………………………………………………………….…10

[Supplemental Table 1. Main Inclusion and Exclusion Criteria of Included Studies 10](#_Toc1185762860)

[Supplemental Table 2. Definitions of Hybrid Ablation and Endocardial Ablation of Included Studies 13](#_Toc1792338030)

[Supplemental Results 18](#_Toc1414030318)

[Supplemental Results . Risk of Bias Assessment of Included Studies 97](#_Toc1986769710)

## Supplemental Methods 1. PRISMA 2020 Checklist

| **Section and Topic** | **Item #** | **Checklist item** | **Location where item is reported** |
| --- | --- | --- | --- |
| **TITLE** | | |  |
| Title | 1 | Identify the report as a systematic review. | Pg. 1 at MS |
| **ABSTRACT** | | |  |
| Abstract | 2 | See the PRISMA 2020 for Abstracts checklist. | Suppl. methods 2 |
| **INTRODUCTION** | | |  |
| Rationale | 3 | Describe the rationale for the review in the context of existing knowledge. | Pg. 3 at MS |
| Objectives | 4 | Provide an explicit statement of the objective(s) or question(s) the review addresses. | Pg. 3 at MS |
| **METHODS** | | |  |
| Eligibility criteria | 5 | Specify the inclusion and exclusion criteria for the review and how studies were grouped for the syntheses. | Pg. 4 at MS |
| Information sources | 6 | Specify all databases, registers, websites, organizations, reference lists, and other sources searched or consulted to identify studies. Specify the date when each source was last searched or consulted. | Pg. 4 at MS |
| Search strategy | 7 | Present the full search strategies for all databases, registers, and websites, including any filters and limits used. | Pg. 4 at MS |
| Selection process | 8 | Specify the methods used to decide whether a study met the inclusion criteria of the review, including how many reviewers screened each record and each report retrieved, whether they worked independently, and if applicable, details of automation tools used in the process. | Pg. 4 at MS |
| Data collection process | 9 | Specify the methods used to collect data from reports, including how many reviewers collected data from each report, whether they worked independently, any processes for obtaining or confirming data from study investigators, and if applicable, details of automation tools used in the process. | Pg. 4 at MS |
| Data items | 10a | List and define all outcomes for which data were sought. Specify whether all results that were compatible with each outcome domain in each study were sought (e.g. for all measures, time points, analyses), and if not, the methods used to decide which results to collect. | Pg. 4 at MS |
|  | 10b | List and define all other variables for which data were sought (e.g. participant and intervention characteristics, funding sources). Describe any assumptions made about any missing or unclear information. | Pg. 4 at MS |
| Study risk of bias assessment | 11 | Specify the methods used to assess risk of bias in the included studies, including details of the tool(s) used, how many reviewers assessed each study, and whether they worked independently, and if applicable, details of automation tools used in the process. | Pg. 4 at MS |
| Effect measures | 12 | Specify for each outcome the effect measure(s) (e.g., risk ratio, mean difference) used in the synthesis or presentation of results. | Pg. 5 at MS |
| Synthesis methods | 13a | Describe the processes used to decide which studies were eligible for each synthesis (e.g., tabulating the study intervention characteristics and comparing against the planned groups for each synthesis (item #5)). | Pg. 4-7 at MS |
|  | 13b | Describe any methods required to prepare the data for presentation or synthesis, such as handling of missing summary statistics or data conversions. | Pg. 4-7 at MS |
|  | 13c | Describe any methods used to tabulate or visually display the results of individual studies and syntheses. | Pg. 4-7 at MS |
|  | 13d | Describe any methods used to synthesize results and provide a rationale for the choice(s). If meta-analysis was performed, describe the model(s), method(s) to identify the presence and extent of statistical heterogeneity, and software package(s) used. | Pg. 4-7 at MS |
|  | 13e | Describe any methods used to explore possible causes of heterogeneity among study results (e.g., subgroup analysis, meta-regression). | Pg. 4-7 at MS |
|  | 13f | Describe any sensitivity analyses conducted to assess the robustness of the synthesized results. | Pg. 4-7 at MS |
| Reporting bias assessment | 14 | Describe any methods used to assess the risk of bias due to missing results in a synthesis (arising from reporting biases). | Pg. 7 at MS |
| Certainty assessment | 15 | Describe any methods used to assess certainty (or confidence) in the body of evidence for an outcome. | NA |
| **RESULTS** | | |  |
| Study selection | 16a | Describe the results of the search and selection process, from the number of records identified in the search to the number of studies included in the review, ideally using a flow diagram. | Figure 1 |
|  | 16b | Cite studies that might appear to meet the inclusion criteria, but which were excluded, and explain why they were excluded. | NA |
| Study characteristics | 17 | Cite each included study and present its characteristics. | Table 1 and suppl. Tables 1-2 |
| Risk of bias in studies | 18 | Present assessments of risk of bias for each included study. | suppl. results 18-19 |
| Results of individual studies | 19 | For all outcomes, present, for each study: (a) summary statistics for each group (where appropriate) and (b) an effect estimate and its precision (e.g., confidence/credible interval), ideally using structured tables or plots. | Figures 2-3 at MS and suppl. results |
| Results of syntheses | 20a | For each synthesis, briefly summarize the characteristics and risk of bias among contributing studies. | suppl. results 18-19 |
|  | 20b | Present the results of all statistical syntheses conducted. If meta-analysis was done, present for each the summary estimate and its precision (e.g. confidence/credible interval) and measures of statistical heterogeneity. If comparing groups, describe the direction of the effect. | Pg. 5-7 and suppl. results |
|  | 20c | Present the results of all investigations of possible causes of heterogeneity among study results. | Pg. 5-7 at MS and suppl. results |
|  | 20d | Present the results of all sensitivity analyses conducted to assess the robustness of the synthesized results. | Pg. 5-7 at MS and suppl. results |
| Reporting biases | 21 | Present assessments of risk of bias due to missing results (arising from reporting biases) for each synthesis assessed. | NA |
| Certainty of evidence | 22 | Present assessments of certainty (or confidence) in the body of evidence for each outcome assessed. | NA |
| **DISCUSSION** | | |  |
| Discussion | 23a | Provide a general interpretation of the results in the context of other evidence. | Pg. 8-9 at MS |
|  | 23b | Discuss any limitations of the evidence included in the review. | Pg. 9 at MS |
|  | 23c | Discuss any limitations of the review processes used. | Pg. 9 at MS |
|  | 23d | Discuss implications of the results for practice, policy, and future research. | Pg. 9 at MS |
| **OTHER INFORMATION** | | |  |
| Registration and protocol | 24a | Provide registration information for the review, including register name and registration number, or state that the review was not registered. | PROSPERO (CRD42024600526) |
|  | 24b | Indicate where the review protocol can be accessed, or state that a protocol was not prepared. | <https://www.crd.york.ac.uk/PROSPERO/view/CRD42024600526>. |
|  | 24c | Describe and explain any amendments to information provided at registration or in the protocol. | NA |
| Support | 25 | Describe sources of financial or non-financial support for the review, and the role of the funders or sponsors in the review. | NA |
| Competing interests | 26 | Declare any competing interests of review authors. | NA |
| Availability of data, code, and other materials | 27 | Report which of the following are publicly available and where they can be found: template data collection forms; data extracted from included studies; data used for all analyses; analytic code; any other materials used in the review. | NA |

*MS:* Manuscript; *Suppl.:* Supplementary Material

## Supplemental Methods 2. PRISMA 2020 Checklist for Abstracts

| **Section and Topic** | **Item #** | **Checklist item** | **Reported (Yes/No)** |
| --- | --- | --- | --- |
| **TITLE** | | |  |
| Title | 1 | Identify the report as a systematic review. | **Yes** |
| **BACKGROUND** | | |  |
| Objectives | 2 | Provide an explicit statement of the main objective(s) or question(s) the review addresses. | **Yes** |
| **METHODS** | | |  |
| Eligibility criteria | 3 | Specify the inclusion and exclusion criteria for the review. | **Yes** |
| Information sources | 4 | Specify the information sources (e.g. databases, registers) used to identify studies and the date when each was last searched. | **Yes** |
| Risk of bias | 5 | Specify the methods used to assess risk of bias in the included studies. | **Yes** |
| Synthesis of results | 6 | Specify the methods used to present and synthesize results. | **Yes** |
| **RESULTS** | | |  |
| Included studies | 7 | Give the total number of included studies and participants, and summarize relevant characteristics of studies. | **Yes** |
| Synthesis of results | 8 | Present results for primary outcomes, preferably indicating the number of studies included and participants for each. If a meta-analysis was done, report the summary estimate and confidence/credible interval. If comparing groups, indicate the direction of the effect (i.e., which group is favored). | **Yes** |
| **DISCUSSION** | | |  |
| Limitations of evidence | 9 | Provide a summary of the limitations of the evidence included in the review (e.g. study risk of bias, inconsistency and imprecision). | **Yes** |
| Interpretation | 10 | Provide a general interpretation of the results and important implications. | **Yes** |
| **OTHER** | | |  |
| Funding | 11 | Specify the primary source of funding for the review. | **Yes** |
| Registration | 12 | Provide the register name and registration number. | **Yes** |

## Supplemental Methods 3. Details of Search Strategies

| **Database** | **Search Strategy** |
| --- | --- |
| PubMed  (387 results) | (“atrial fibrillations” OR “atrial fibrillation” OR “persistent atrial fibrillation” OR “persistent atrial fibrillations” OR “long-standing persistent atrial fibrillation” ) AND (“hybrid ablation” OR “hybrid” OR “hybrid ablations” OR “hybrid endocardial-epicardial ablation” OR “hybrid endocardial epicardial ablation” OR “hybrid epicardial-endocardial ablation” OR “hybrid epicardial endocardial ablation” OR “hybrid therapy”) AND (“catheter ablation” OR “transvenous electric ablation” OR “transvenous electrical ablation” OR “electric catheter ablation” OR “electrical catheter ablation” OR “percutaneous catheter ablation” OR “endocardial ablation” OR “radiofrequency catheter ablation” OR “repeated catheter ablation” OR “transvenous catheter ablation”) |
| Embase  (760 results) | ('atrial fibrillations' OR 'atrial fibrillation'/exp OR 'atrial fibrillation' OR 'persistent atrial fibrillation'/exp OR 'persistent atrial fibrillation' OR 'persistent atrial fibrillations' OR 'long standing persistent atrial fibrillation'/exp OR 'long standing persistent atrial fibrillation') AND ('hybrid ablation'/exp OR 'hybrid ablation' OR 'hybrid'/exp OR 'hybrid' OR 'hybrid ablations' OR 'hybrid endocardial-epicardial ablation' OR 'hybrid endocardial epicardial ablation' OR 'hybrid epicardial-endocardial ablation' OR 'hybrid epicardial endocardial ablation' OR 'hybrid therapy'/exp OR 'hybrid therapy') AND ('catheter ablation'/exp OR 'catheter ablation' OR 'transvenous electric ablation' OR 'transvenous electrical ablation' OR 'electric catheter ablation' OR 'electrical catheter ablation' OR 'percutaneous catheter ablation' OR 'endocardial ablation'/exp OR 'endocardial ablation' OR 'radiofrequency catheter ablation'/exp OR 'radiofrequency catheter ablation' OR 'repeated catheter ablation' OR 'transvenous catheter ablation') |
| Cochrane Library  (63 results) | (“atrial fibrillations” OR “atrial fibrillation” OR “persistent atrial fibrillation” OR “persistent atrial fibrillations” OR “long standing persistent atrial fibrillation” ) AND (“hybrid ablation” OR “hybrid” OR “hybrid ablations” OR “hybrid endocardial-epicardial ablation” OR “hybrid endocardial epicardial ablation” OR “hybrid epicardial-endocardial ablation” OR “hybrid epicardial endocardial ablation” OR “hybrid therapy”) AND (“catheter ablation” OR “transvenous electric ablation” OR “transvenous electrical ablation” OR “electric catheter ablation” OR “electrical catheter ablation” OR “percutaneous catheter ablation” OR “endocardial ablation” OR “radiofrequency catheter ablation” OR “repeated catheter ablation” OR “transvenous catheter ablation”) |

## Supplemental Figure 1. Flow chart of selected studies.


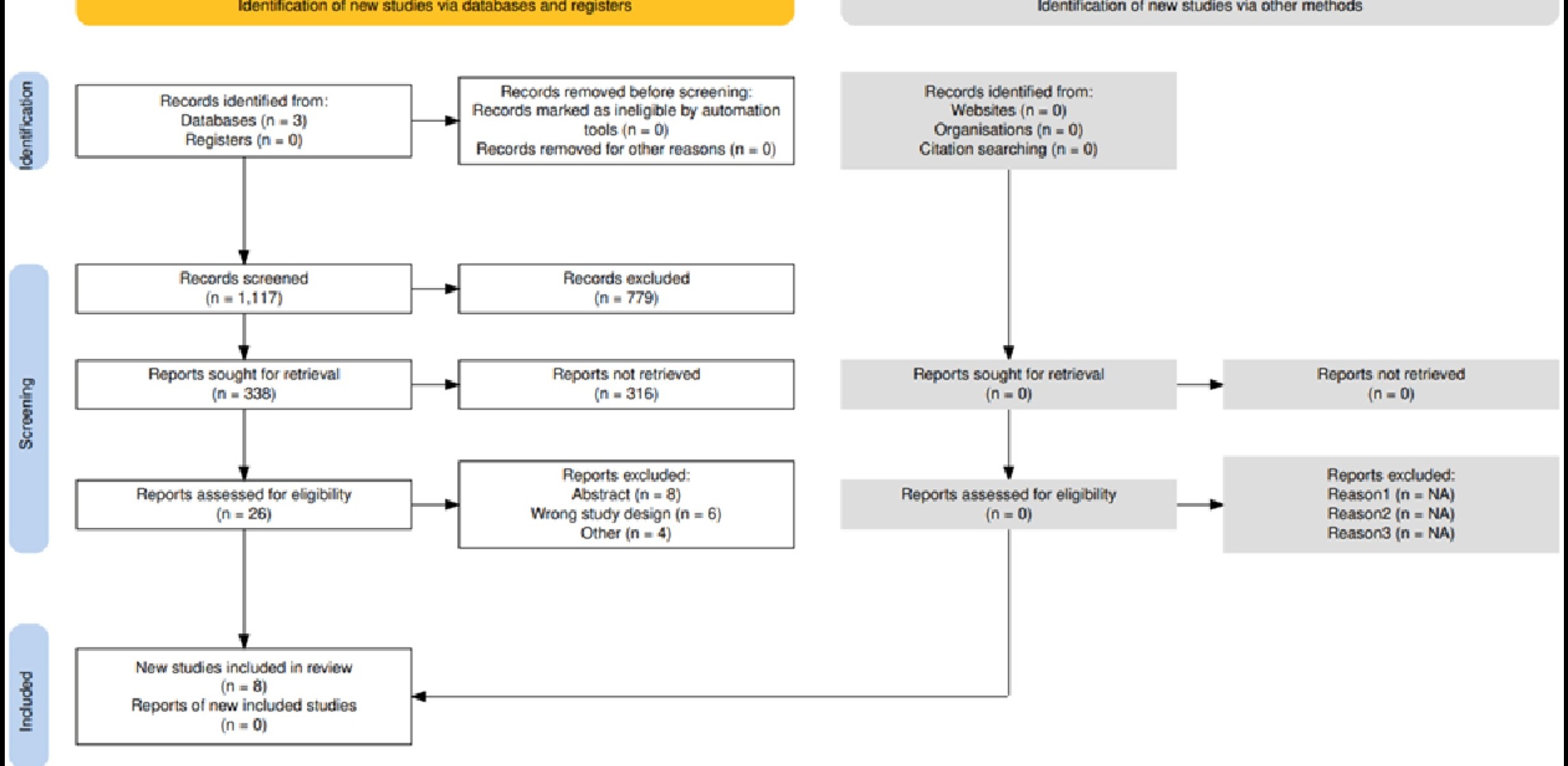


## Supplemental Table 1. Main Inclusion and Exclusion Criteria of Included Studies

| **Study** | **Inclusion Criteria** | **Exclusion Criteria** |
| --- | --- | --- |
| **Chunyu, 2024** | Diagnosis of nonparoxysmal atrial fibrillation (NPAF).Left atrial diameter (LAD) ≥ 45 mm. No previous failed catheter ablation. | Patients who had previously failed catheter ablation (i.e., redo patients were excluded from the initial cohort selection, and left atrial diameter < 45 mm—paroxysmal atrial fibrillation (only NPAF patients were included). |
| **Van der Heijden, 2023** | Age >18 years, symptomatic (long-standing)-  persAF refractory to 1 or more Vaughan-Williams  class I or III antiarrhythmic drugs and no prior  (catheter) ablation. | Left atrial(LA) diameter >60 mm, contraindications for ablation or prior ablation, body mass index >40 kg/m2, history of cardiac surgery, life expectancy <12 months, and pregnancy. |
| **CEASE-AF,2023** | Between 18 and 75 years of age; had a history of symptomatic PersAF and a LA diameter (LAD) > 4.0 cm or symptomatic LSPAF; and had failed at least one class I or III AAD. Persistent AF was defined as continuous AF sustained beyond seven days, or  lasting greater than 48 h and less than 7 days but necessitating pharmacologic or electrical cardioversion. Long-standing persistent AF was continuous AF lasting more than 12 months. | Key exclusion criteria were: previous ablation procedure; paroxysmal AF; LSPAF > 10 years; AF secondary to electrolyte imbalance, thyroid disease, or other reversible cause; need for  other cardiac surgery procedures besides AF treatment;  or contraindication for CA or epicardial ablation. |
| **Converge,2020** | Age ≥18 years; < 80 years. Documentation of persistent AF Refractory or intolerant to one AAD (class I and/or III) Left atrium < 6.0 cm (Trans Thoracic Echo – TTE – parasternal 4 chamber view). Provided written informed consent | Patients requiring concomitant surgery or have had previous cardiac surgery Left ventricular ejection fraction <40% History of pericarditis Previous cerebrovascular accident Presence of active infection or sepsis, esophageal ulcer stricture and varices Patients with renal dysfunction who are not on dialysis (defined as GFR ≤40) Contraindication for anticoagulants Patients who have had a previous left atrial catheter ablation for AF, or are being treated for ventricular arrhythmia Patients with existing ICDs. |
| **Maclean,2020** | Adult patients with longstanding PeAF that underwent subxiphoid endoscopic ablation of the posterior left atrium followed by catheter ablation. | Only patients with a history of previous cardiac surgery, abdominal surgery, or a contraindication to anticoagulation  were excluded. |
| **Kress,2016** | Had a diagnosis of persistent or long-standing persistent atrial fibrillation (AF) based on the HRS/EHRA/ECAS Consensus Report. | They had paroxysmal AF (only persistent or long-standing persistent AF was included). They lacked complete procedural or follow-up data. |
| **Edgerton,2016** | Patients with symptomatic LSPAF (defined as continuous AF that lasts longer than 1 year17) with evidence of enlarged left atrium (LA) (defined as antero-posterior diameter >4.5 cm), who had failed medical treatment with at least one AAD previously. | Presence of paroxysmal AF, persistent AF for less than 1 year, atrial antero-posterior diameter 4.5 cm, and unwillingness to participate in the study. |
| **Hwang,2018** | Ablation-naive patients. Underwent either a staged hybrid procedure or RFCA alone. Had symptomatic drug-refractory non-valvular AF. Had either persistent AF or long-standing persistent AF. Had been prescribed at least one anti-arrhythmia drug for more than six weeks and had experienced failed medical treatment. | The study excluded patients who had a prior history of ablation for AF. The study also excluded patients with paroxysmal AF. |

## Supplemental Table 2. Definitions of **Hybrid Ablation** and **Endocardial Ablation** of Included Studies

| **Study** | **Definition of Hybrid Ablation** | **Definition of Endocardial Ablation** |
| --- | --- | --- |
| **Yu, 2024** | Epicardial ablation: Access to the pericardial space was achieved through a bilateral transthoracic approach. The left atrial appendage (LAA) was excised by a linear stapler (EZ 60; Ethicon Endosurgery, Cincinnati, OH, USA). The ligament of Marshall was divided by an ultrasonic scalpel. The bipolar radiofrequency clamp (Isolator Synergy Access; AtriCure, Mason, OH, USA) was used to create left pulmonary vein (PV) isolation, left-sided roof and inferior lines, and the left trigone line. The linear lesion connecting the left superior PV to the LAA and the linear lesion connecting the left inferior PV to the great cardiac vein were ablated with the transpolar radiofrequency pen. On the right side, the lesion set included proper PV isolation, ganglion plex ablation at the Waterston groove fat pad, right-sided roof and floor lines, superior vena cava (SVC) to inferior vena cave (IVC) line, right atrial appendage line (the apex to the base at the level of the root of the aorta), and the line connecting the tip of the right atrial appendage to the SVC-IVC line.  Endocardial ablation: performed immediately after thoracoscopic epicardial ablation. Two multipolar catheters were inserted into the coronary sinus (IBI; Abbott, Chicago, IL, USA) and the right ventricle (Daig, Abbott). After transseptal puncture, electroanatomic and voltage mapping were performed using the EnSite NavX mapping system (Abbott). Then the ablation of the coronary sinus, tricuspid isthmus, and mitral isthmus was delivered. | Endocardial ablation: Radiofrequency ablation was used in all patients with the ThermoCool SmartTouch catheter (Biosense Webster, Diamond Bar, CA, USA) or the TactiCath catheter (Abbott). PVI was completed in all patients at first. If AF persisted, in general, the following steps were applied: (1) further stepwise linear ablation in anterior wall,  roof, mitral isthmus, and coronary sinus ablation, cavotriscupid isthmus would be conducted; (2) if AF continued, intravenous ibutilide was administered, followed by elimination of atrial tachycardia under activation and voltage mapping if atrial tachycardia occurred; (3) if AF continued, direct-current cardioversion would be applied. |
| **Van der Heijden, 2023** | Epicardial ablation: 3-port access introduced in the third, fifth, and seventh intercostal spaces. The pericardium was opened posterior to the phrenic nerve with video-assisted thoracoscopic surgery. Isolation of the left pulmonary veins (PV) was performed with a bipolar radiofrequency (RF) clamp (Synergy System, AtriCure). A box lesion connecting the superior and inferior pulmonary veins by creating a roof and inferior line was performed using a unidirectional bipolar RF pen (Coolrail, AtriCure). Thereafter, PV isolation was performed on the right side. In all patients, the left atrial appendage (LAA) was excluded using an epicardial clipping device (AtriClip Pro or Atriclip Pro 2, AtriCure) or an LAA closure device (Lariat, SentreHEART).  Endocardial ablation: After transseptal puncture, exit and  entrance block of all PVs and the box lesion on the  posterior left atrium were evaluated using a 3.5-mm tip ablation catheter (Thermocool Smarttouch, Biosense Webster). | Endocardial ablation: an irrigated tip contact force mapping catheter (ThermoCool Smarttouch, Biosense Webster). Automated lesion tagging (VisiTag, Biosense Webster) was used to mark each ablation lesion. The minimal lesion set performed by endocardial catheter ablation was bilateral pulmonary vein isolation (PVI) and a box lesion excluding  the posterior LA. In cases of previously clinically documented atrial arrhythmias, such as left- or right-sided flutter or ATs, additional ablation was performed in accordance with standard care. |
| **CEASE -AF, 2023** | Epicardial ablation: minimum lesion set of the pulmonary vein  isolation (PVI) and LA posterior wall isolation by means of a “box” lesion using transpolar and bipolar radiofrequency (RF) energy devices (AtriCure Isolator Synergy Clamp [EMR2/EML2] for PVI with pericardial access through bilateral thoracoscopy.  Endocardial ablation: standard techniques by an electrophysiologist between 91 and 180 days following the epicardial index procedure. PVI and posterior box isolation were verified and completed if necessary. | Endocardial ablation: RF catheter technology, including contact force, by an electrophysiologist according to institutional standards. PVI was mandatory during the index procedure. If clinically indicated, a repeat CA was permitted after a 90-day blanking period through 180 days after the endocardial index procedure. |
| **Converge,2020** | Epicardial ablation: vacuum-assisted, unipolar radiofrequency device (EPi-Sense, AtriCure, OH). The right and left PV antrum and contiguous, parallel lesions were made across the posterior wall of the left atrium with pericardial access, transdiaphragmatic or subxiphoid. Endocardial ablation: radiofrequency catheter via standard approach to complete isolation of the PVs. | Endocardial ablation: irrigated radiofrequency catheter to isolate the left and right PVs and connect them via atrial roofline. A cavotriscupid isthmus line was created. |
| **Maclean,2020** | Epicardial ablation: a subxiphoid incision was made, and a transdiaphragmatic approach was used with a laparoscope advanced following carbon dioxide insufflation of the abdomen. The Atricure (OH, USA) EPIsense coagulation catheter with VisiTrax© was used to produce continuous, intersecting linear lesions across the posterior wall of the left atrium. Direct current cardioversion (DCCV) was performed to achieve sinus rhythm if required. Patients recovered in the high dependency unit with a view to discharge after 3 days.  Endocardial ablation was performed approximately 6 weeks later, guided by CARTO 3-D mapping (Biosense Webster Inc., Diamond Bar, CA). The ablation strategy was operator dependent but involved (in all cases) completing PVI and delivering additional lesions to achieve posterior wall isolation, additional CFE ablation (time spent was operator dependent), and finally induction, mapping, and ablation of any atrial tachycardia. In those cases where sinus rhythm was not restored, DCCV was performed. | Endocardial ablation was performed approximately 6 weeks later, guided by CARTO 3-D mapping (Biosense Webster Inc., Diamond Bar, CA). The ablation strategy was operator dependent but involved (in all cases) completing PVI and delivering additional lesions to achieve posterior wall isolation, additional CFE ablation (time spent was operator dependent), and finally induction, mapping, and ablation of any atrial tachycardia. In those cases where sinus rhythm was not restored, DCCV was performed. |
| **Kress,2016** | Epicardial and endocardial ablation: A 2-cm incision was placed in the upper midline, the linea alba was divided, the peritoneum was opened, and a 12-mm port was placed. A Silastic (Dow Corning Corp., Auburn, Michigan) 30-cm pericardioscope was placed through this opening and guided into the oblique sinus with a 6.5-mm scope. Under fluoroscopic guidance, an esophageal temperature probe was passed to lie in the region of the ablation probe. A pre-ablation epicardial map of the posterior LA and proximal pulmonary vein (PV) trunks was created  using the CARTO 3D mapping system (Biosense Webster, South Diamond Bar, California) under direct visualization. Each lesion was delivered with a 3-cm Numeris probe (nContact, Morrisville, North Carolina), which was saline-irrigated and vacuum-attached, at 30 W for 90 s. All lesions were delivered with cold saline infusion within the pericardial sac. Lesions were repeated to achieve 2,700 J delivery, if necessary. The entire posterior LA from the right PV trunk to the left PV trunk was ablated with two rows of parallel overlapping lesions. The anterior left inferior PV trunk was ablated. Lesions were placed in the recess between the inferior vena cava and the right inferior PV in some patients. | Endocardial ablation: Percutaneous radiofrequency catheter ablation targeting pulmonary vein isolation and posterior left atrial substrate modification using electroanatomic mapping. |
| **Edgerton,2016** | Epicardial ablation: the pericardial access was achieved via a transdiaphragmatic approach by means of a subxyphoid incision. The epicardial lesions were performed using an irrigated unipolar RF ablator (Visitrax, nContact Surgical, Morrisville, NC, USA) around the left and right pulmonary veins (PVs), on the posterior wall, the ligament of Marshall (without dissection), and the lateral right atrium. Surgical isolation was verified using a circular mapping catheter (Biosense Webster, Diamond Bar, CA, USA) to assess pulmonary vein entrance and exit block at baseline and during isoproterenol infusion (up to 30 μg/kg for 10–20 minutes). The transseptal puncture was performed under intracardiac echocardiographic guide (AcuNav; Acuson, Mountain View, CA, USA) and after administration of a bolus of unfractionated heparin (10,000 UI for men and 8,000 for women) to maintain an ACT value of 350 seconds.  Endocardial ablation: performed manually. To minimize the risk of complications an esophageal temperature probe was inserted to monitor esophageal temperature during ablation. After the achievement of sinus rhythm, either during ablation or after cardioversion, a pharmacological test with continuous i.v. An infusion of isoproterenol, up to 30 μg/kg for 10–20 minutes, was performed to disclose any non-pulmonary vein triggers or atrial tachycardia and to assess acute pulmonary vein electrical reconnection. | Endocardial ablation: performed manually. To minimize the risk of complications an esophageal temperature probe was inserted to monitor esophageal temperature during ablation. After the achievement of sinus rhythm, either during ablation or after cardioversion, a pharmacological test with continuous i.v. infusion of isoproterenol, up to 30 μg/kg for 10–20 minutes, was performed, to disclose any non-pulmonary vein triggers or atrial tachycardia, and to assess acute pulmonary vein electrical reconnection. |
| **Hwang, 2018** | The staged hybrid procedure for atrial fibrillation (AF) is a sequential combination of two distinct treatments: a totally thoracoscopic ablation (epicardial approach) followed four or five days later by an endocardial radiofrequency catheter ablation (RFCA). The thoracoscopic ablation, which constitutes the surgical part of the procedure, is minimally invasive, using a bipolar radiofrequency clamping device to create a "box lesion" around the pulmonary vein (PV) antrum and superior/inferior lines. It also involves a superior vena cava circular lesion, division of the ligament of Marshall, and ablation of ganglionated plexi (GPs). A key step in the hybrid procedure, performed in 95.8% of the hybrid group, is the removal of the left atrial (LA) appendage. The endocardial RFCA portion is performed subsequently to stabilize the high bleeding tendency and involves catheter-based mapping and ablation to eliminate residual electrical potentials. | Endocardial Ablation: Standalone RF catheter ablation (RFCA) performed via transseptal access, aiming at pulmonary vein isolation and additional linear lesions as needed. |

##

## **Supplemental Results**

Supplemental Figure 1A.Leave-One-Out Sensitivity Analysis for Freedom from Atrial Fibrillation


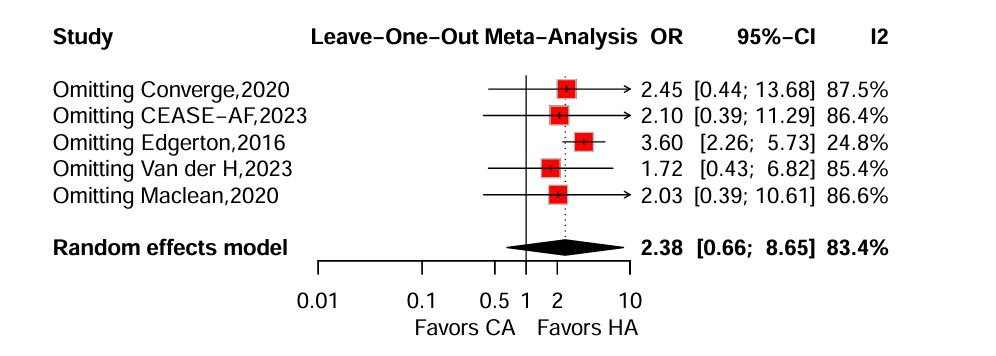


Supplemental Figure 1B. Leave-One-Out Sensitivity Analysis for Freedom from Anti-Arrhythmic Drug (AAD)


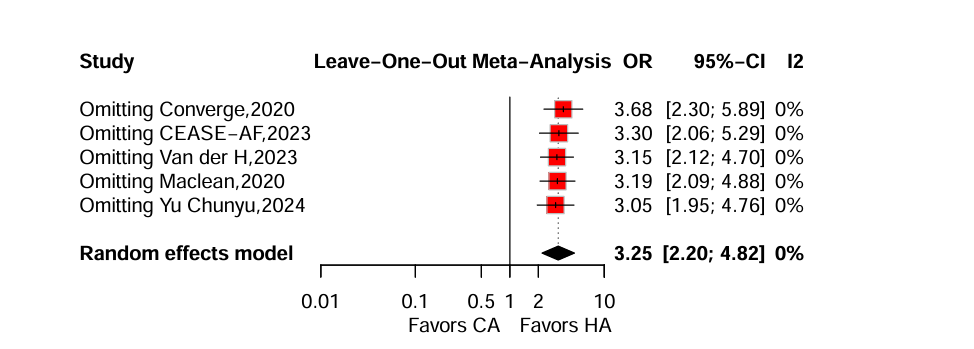


Supplemental Figure 1C. Leave-One-Out Sensitivity Analysis for Freedom from Arrhythmia (Regardless of AADs)


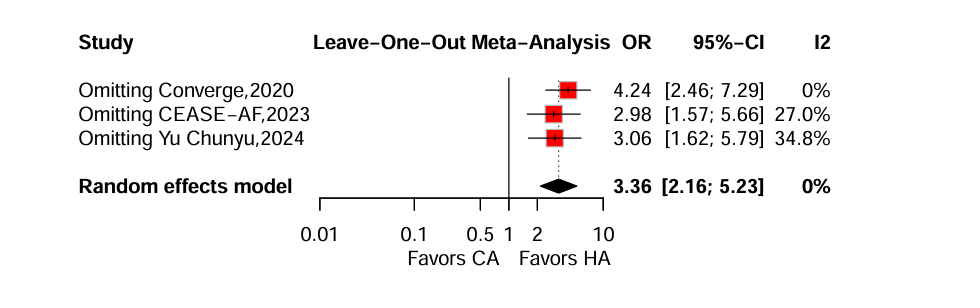


Supplemental Figure 1D. Leave-One-Out Sensitivity Analysis for Repeat Ablation


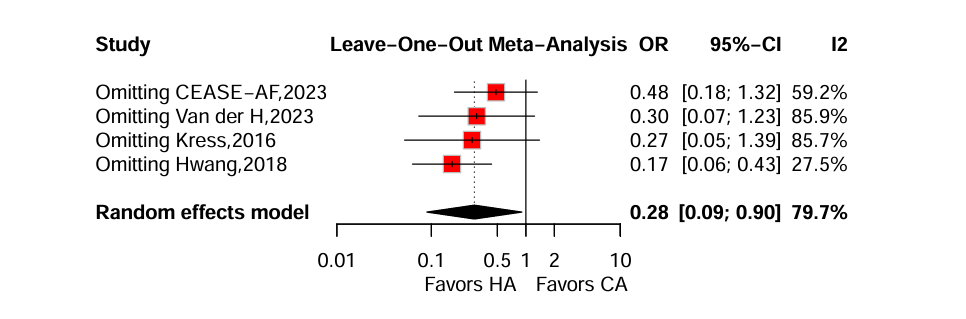


Supplemental Figure 1E. Leave-One-Out Sensitivity Analysis for Arrhythmia Recurrence


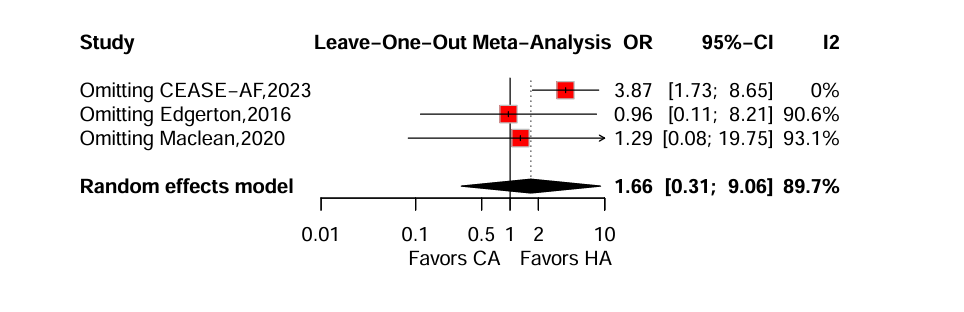


Supplemental Figure 2A. Baujat Plot for Freedom from Atrial Fibrillation


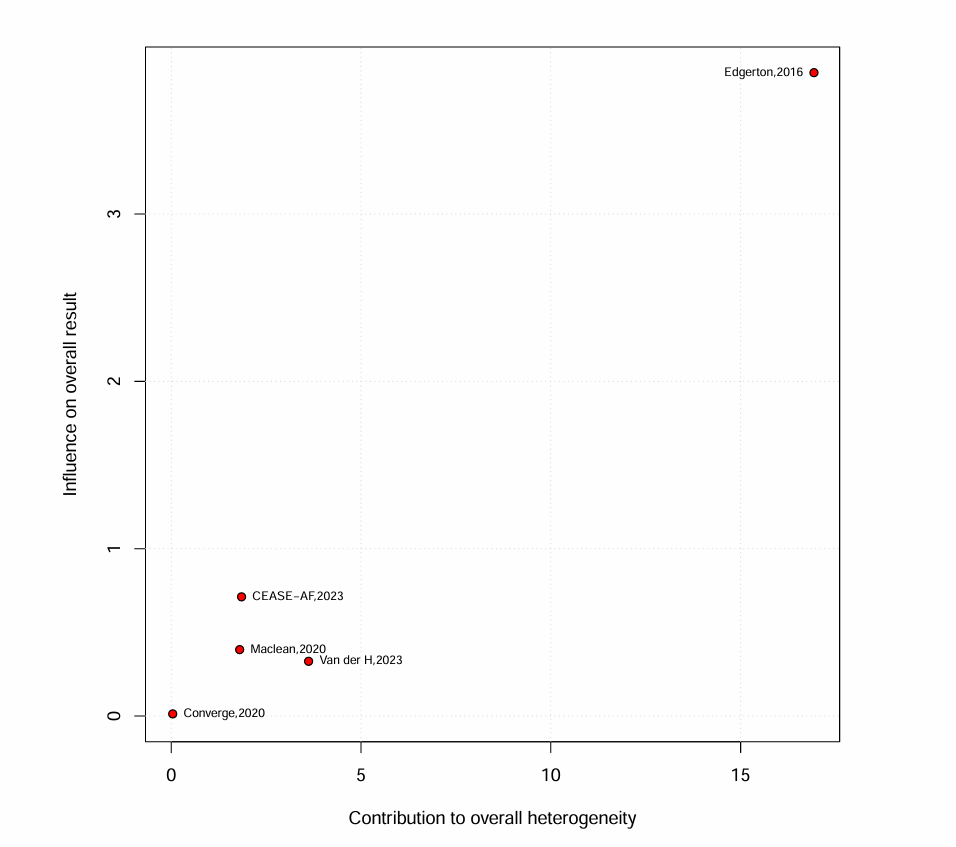


Supplemental Figure 2B. Baujat Plot for Freedom from Anti-Arrhythmic Drug (AAD)


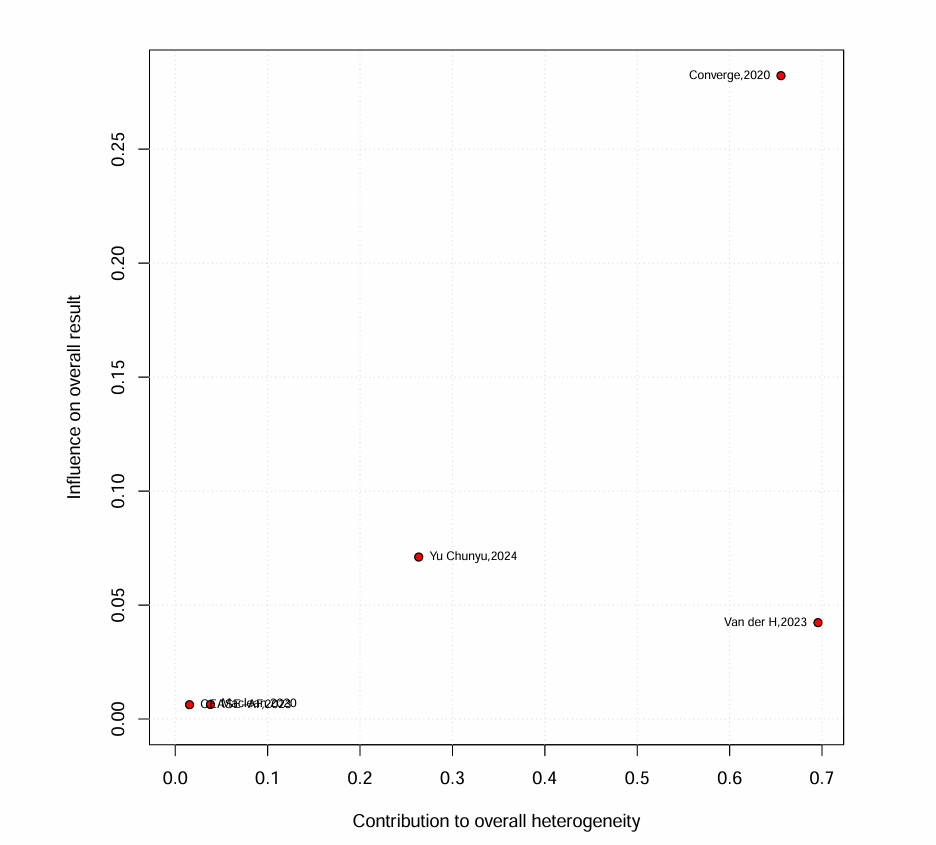


Supplemental Figure 2C. Baujat Plot for Freedom from Arrhythmia (Regardless of AADs)


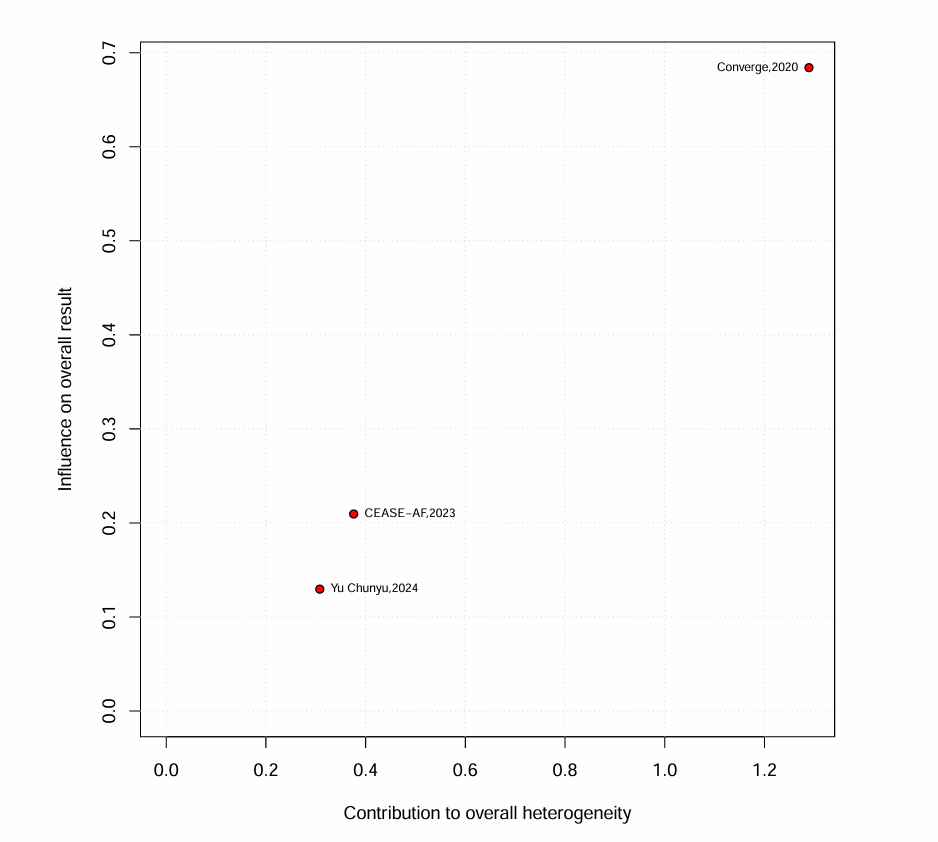


Supplemental Figure 2D. Baujat Plot for Arrhythmia Recurrence


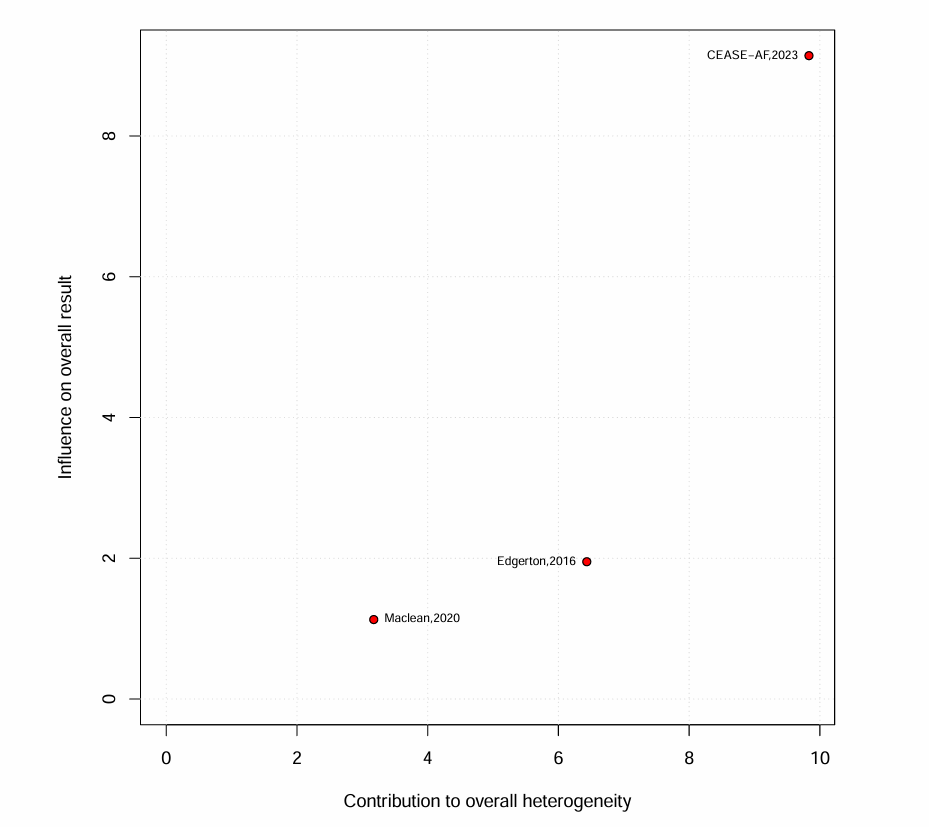


Supplemental Figure 2E. Baujat Plot for Repeat Ablation


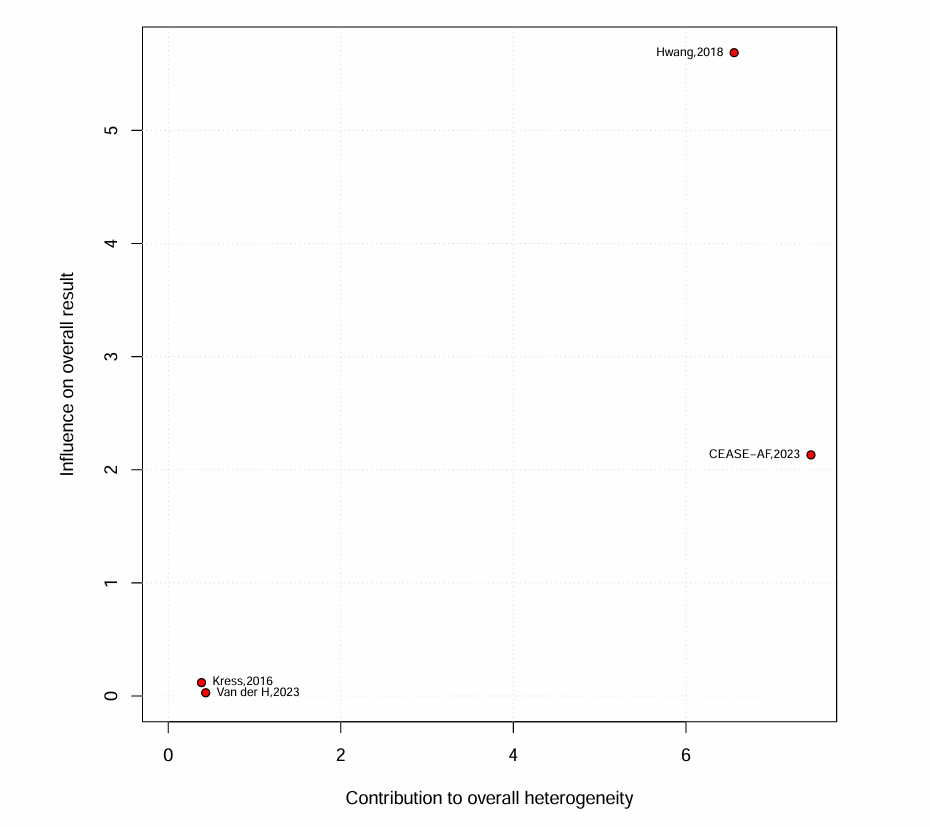


Supplemental Figure 3A.Funnel Plot for Freedom from Atrial Fibrillation


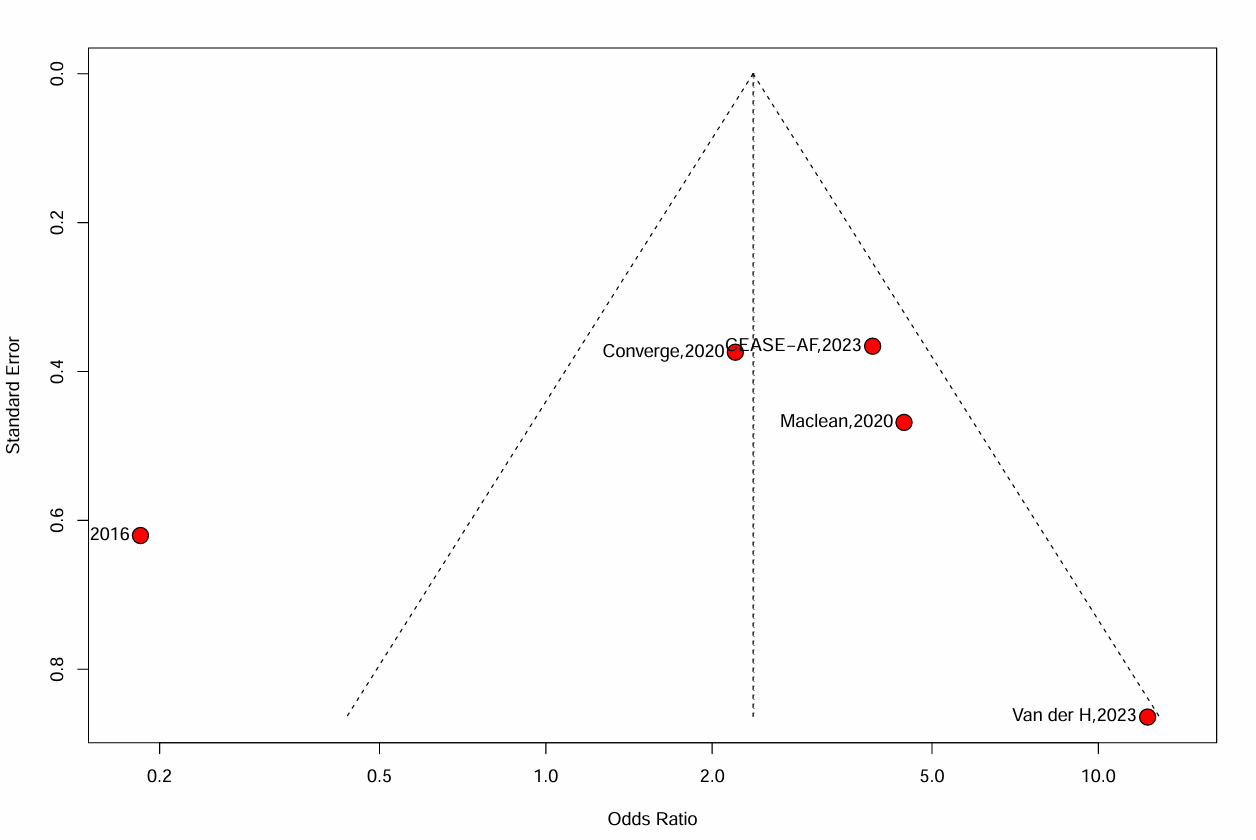


Supplemental Figure 3B.Funnel Plot for Freedom from Anti-Arrhythmic Drug (AAD)


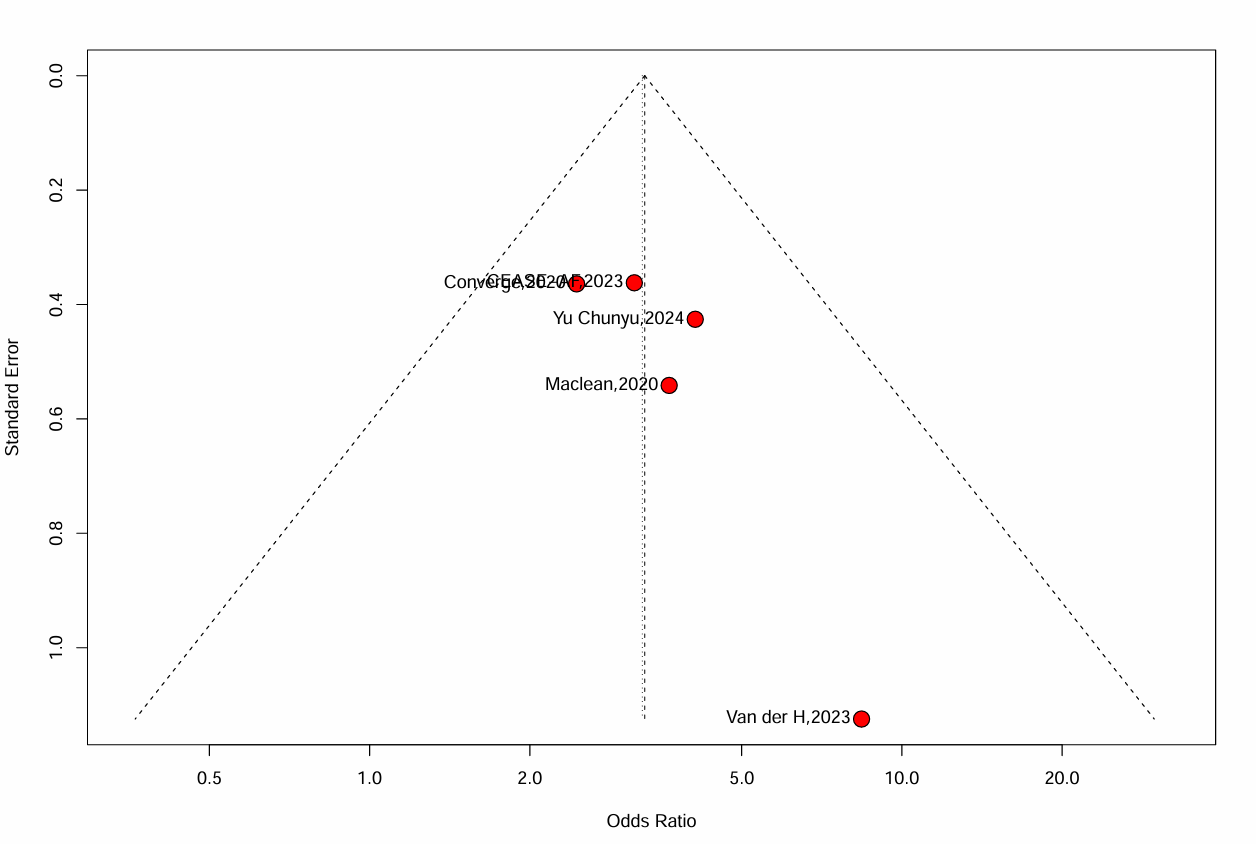


Supplemental Figure 3C. Funnel Plot for Freedom from Arrhythmia (Regardless of AADs)


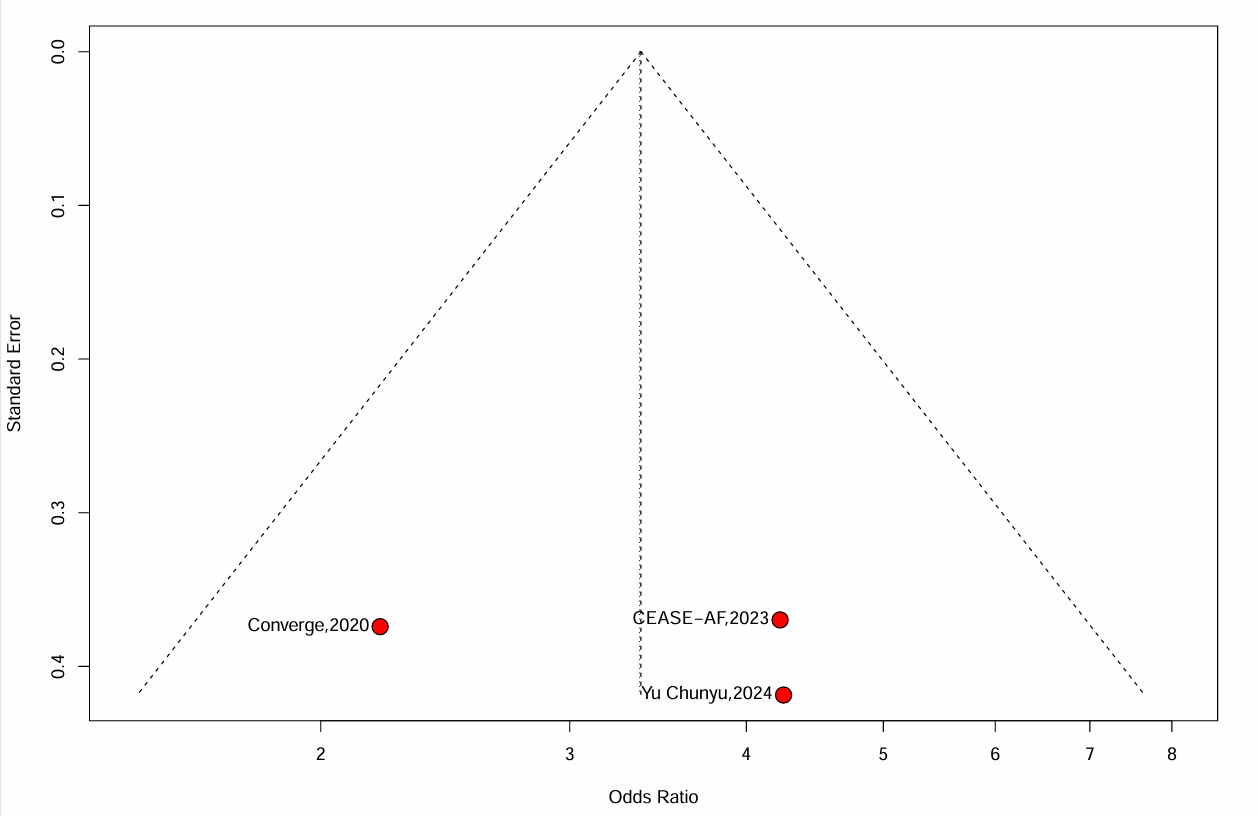


Supplemental Figure 3D. Funnel Plot for Arrhythmia Recurrence


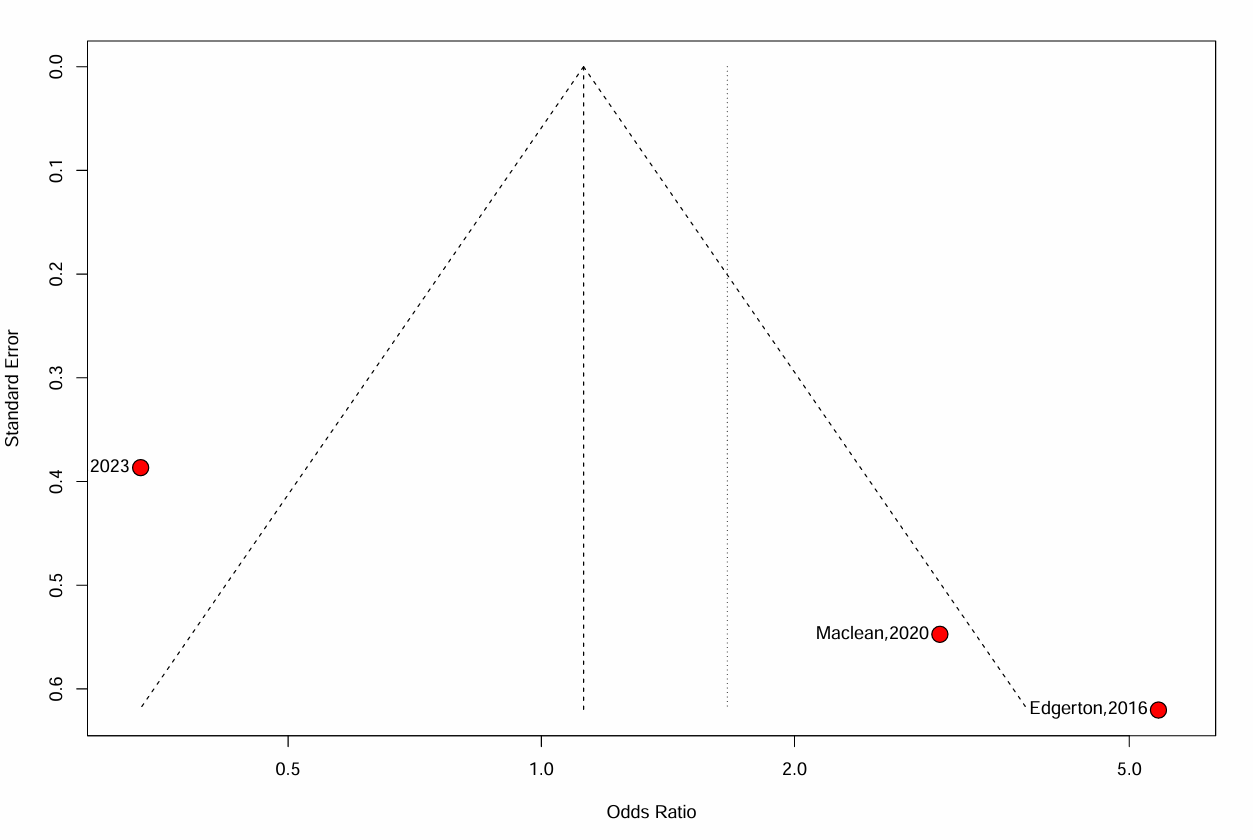


Supplemental Figure 3E. Funnel Plot for Repeat Ablation


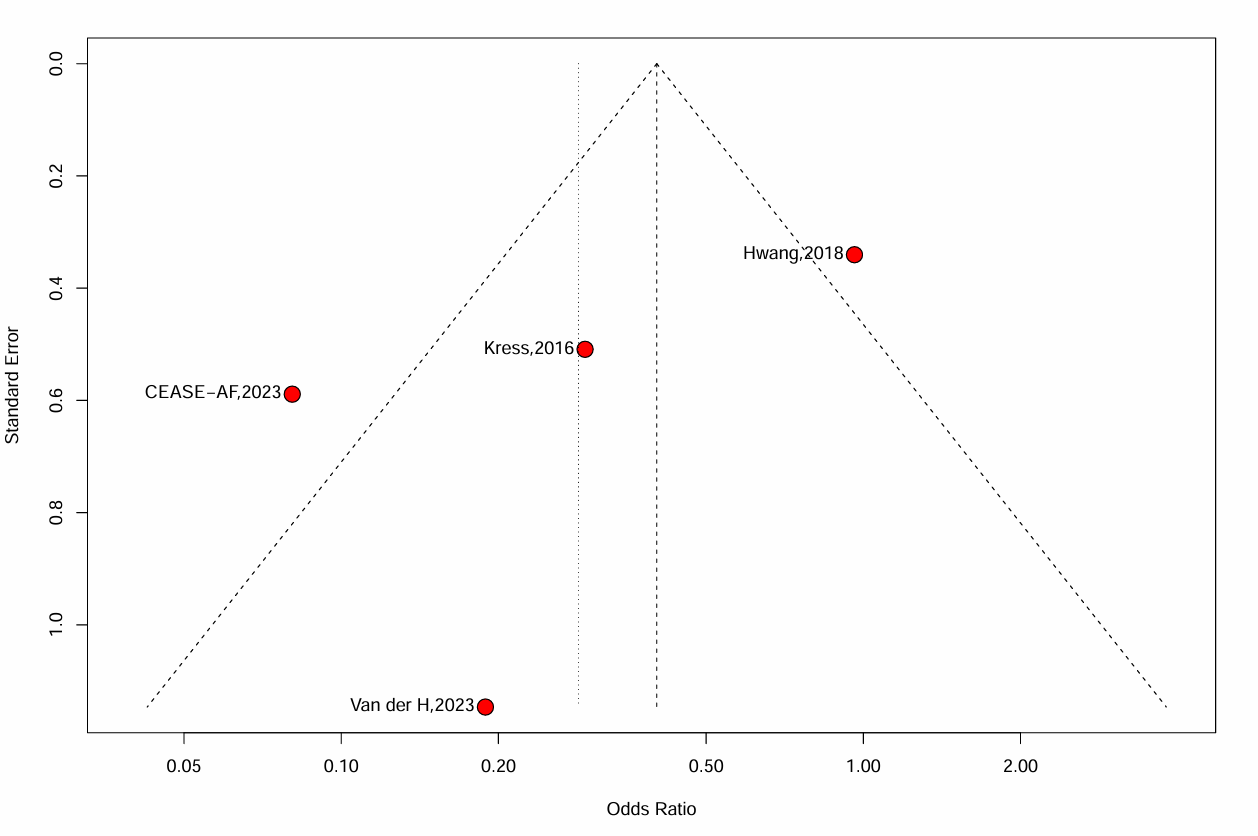


Supplemental Figure 4A. Subgroup Analysis of Type of energy for Freedom from Atrial Fibrillation


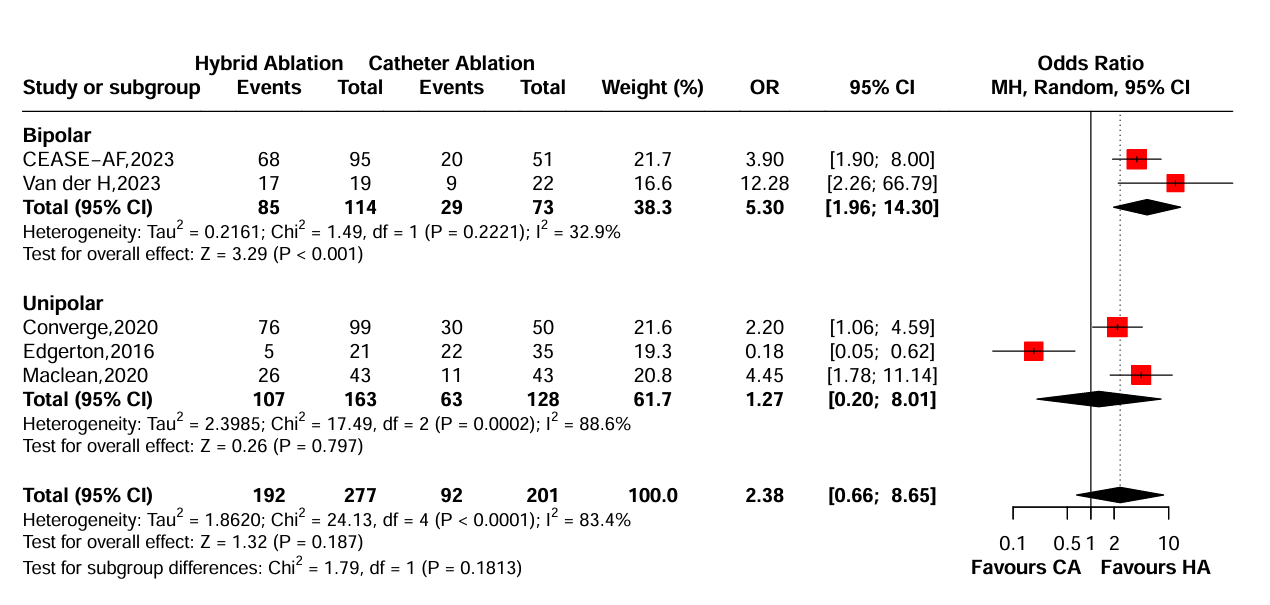


Supplemental Figure 4B. Subgroup Analysis of Type of Energy for Freedom from Anti-Arrhythmic Drug (AAD)


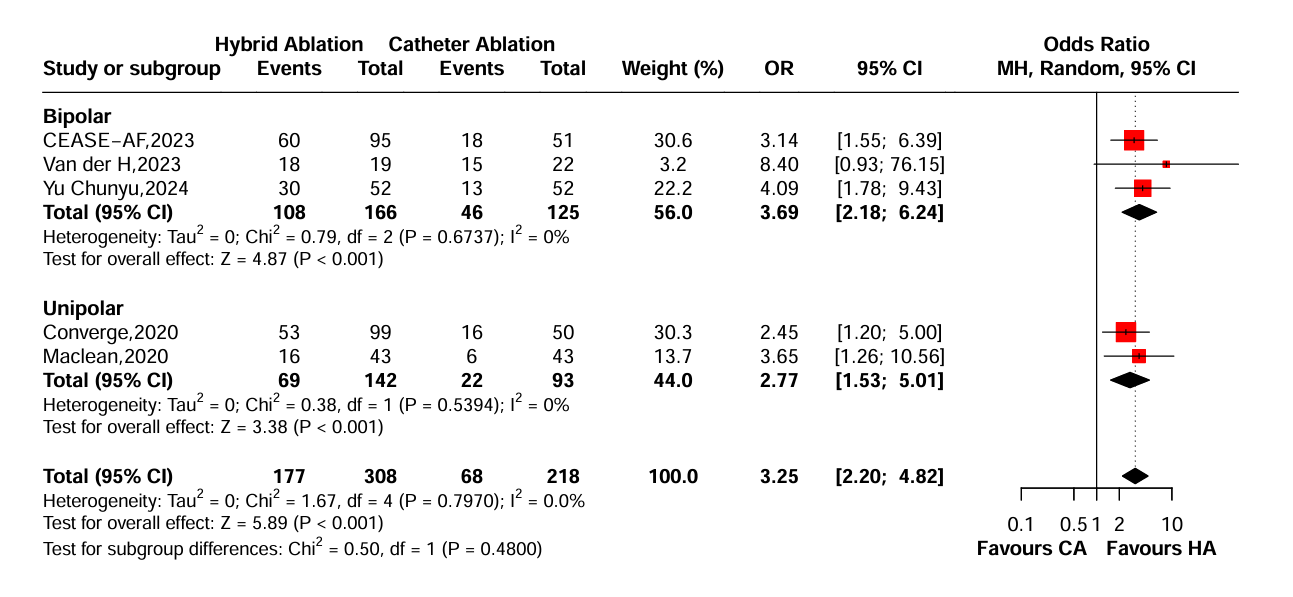


Supplemental Figure 4C. Subgroup Analysis of Type of energy for Freedom from Arrhythmia (Regardless of AADs)


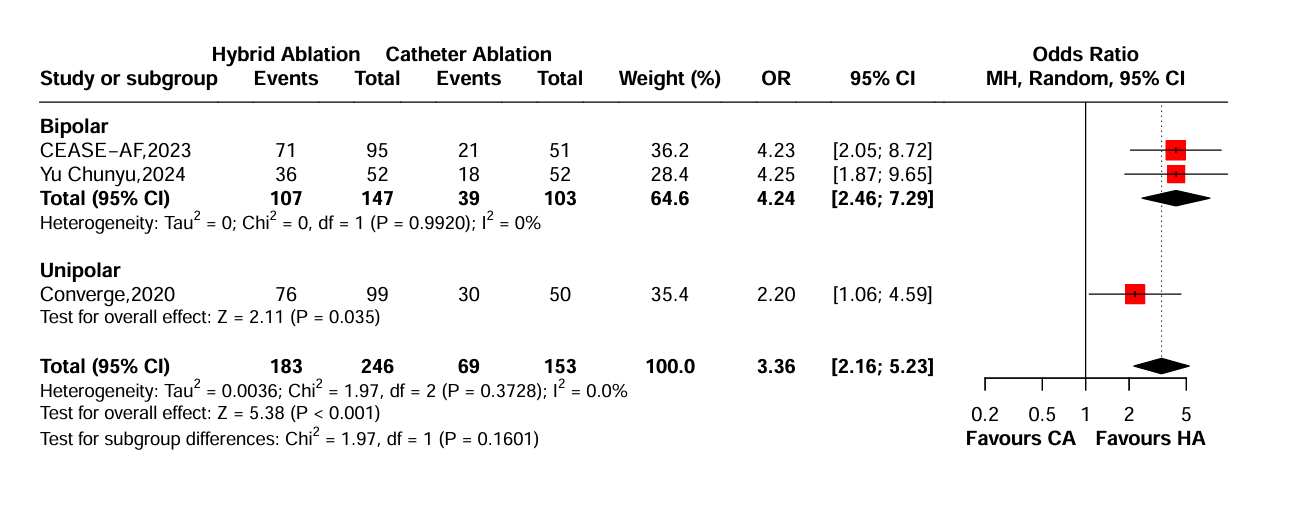


Supplemental Figure 4D. Subgroup Analysis of the Type of Energy for Arrhythmia Recurrence


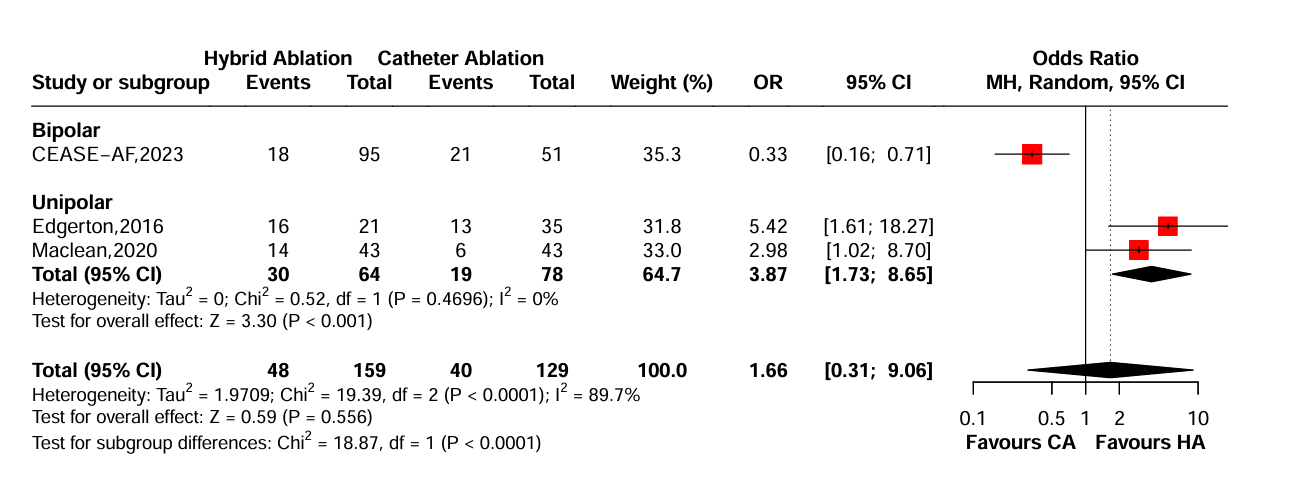


Supplemental Figure 4E. Subgroup Analysis of Type of Energy for Repeat Ablation


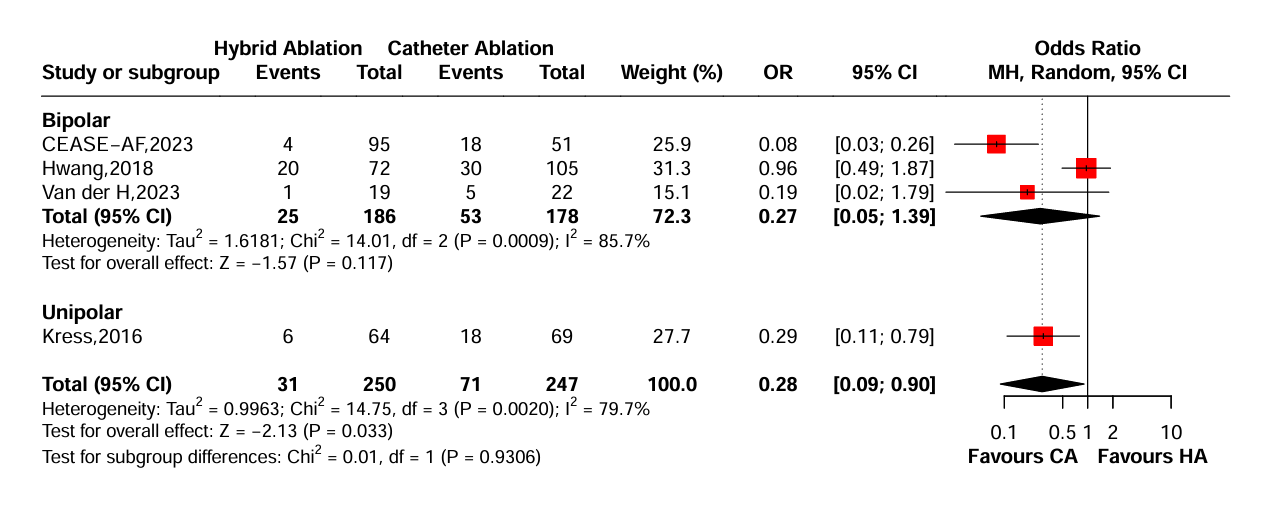


Supplemental Figure 5A. Subgroup Analysis of the Type of Study for Freedom from Atrial Fibrillation


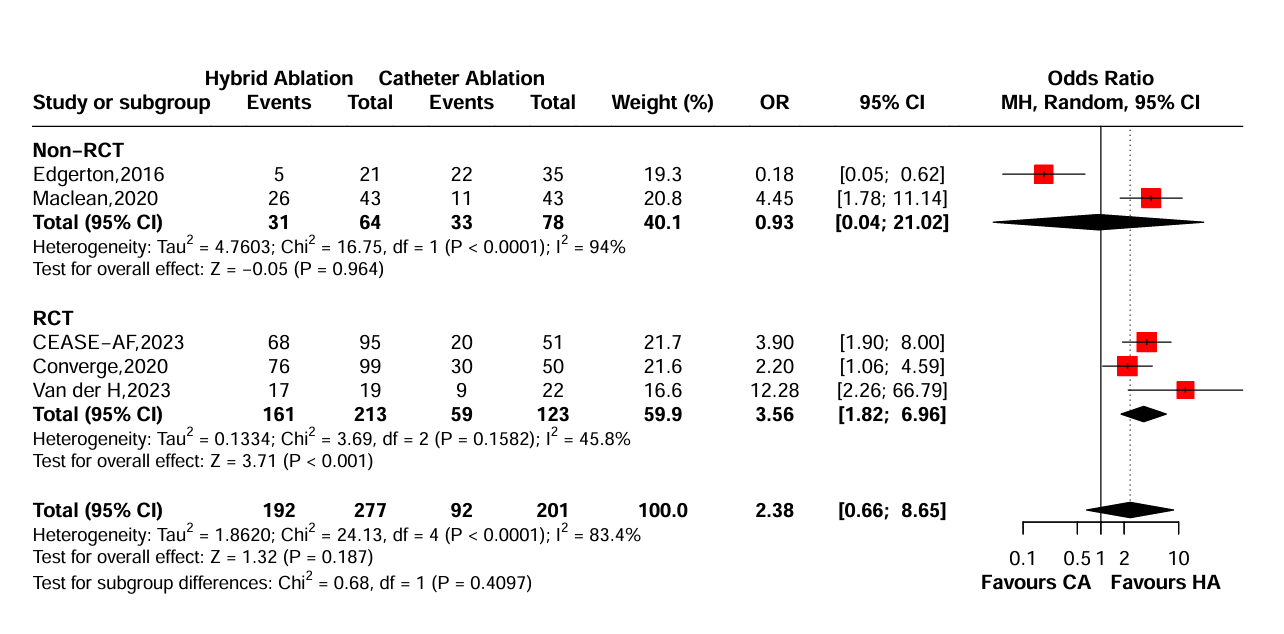


Supplemental Figure 5B. Subgroup Analysis of the Type of Study for Freedom from Anti-Arrhythmic Drug (AAD)


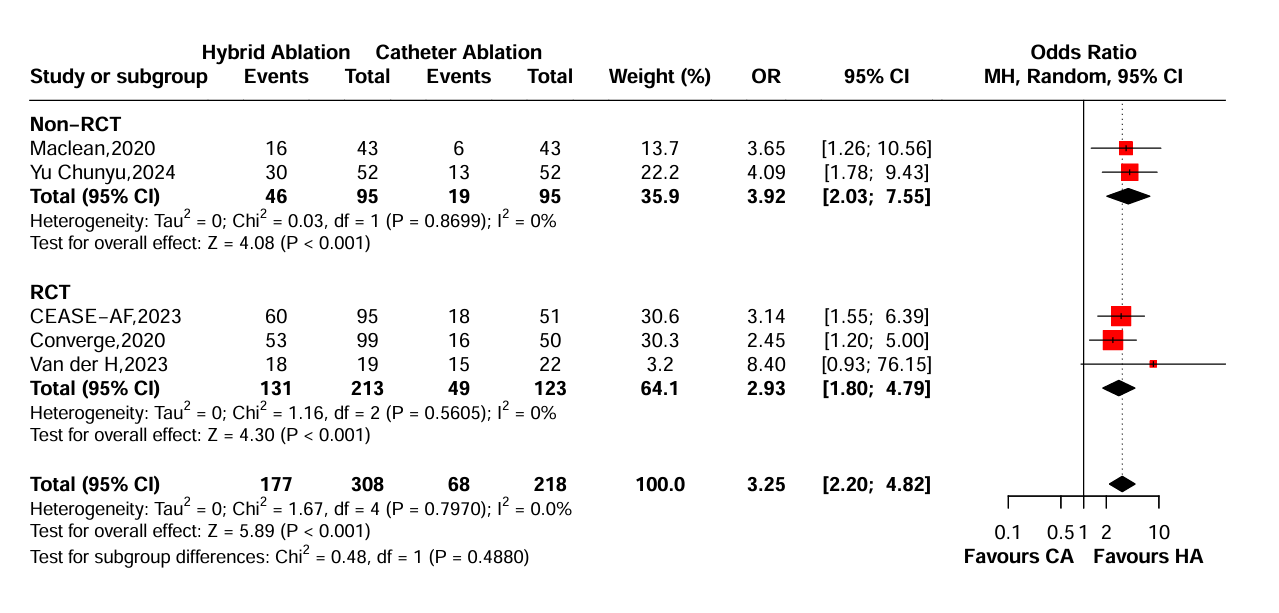


Supplemental Figure 5C. Subgroup Analysis of Type of Study for Freedom from Arrhythmia (Regardless of AADs)


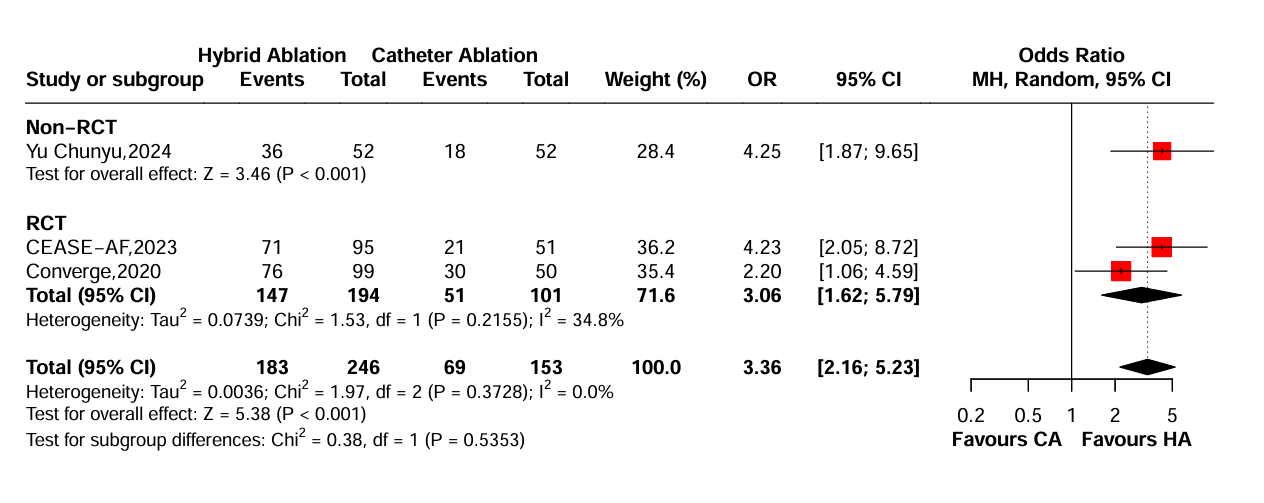


Supplemental Figure 5D. Subgroup Analysis of the Type of Study for Arrhythmia Recurrence


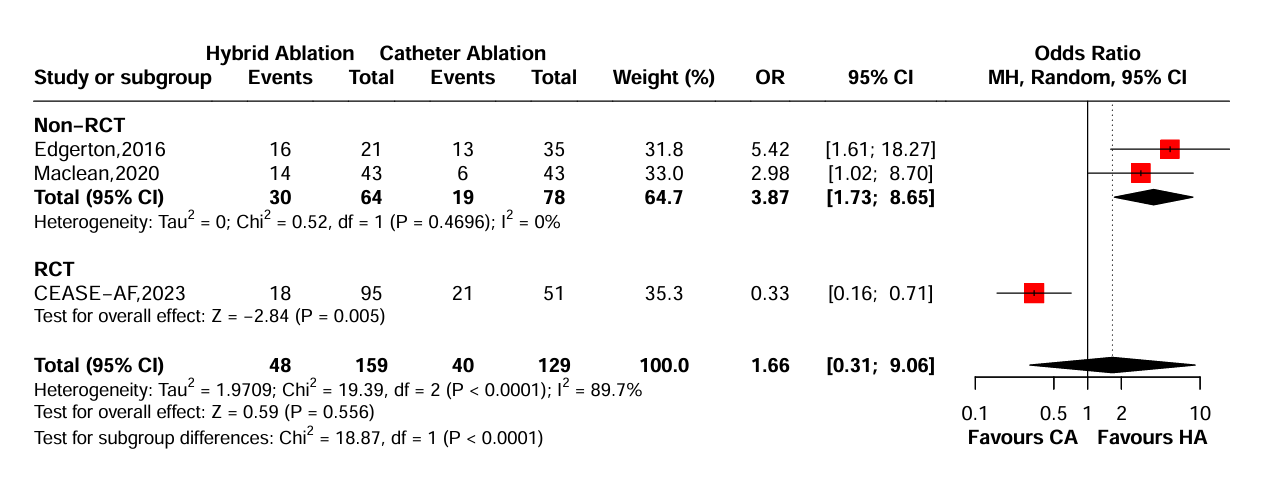


Supplemental Figure 5E. Subgroup Analysis of the Type of Study for Repeat Ablation


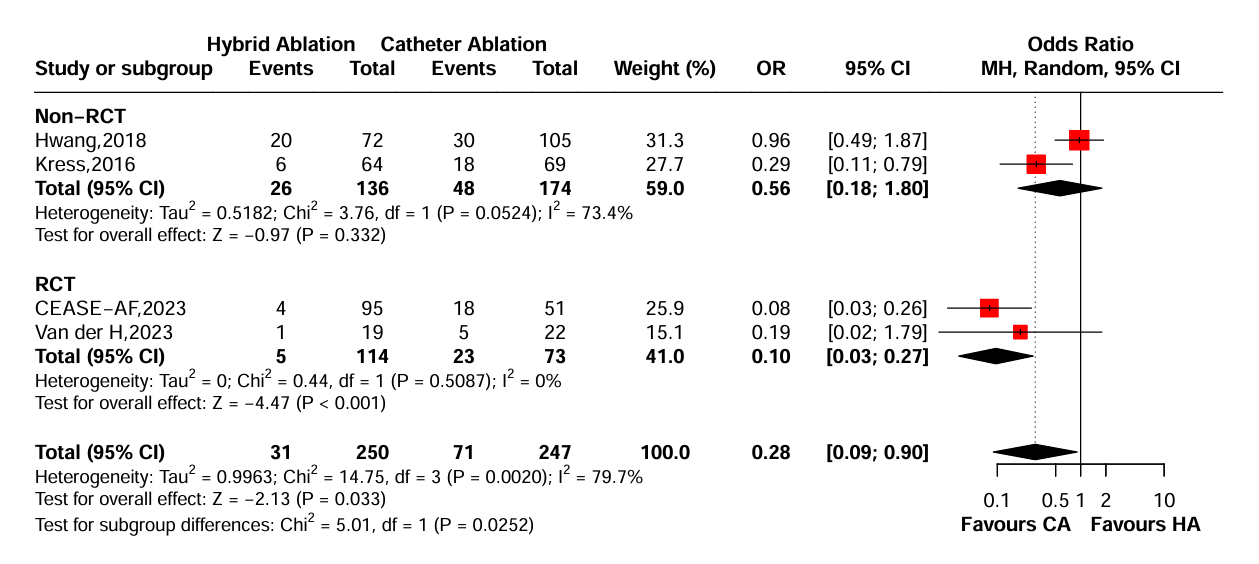


Supplemental Figure 6A. Meta regression analysis of Age for Freedom from Atrial Fibrillation

| **Moderator** | **Estimate (SE)** | **95% CI** | **p-value** | **τ²** | **I²** | **QM (df=1)** |
| --- | --- | --- | --- | --- | --- | --- |
| **Age** | –0.12 (0.30) | –0.71 to 0.46 | 0.68 | 2.55 | 90.9% | 0.17 |


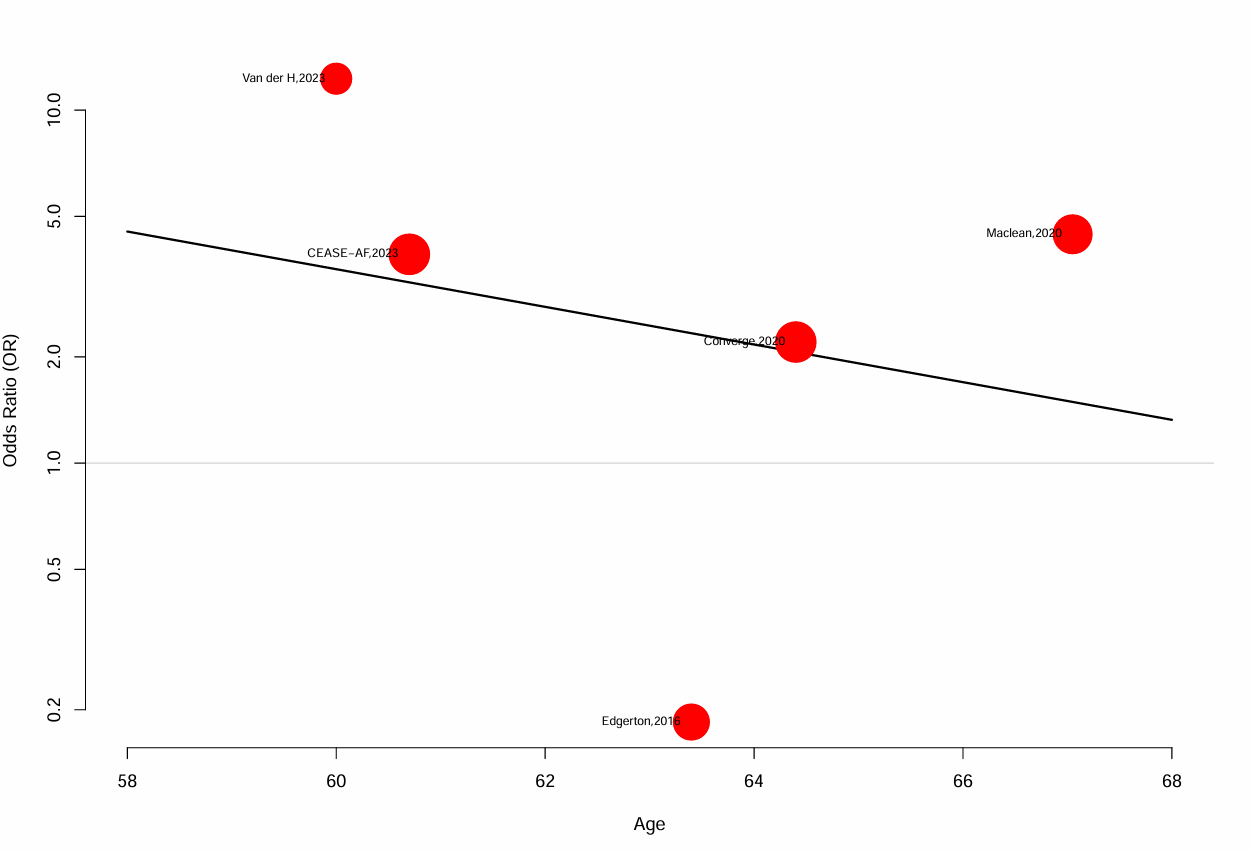


Supplemental Figure 6B. Meta regression analysis of Age for Freedom from Anti-Arrhythmic Drug (AAD)

| **Moderator** | **Estimate (SE)** | **95% CI** | **p-value** | **τ²** | **I²** | **QM (df=1)** |
| --- | --- | --- | --- | --- | --- | --- |
| **Age** | –0.04 (0.09) | –-0.21 to -0.13 | 0.64 | 0.00 | 0.0% | 0.22 |


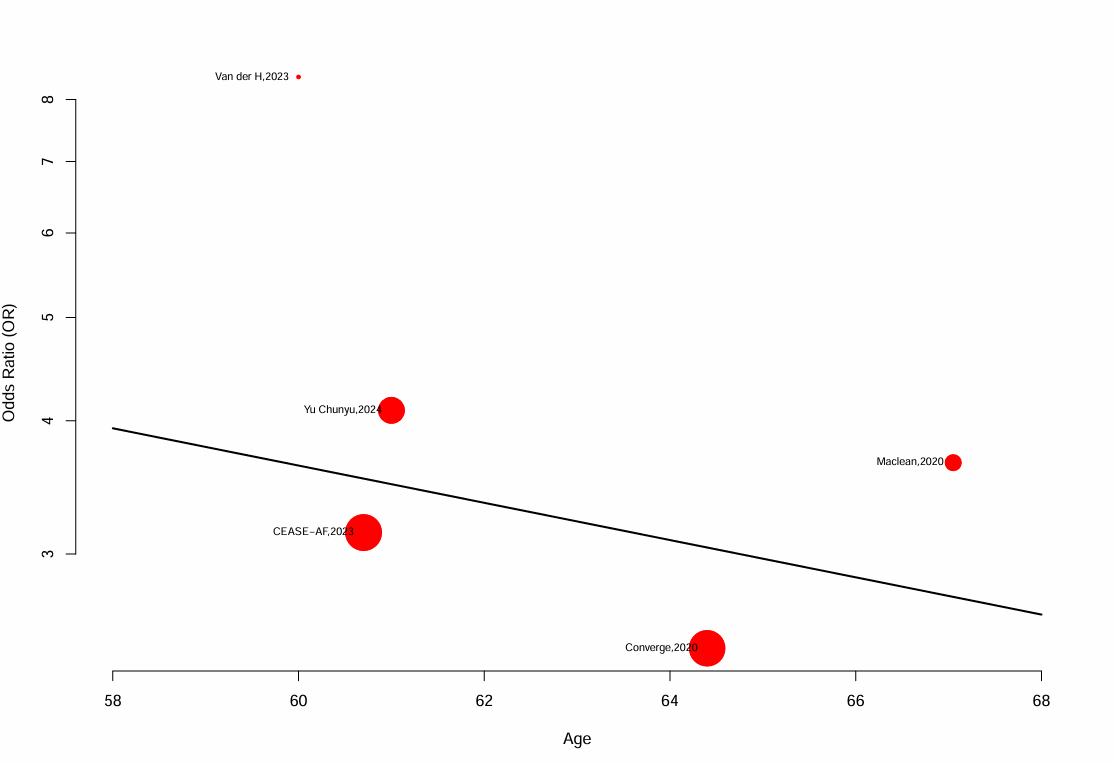


Supplemental Figure 6C. Meta regression analysis of Age for Freedom from Arrhythmia (Regardless of AADs)

| **Moderator** | **Estimate (SE)** | **95% CI** | **p-value** | **τ²** | **I²** | **QM (df=1)** |
| --- | --- | --- | --- | --- | --- | --- |
| **Age** | –0.18 (0.13) | –0.44 to 0.07 | 0.16 | 0.00 | 0.0% | 1.96 |


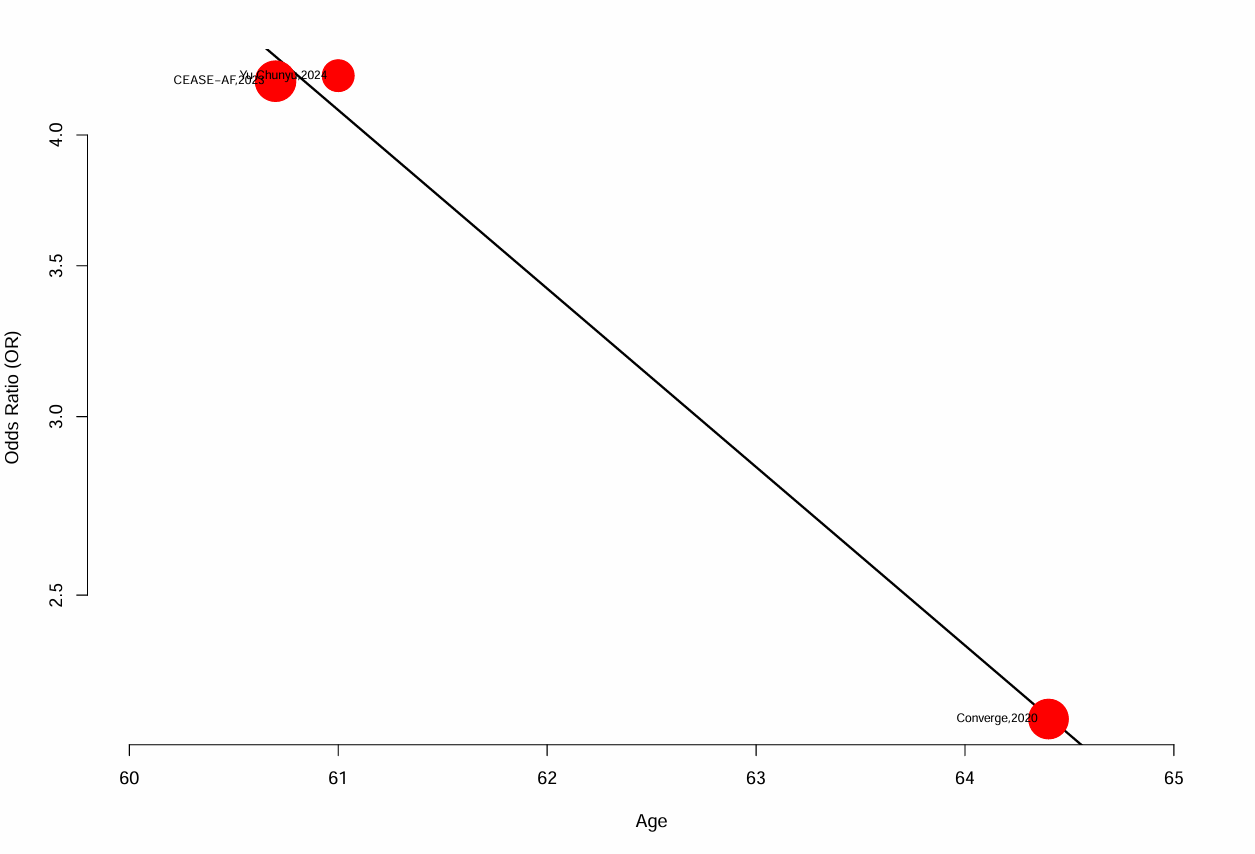


Supplemental Figure 6D. Meta regression analysis of Age for Arrhythmia Recurrence

| **Moderator** | **Estimate (SE)** | **95% CI** | **p-value** | **τ²** | **I²** | **QM (df=1)** |
| --- | --- | --- | --- | --- | --- | --- |
| **Age** | 0.32 (0.33) | –0.32 to 0.97 | 0.33 | 1.96 | 85.8% | 0.97 |


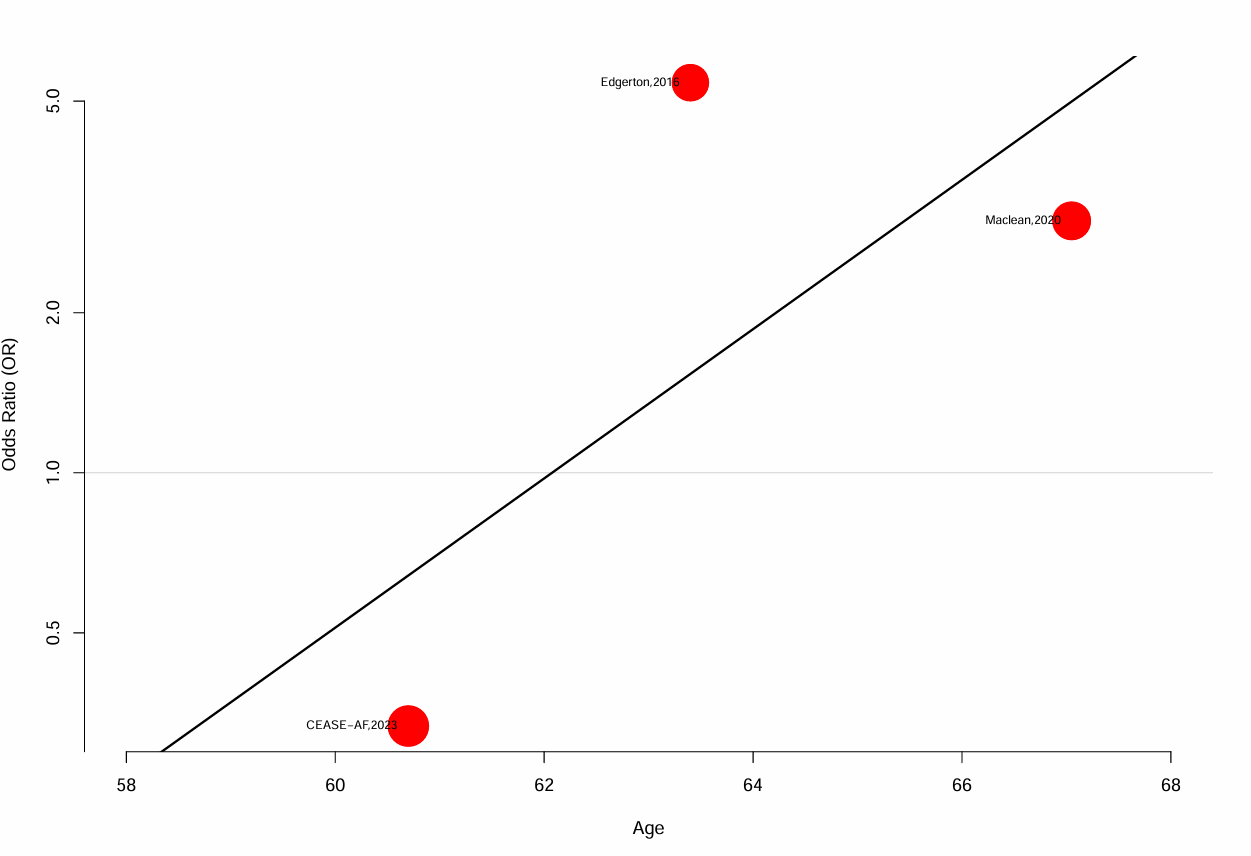


Supplemental Figure 6E. Meta regression analysis of Age for Repeat Ablation

| **Moderator** | **Estimate (SE)** | **95% CI** | **p-value** | **τ²** | **I²** | **QM (df=1)** |
| --- | --- | --- | --- | --- | --- | --- |
| **Age** | –0.21 (0.11) | –0.43 to 0.01 | 0.06 | 0.41 | 46.8% | 3.45 |


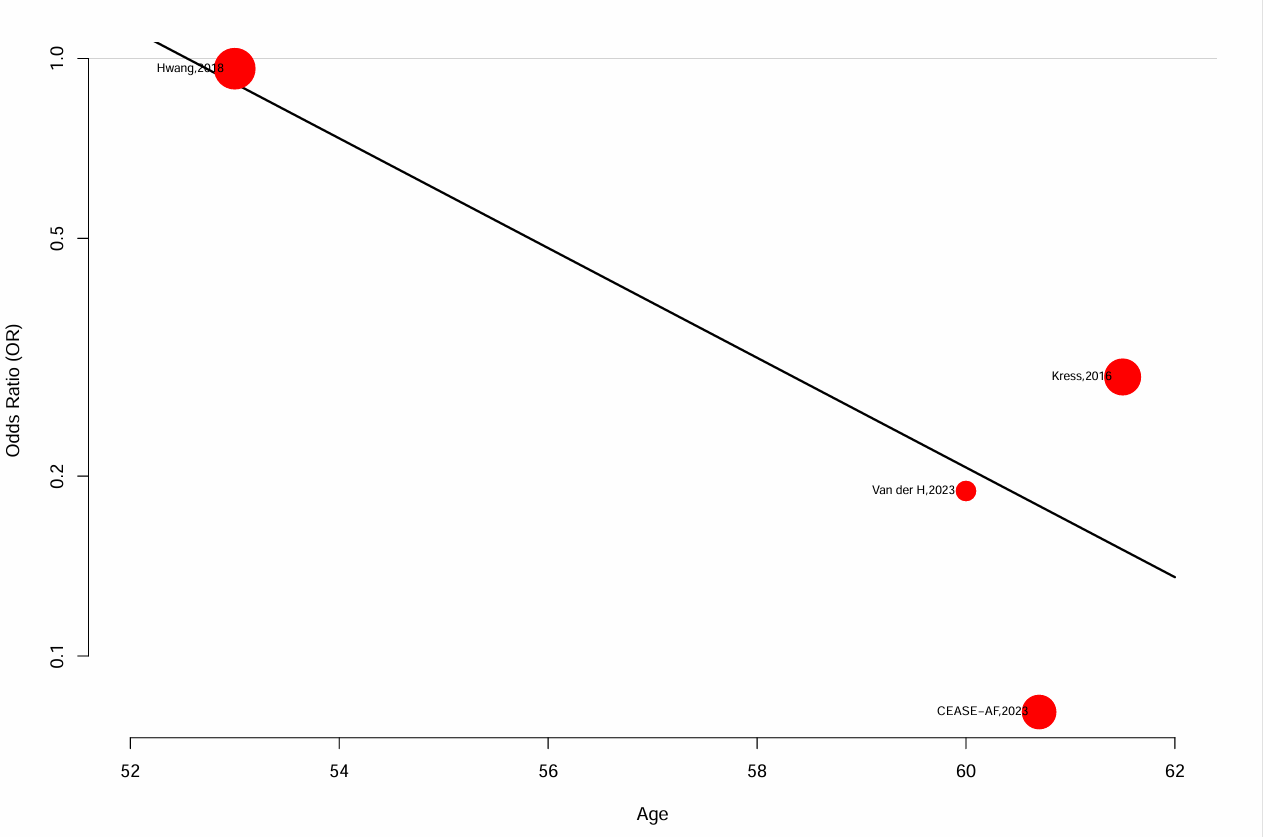


Supplemental Figure 7A. Meta regression analysis of Male sex for Freedom from Atrial Fibrillation

| **Moderator** | **Estimate (SE)** | **95% CI** | **p-value** | **τ²** | **I²** | **QM (df=1)** |
| --- | --- | --- | --- | --- | --- | --- |
| **Male** | 0.01 (0.05) | –0.09 to 0.10 | 0.90 | 2.64 | 91.9% | 0.02 |


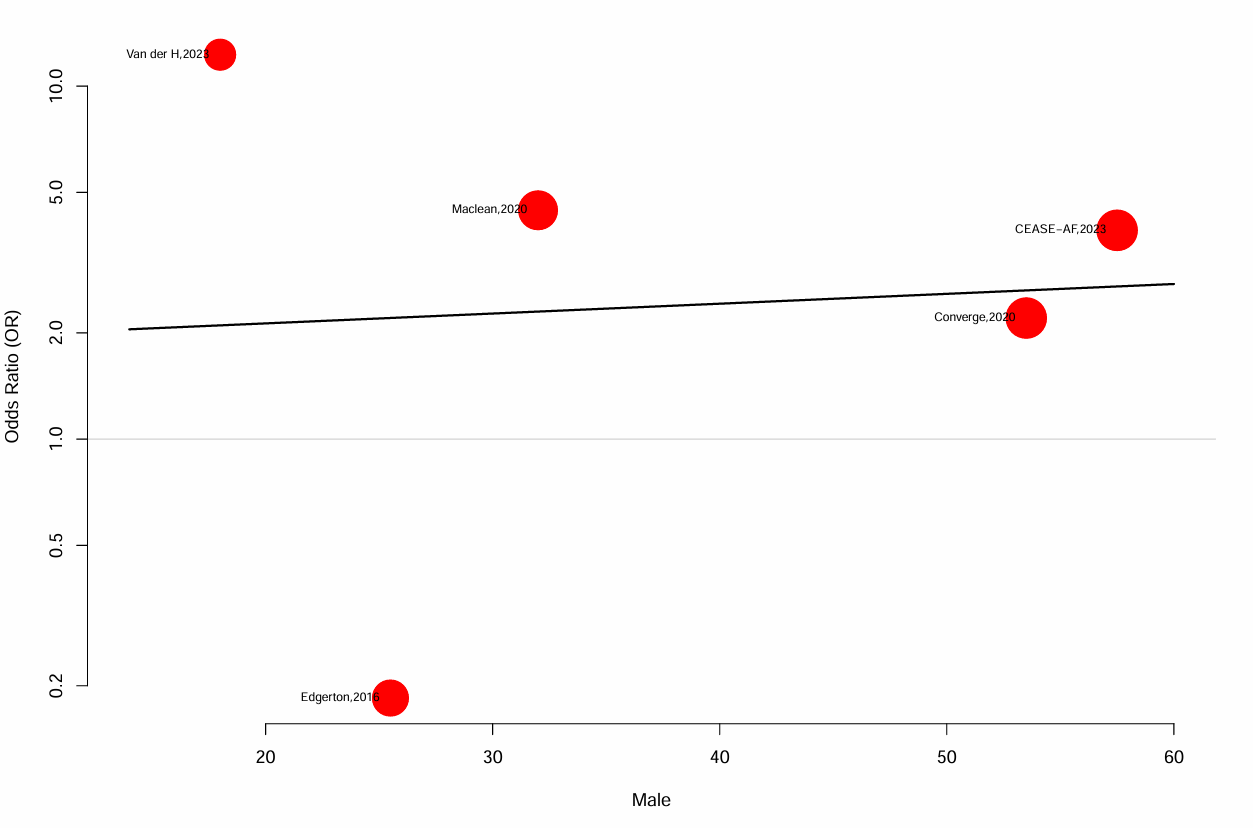


Supplemental Figure 7B. Meta regression analysis of Male sex for Freedom from Anti-Arrhythmic Drug (AAD)

| **Moderator** | **Estimate (SE)** | **95% CI** | **p-value** | **τ²** | **I²** | **QM (df=1)** |
| --- | --- | --- | --- | --- | --- | --- |
| **Male** | –0.02 (0.02) | –0.06 to 0.02 | 0.33 | 0.00 | 0.0% | 0.95 |


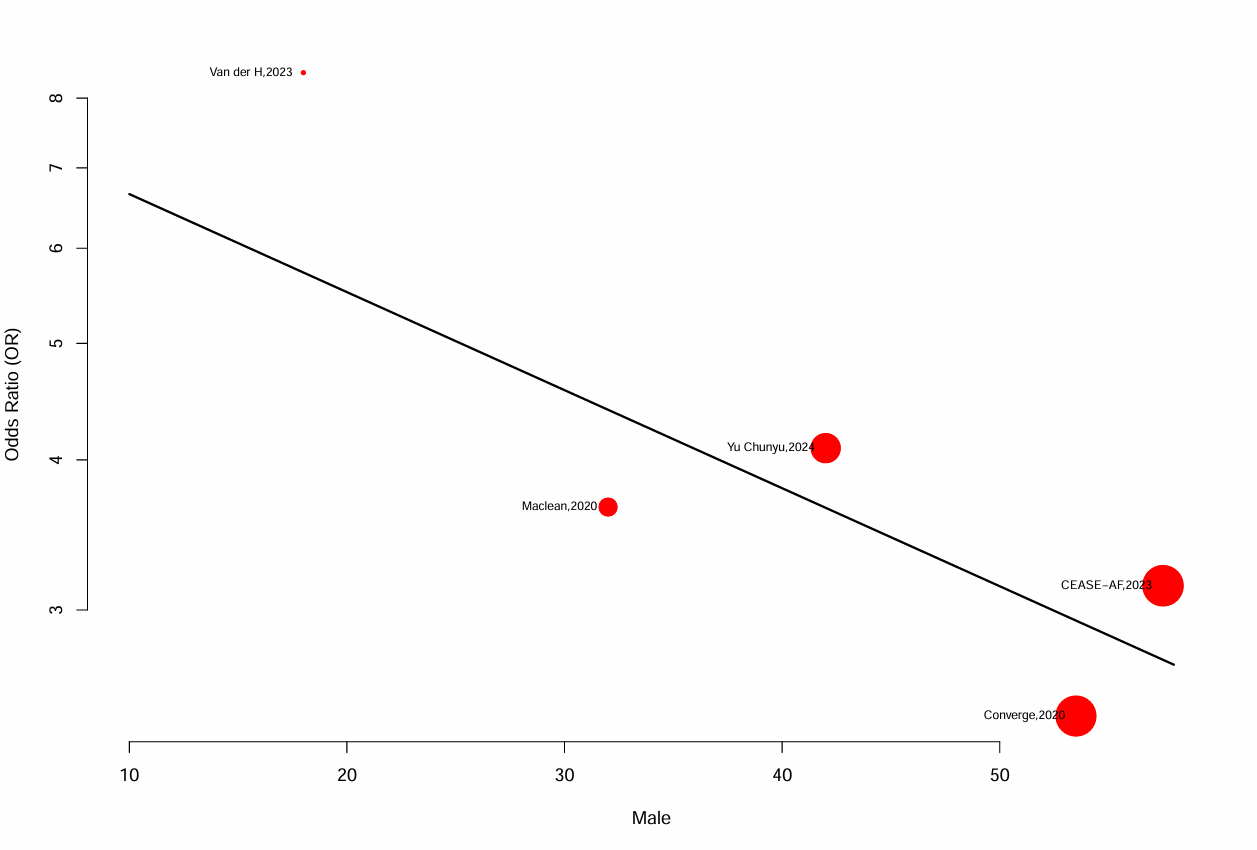


Supplemental Figure 7C. Meta regression analysis of Male sex for Freedom from Arrhythmia (Regardless of AADs)

| **Moderator** | **Estimate (SE)** | **95% CI** | **p-value** | **τ²** | **I²** | **QM (df=1)** |
| --- | --- | --- | --- | --- | --- | --- |
| **Male** | –0.01 (0.05) | –0.10 to 0.08 | 0.80 | 0.12 | 46.8% | 0.06 |


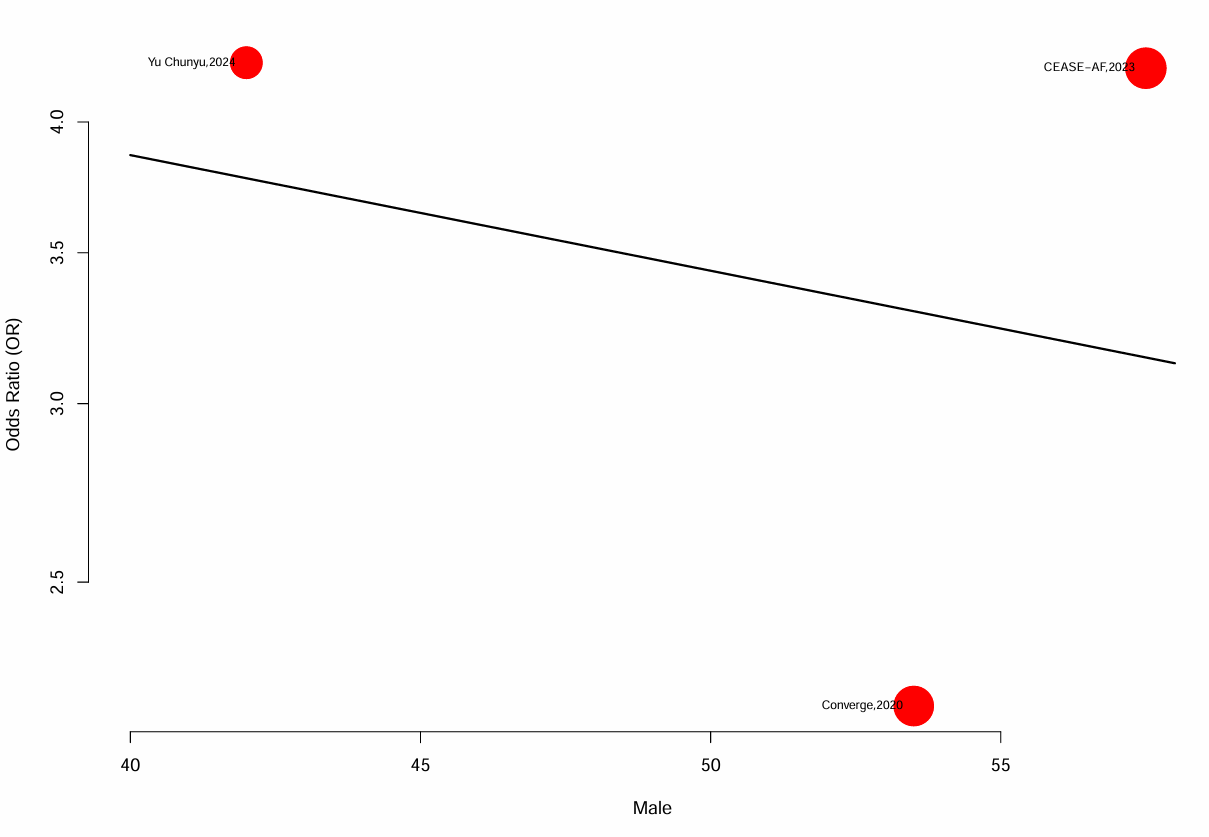


Supplemental Figure 7D. Meta regression analysis of Male sex for Arrhythmia Recurrence

| **Moderator** | **Estimate (SE)** | **95% CI** | **p-value** | **τ²** | **I²** | **QM (df=1)** |
| --- | --- | --- | --- | --- | --- | --- |
| **Male** | –0.09 (0.02) | –-0.13 to –0.05 | <0.001 | 0.00 | 0.0% | 19.39 |


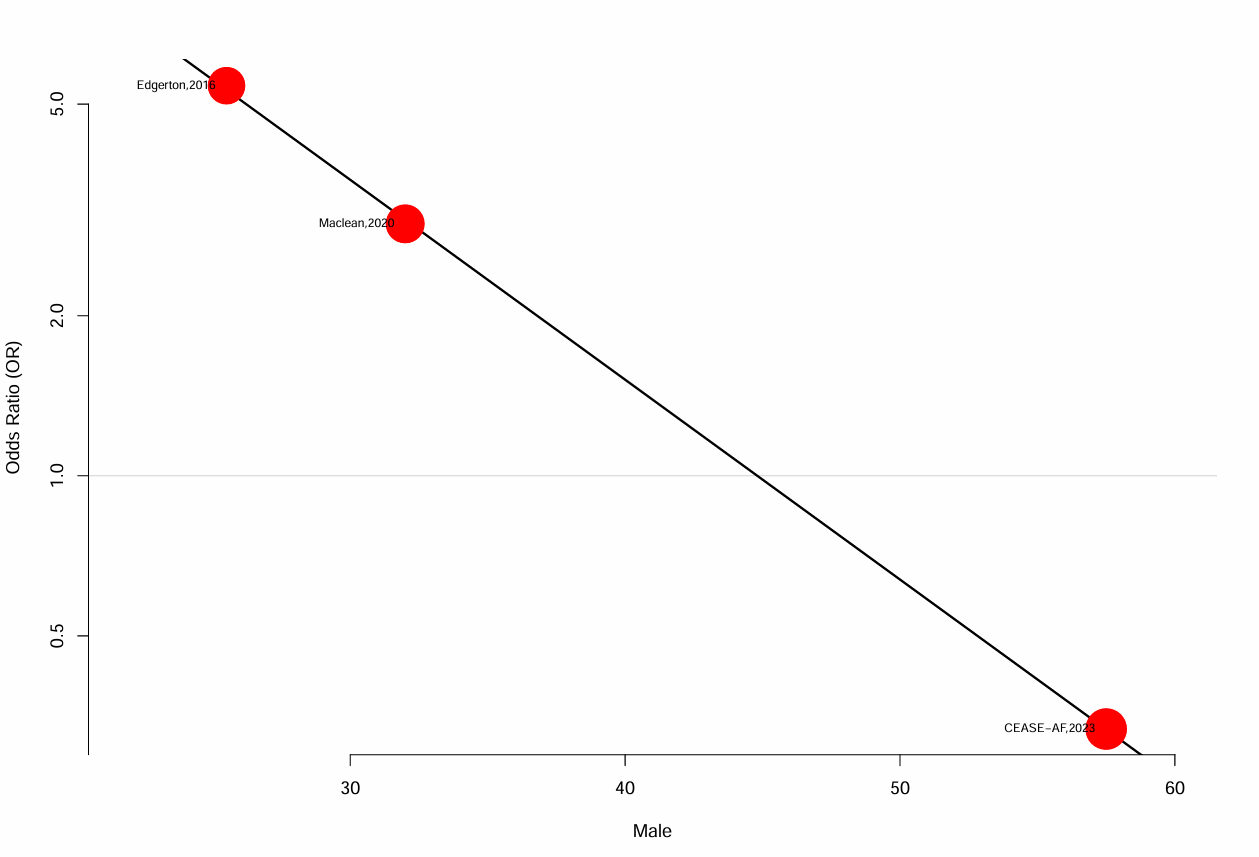


Supplemental Figure 7E. Meta regression analysis of Male sex for Repeat Ablation

| **Moderator** | **Estimate (SE)** | **95% CI** | **p-value** | **τ²** | **I²** | **QM (df=1)** |
| --- | --- | --- | --- | --- | --- | --- |
| **Male** | 0.03 (0.03) | –0.03 to 0.09 | 0.32 | 0.82 | 71.5% | 0.98 |


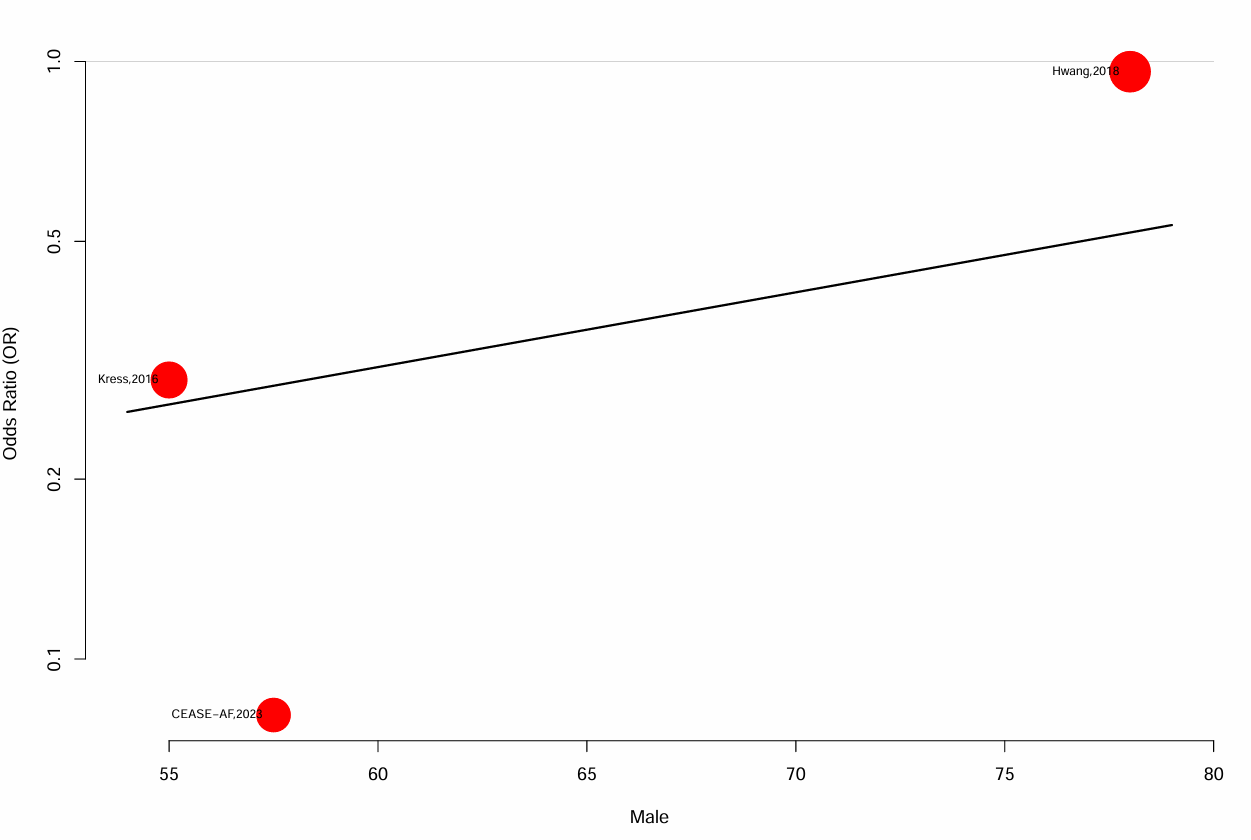


Supplemental Figure 8A. Meta regression analysis of LVEF for Freedom from Atrial Fibrillation

| **Moderator** | **Estimate (SE)** | **95% CI** | **p-value** | **τ²** | **I²** | **QM (df=1)** |
| --- | --- | --- | --- | --- | --- | --- |
| **LVEF** | 0.14 (0.25) | –-0.34 to 0.62 | 0.58 | 2.45 | 90.7% | 0.31 |


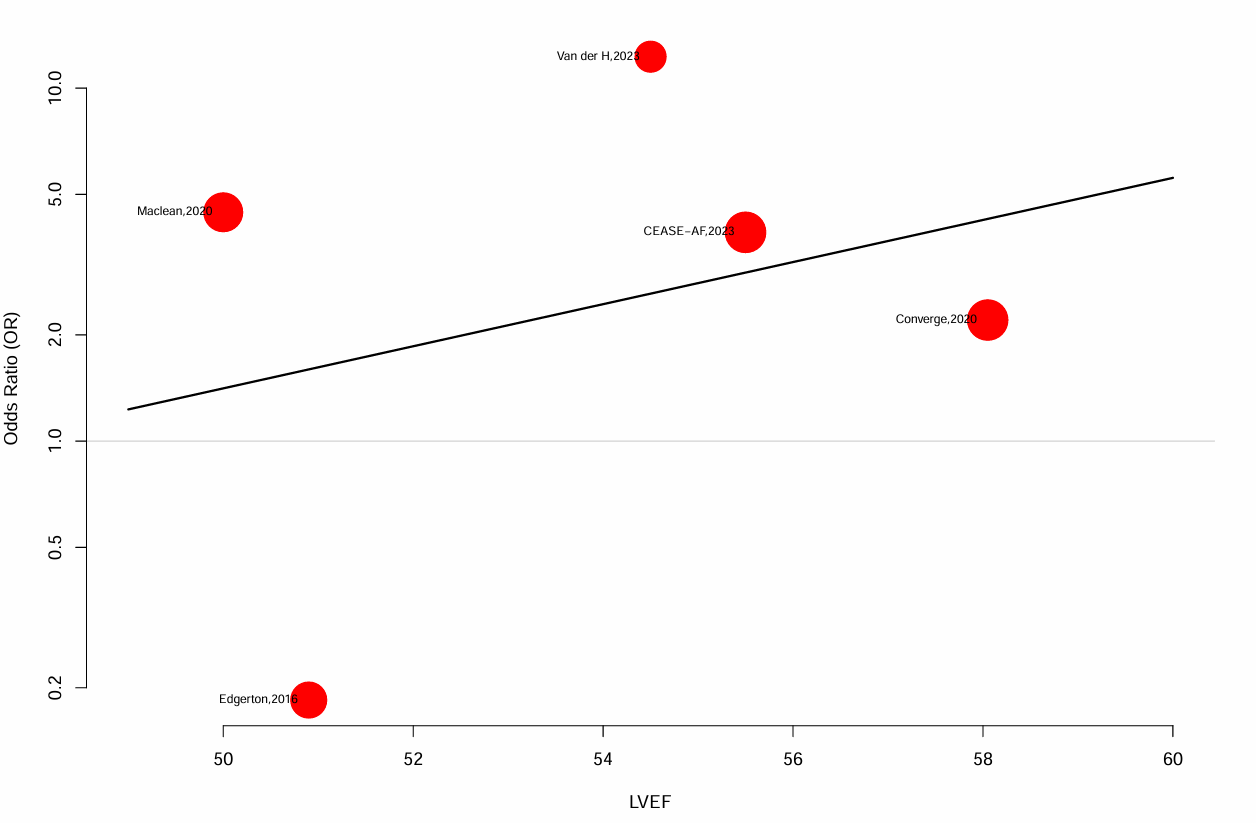


Supplemental Figure 8B. Meta regression analysis of LVEF for Freedom from Anti-Arrhythmic Drug (AAD)

| **Moderator** | **Estimate (SE)** | **95% CI** | **p-value** | **τ²** | **I²** | **QM (df=1)** |
| --- | --- | --- | --- | --- | --- | --- |
| **LVEF** | −0.0065 (0.0606) | −0.1253 to 0.1123 | 0.9146 | 0 | 0.0% | 0.0115 |


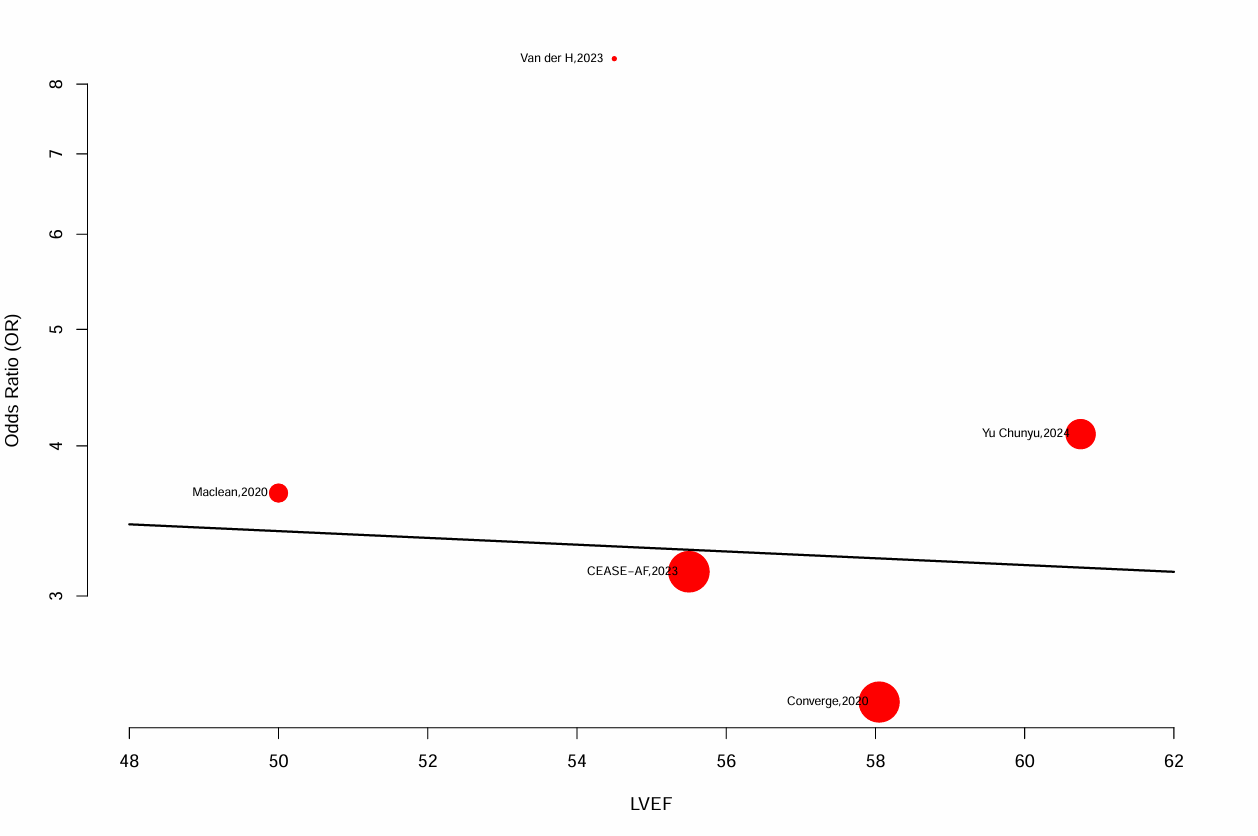


Supplemental Figure 8C. Meta regression analysis of LVEF for Freedom from Arrhythmia (Regardless of AADs)

| **Moderator** | **Estimate (SE)** | **95% CI** | **p-value** | **τ²** | **I²** | **QM (df=1)** |
| --- | --- | --- | --- | --- | --- | --- |
| **LVEF** | −0.0021 (0.1466) | −0.2894 to 0.2853 | 0.9887 | 0.1403 | 49.2% | 0.0002 |


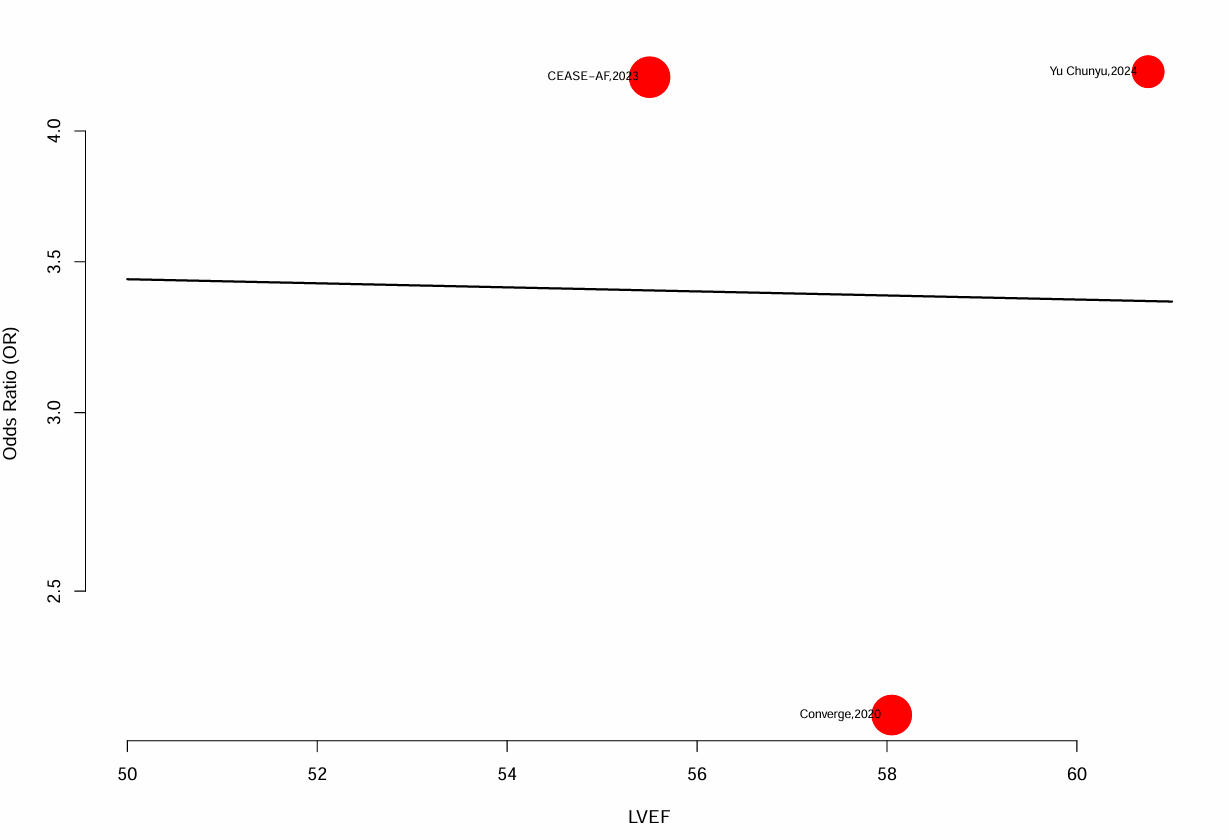


Supplemental Figure 8D. Meta regression analysis of LVEF for Arrhythmia Recurrence

| **Moderator** | **Estimate (SE)** | **95% CI** | **p-value** | **τ²** | **I²** | **QM (df=1)** |
| --- | --- | --- | --- | --- | --- | --- |
| **LVEF** | −0.4642 (0.1501) | −0.7584 to −0.1699 | 0.0020 | 0.1831 | 34.56% | 9.5580 |


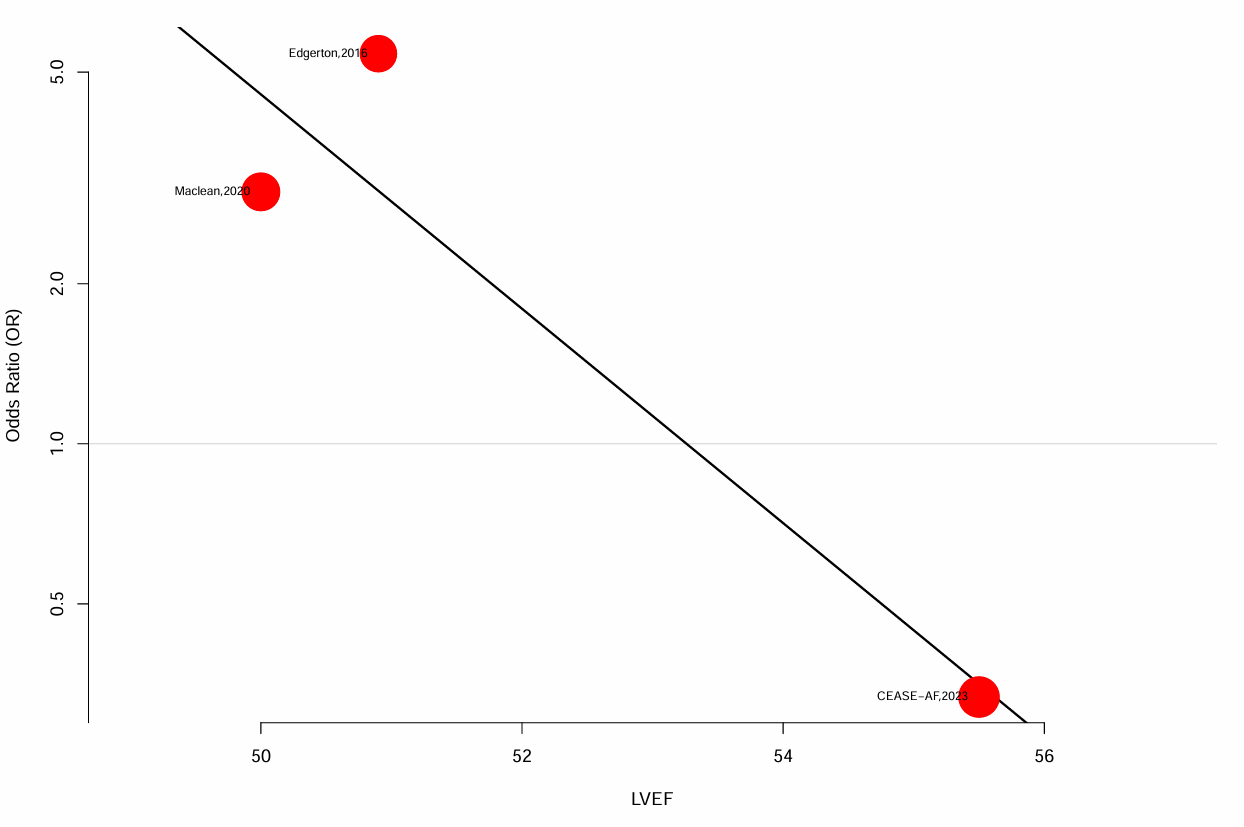


Supplemental Figure 8E. Meta regression analysis of LVEF for Repeat Ablation

| **Moderator** | **Estimate (SE)** | **95% CI** | **p-value** | **τ²** | **I²** | **QM (df=1)** |
| --- | --- | --- | --- | --- | --- | --- |
| **LVEF** | 0.2487 (0.1995) | −0.1423 to 0.6396 | 0.2126 | 0.7725 | 62.39% | 1.5537 |


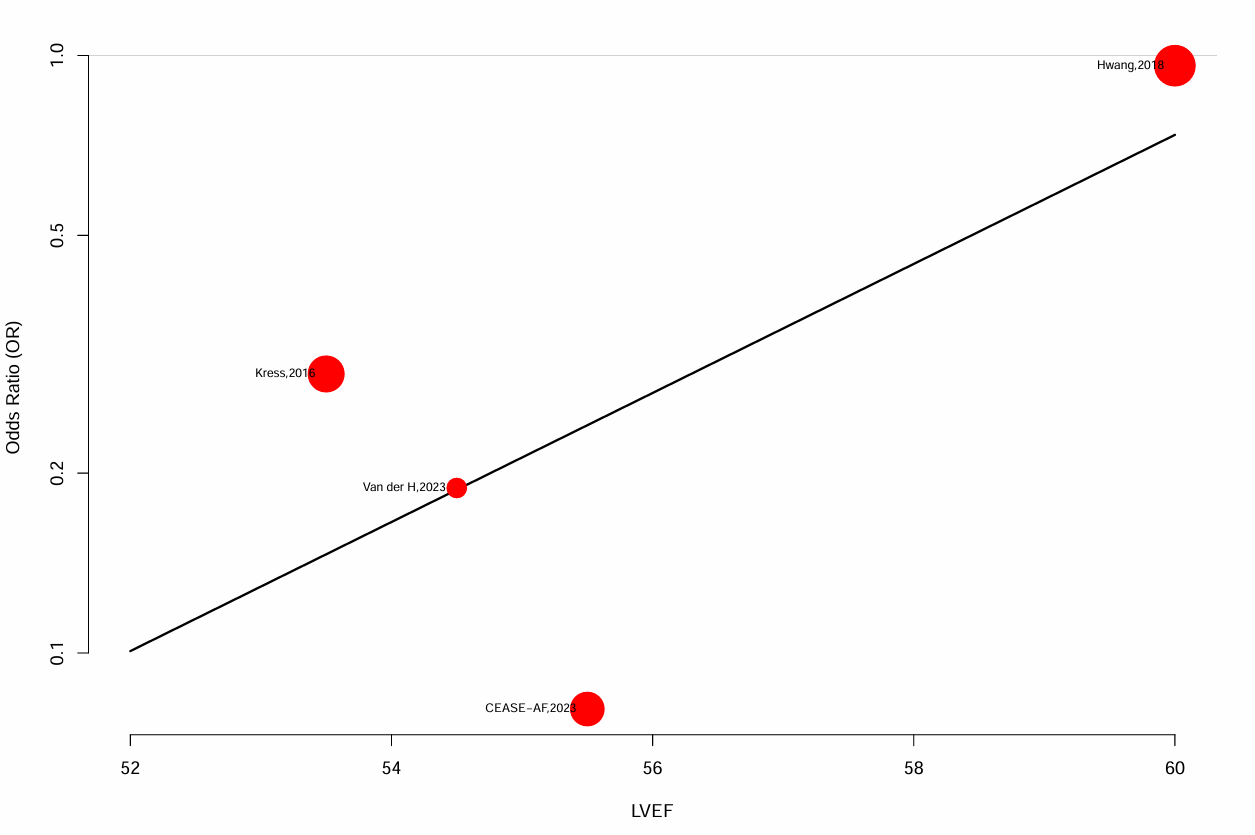


Supplemental Figure 9A. Meta regression analysis of Duration of AF for Freedom from Atrial Fibrillation

| **Moderator** | **Estimate (SE)** | **95% CI** | **p-value** | **τ²** | **I²** | **QM (df=1)** |
| --- | --- | --- | --- | --- | --- | --- |
| **Duration** | −0.8813 (0.1902) | −1.2542 to −0.5085 | < .0001 | 0.0033 | 1.45% | 21.4659 |


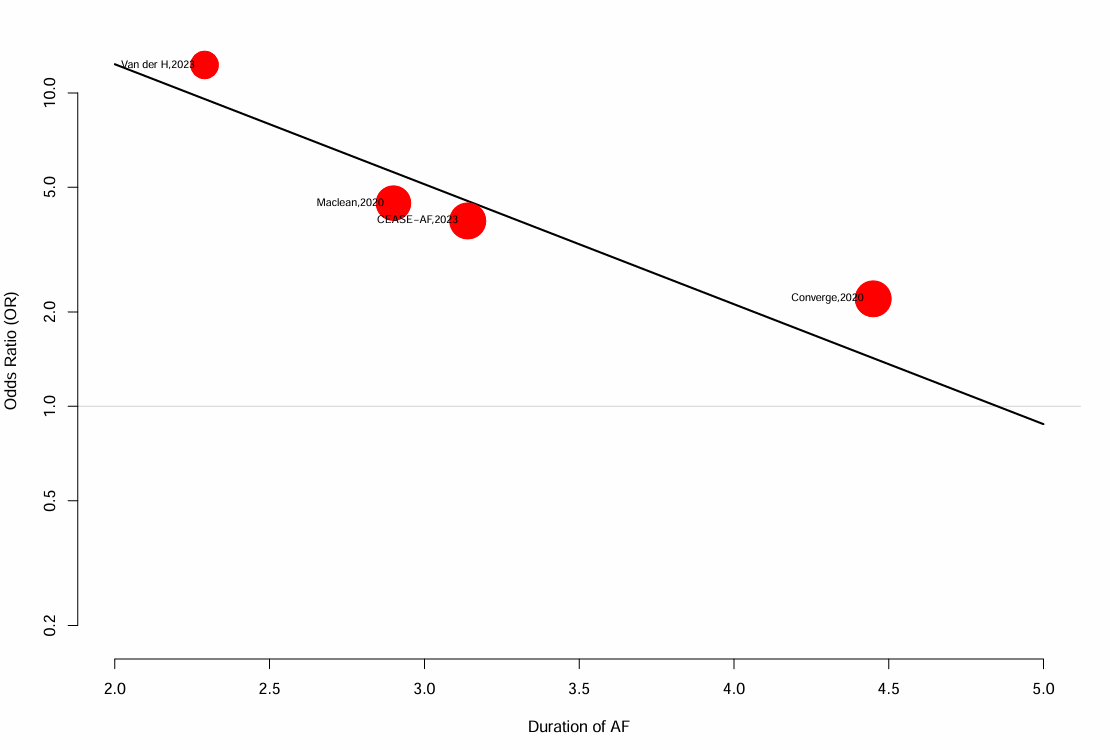


Supplemental Figure 9B. Meta regression analysis of Duration of AF for Freedom from Anti-Arrhythmic Drug (AAD)

| **Moderator** | **Estimate (SE)** | **95% CI** | **p-value** | **τ²** | **I²** | **QM (df=1)** |
| --- | --- | --- | --- | --- | --- | --- |
| **Duration** | −0.2714 (0.3129) | −0.8847 to 0.3418 | 0.3857 | 0 | 0.00% | 0.7525 |


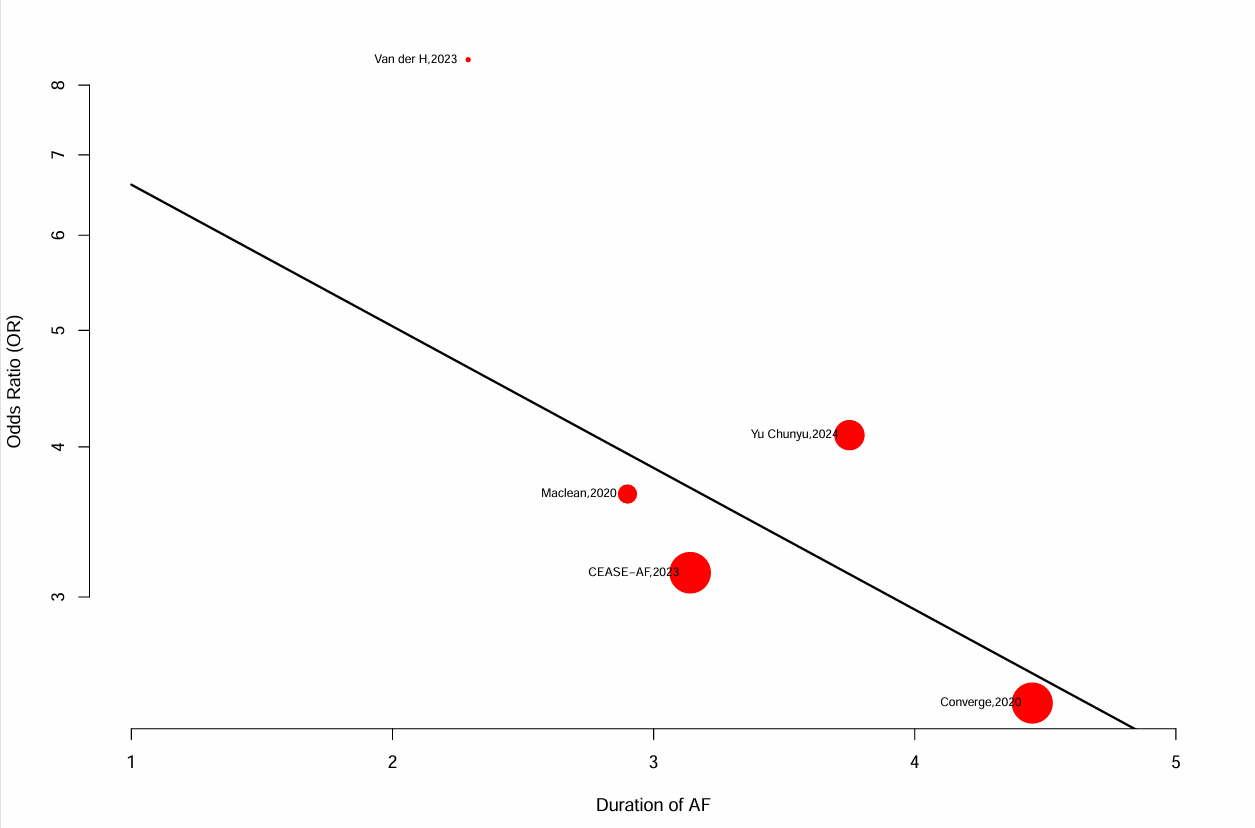


Supplemental Figure 9C. Meta regression analysis of Duration of AF for Freedom from Arrhythmia (Regardless of AADs)

| **Moderator** | **Estimate (SE)** | **95% CI** | **p-value** | **τ²** | **I²** | **QM (df=1)** |
| --- | --- | --- | --- | --- | --- | --- |
| **Duration** | −0.5049 (0.4013) | −1.2916 to 0.2817 | 0.2083 | 0 | 0.00% | 1.5829 |


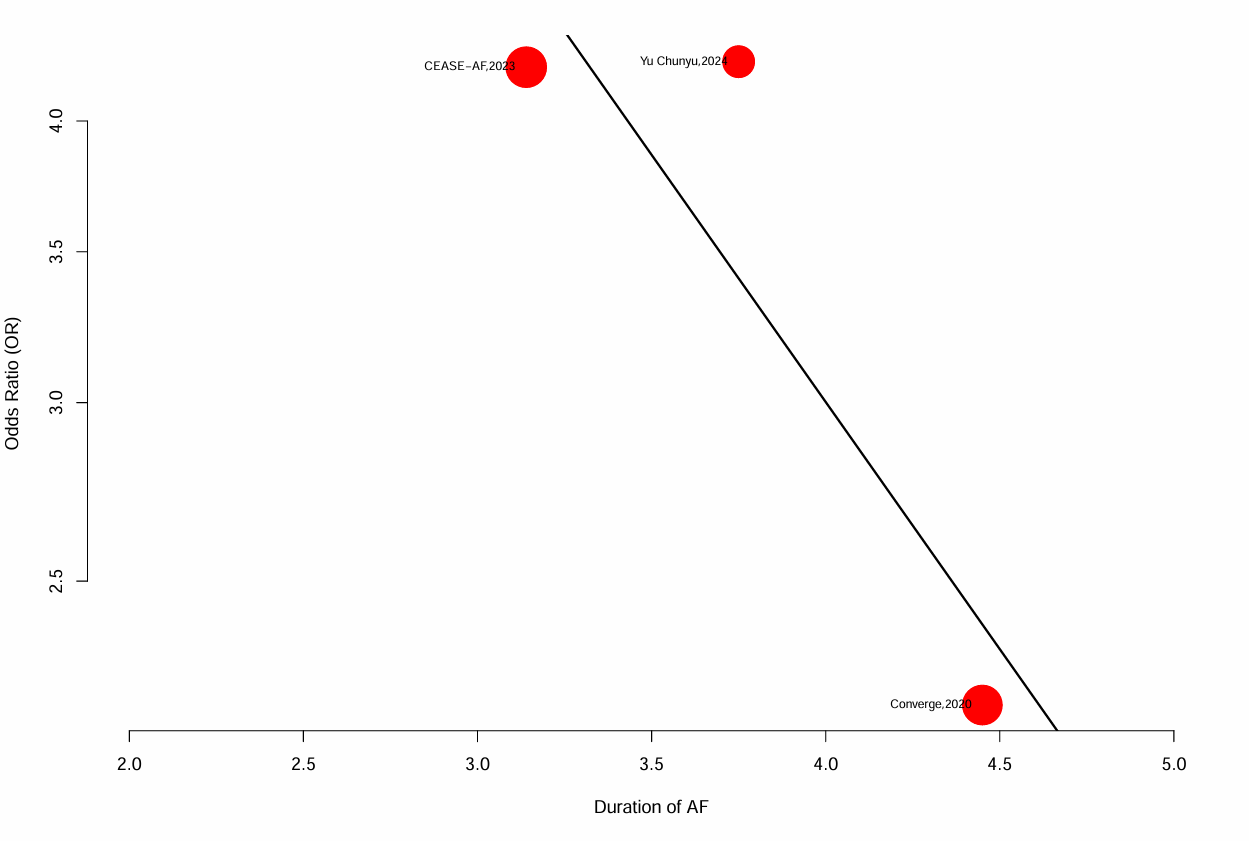


Supplemental Figure 9D. Meta regression analysis of Duration of AF for Arrhythmia Recurrence

| **Moderator** | **Estimate (SE)** | **95% CI** | **p-value** | **τ²** | **I²** | **QM (df=1)** |
| --- | --- | --- | --- | --- | --- | --- |
| **Duration** | 0.4996 (0.6415) | −0.7577 to 1.7568 | 0.4361 | 2.4497 | 91.78% | 0.6065 |


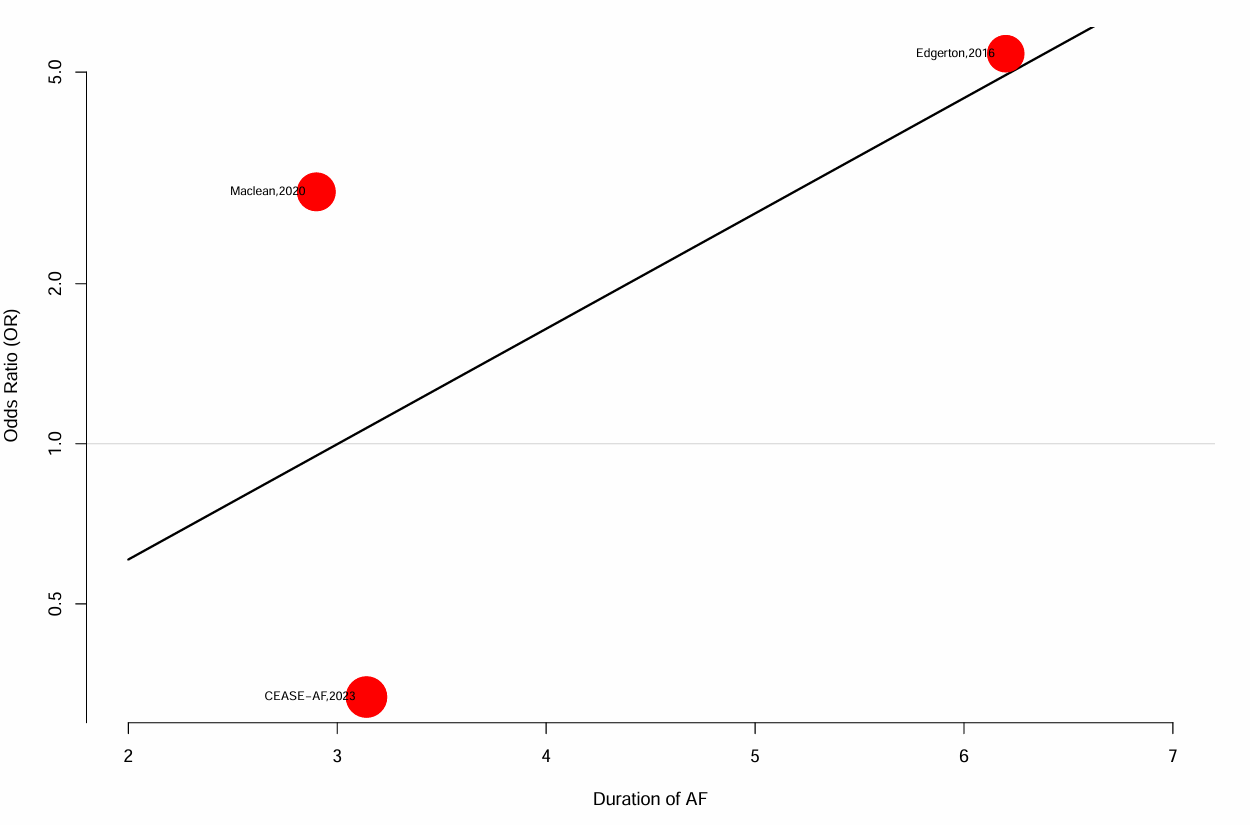


Supplemental Figure 10A. Forest Plot for Cardiac Tamponade


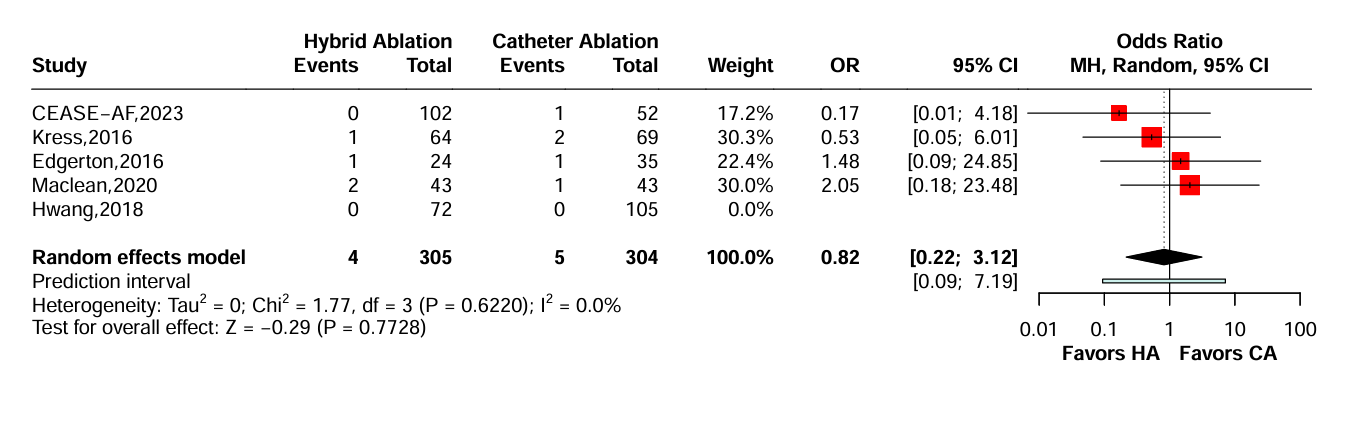


Supplemental Figure 10B.Forest Plot for Cardioversion


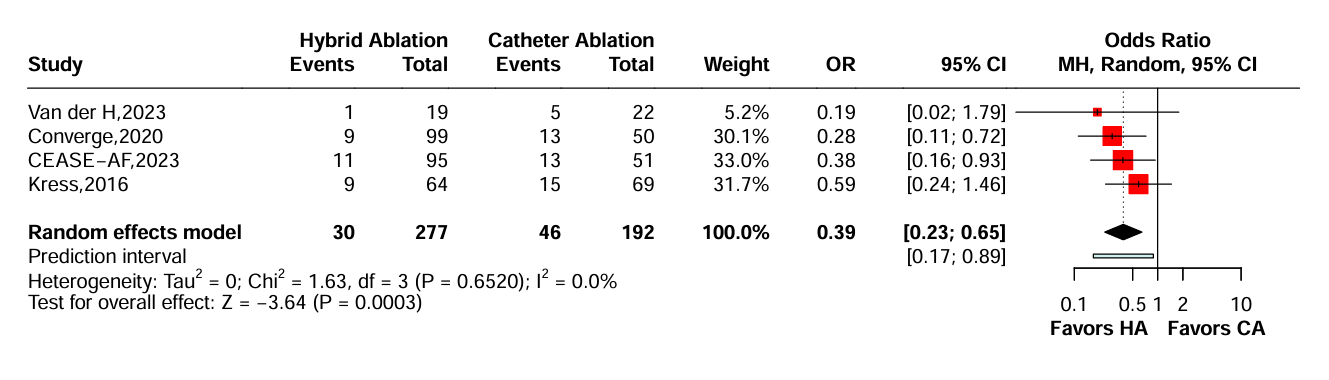


Supplemental Figure 10C.Forest Plot for Death


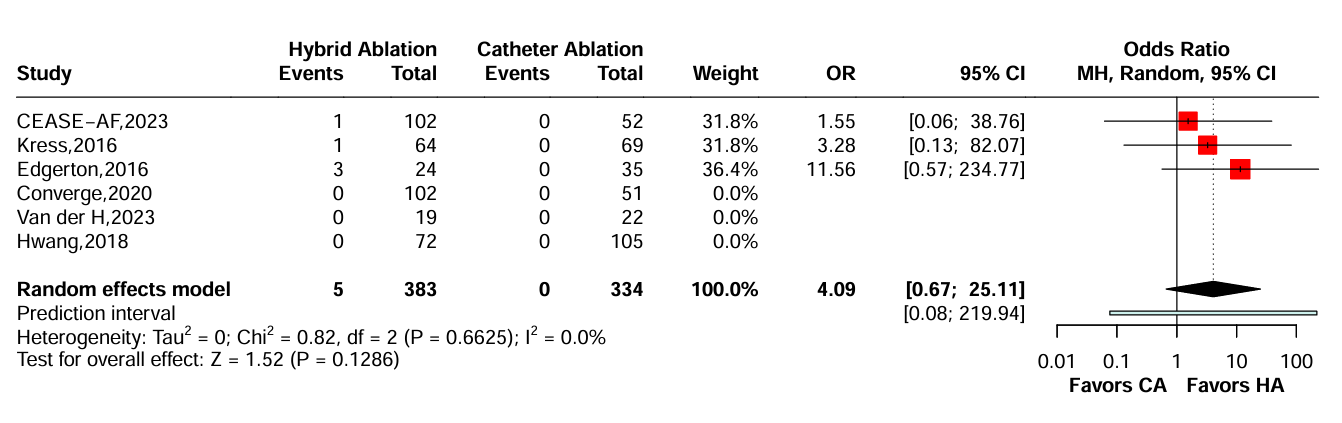


Supplemental Figure 10D.Forest Plot for Major Complications


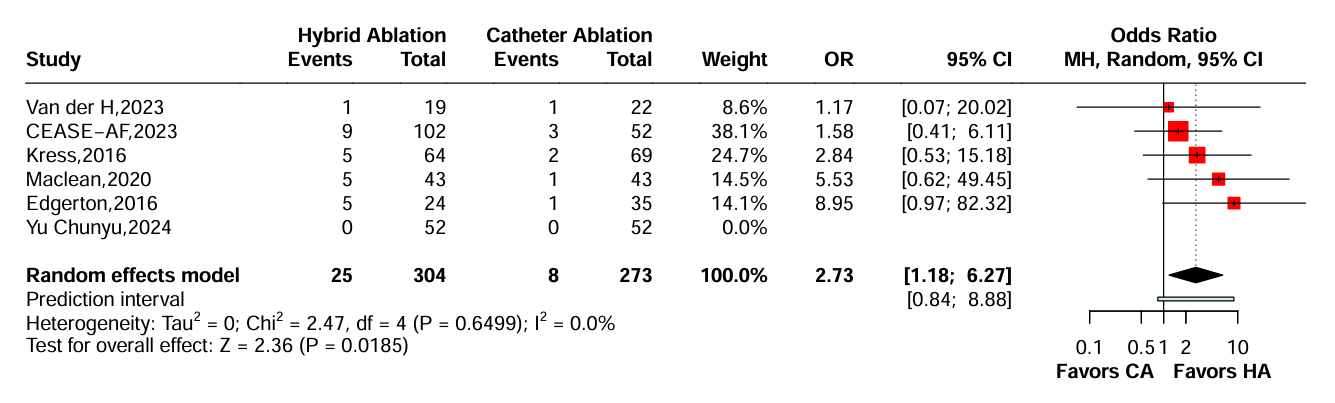


Supplemental Figure 10E. Forest Plot for Phrenic nerve paralysis


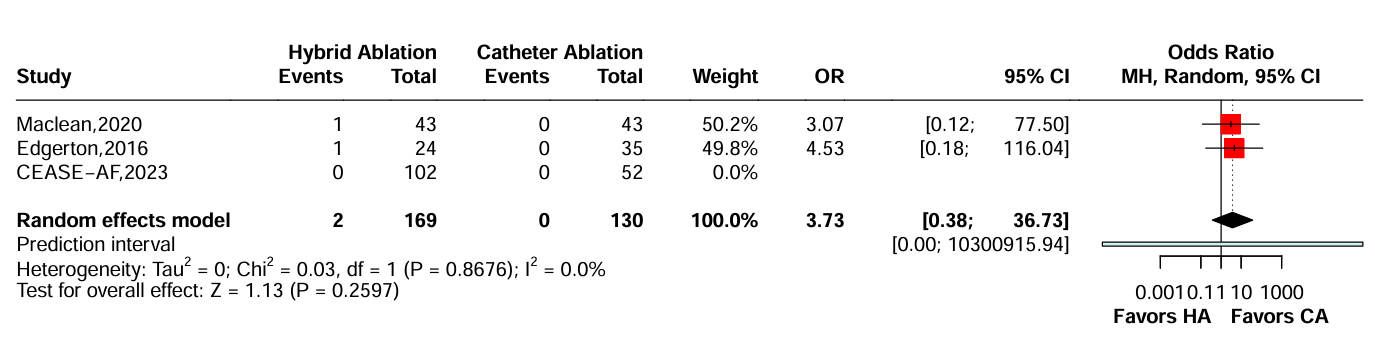


Supplemental Figure 10F.Forest Plot for Stroke


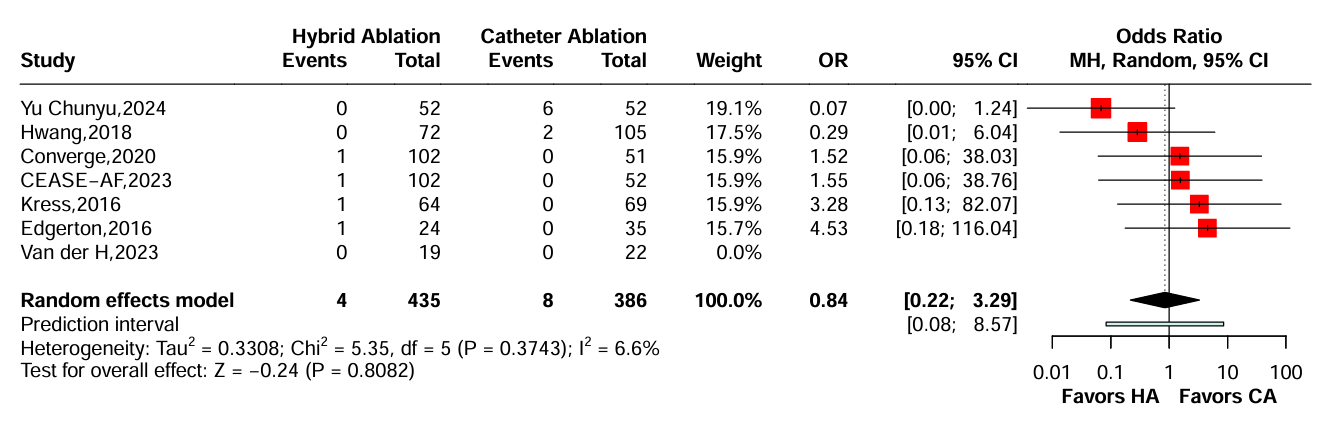


Supplemental Figure 11A. Leave-One-Out Sensitivity Analysis for Cardiac Tamponade


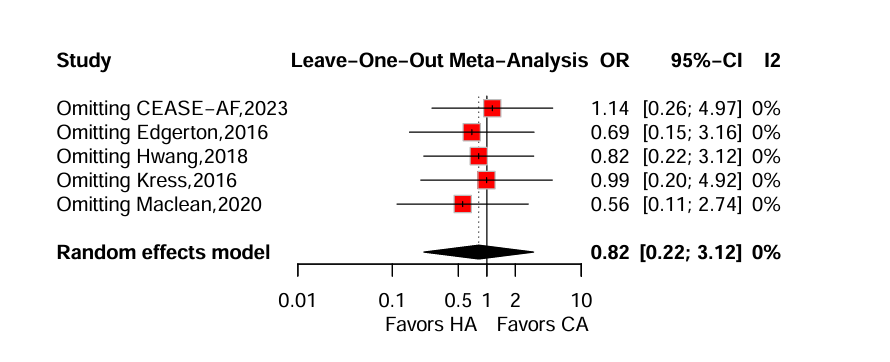


Supplemental Figure 11B. Leave-One-Out Sensitivity Analysis for Cardioversion


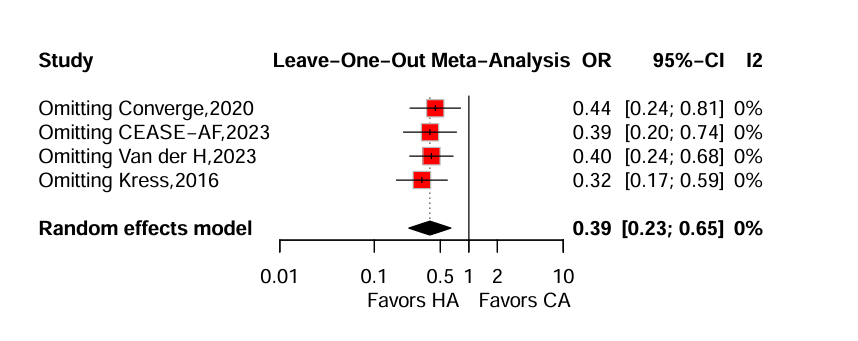


Supplemental Figure 11C. Leave-One-Out Sensitivity Analysis for Death


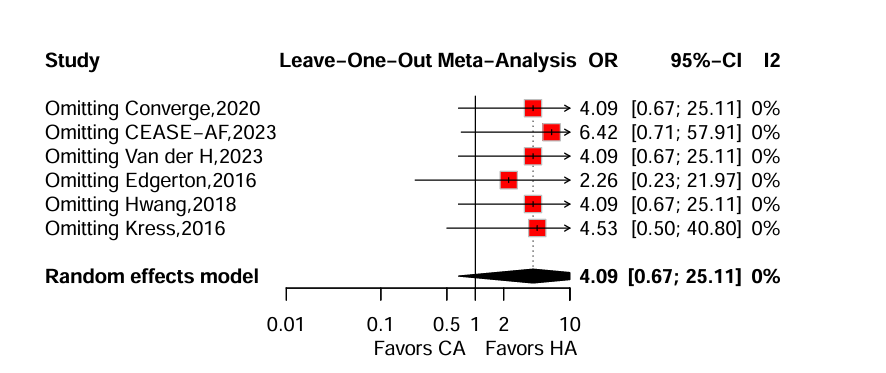


Supplemental Figure 11D. Leave-One-Out Sensitivity Analysis for Major Complications


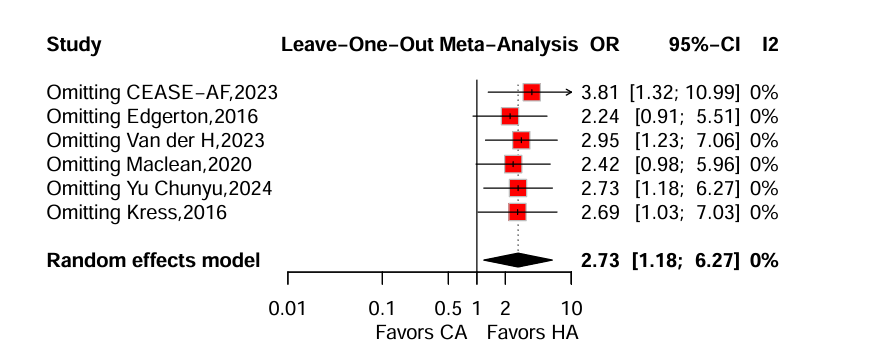


Supplemental Figure 11E. Leave-One-Out Sensitivity Analysis for Phrenic nerve paralysis


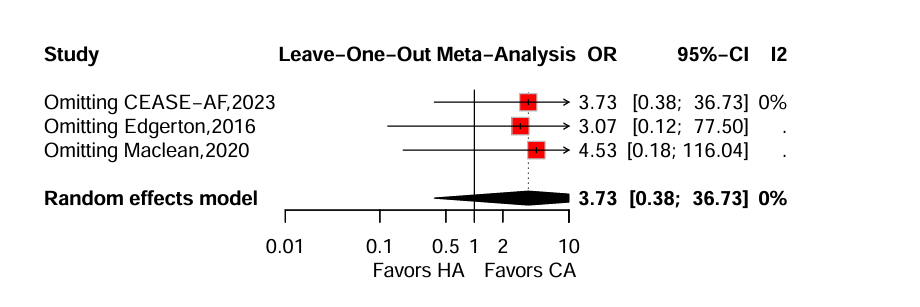


Supplemental Figure 11F. Leave-One-Out Sensitivity Analysis for Stroke


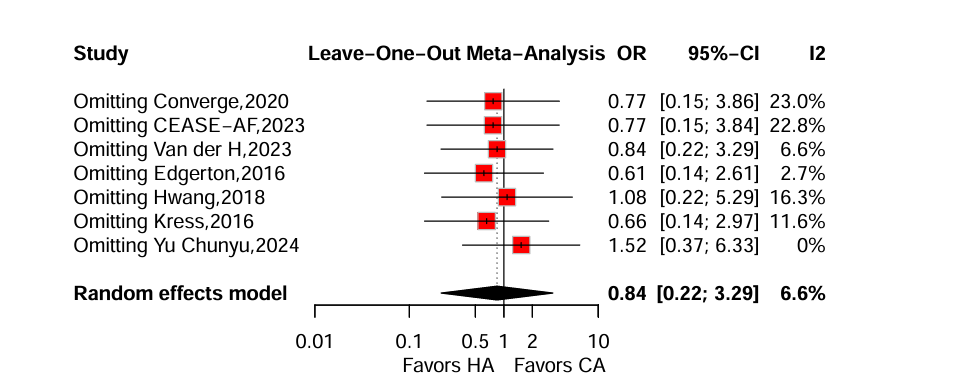


Supplemental Figure 12A. Baujat Plot for Cardiac Tamponade


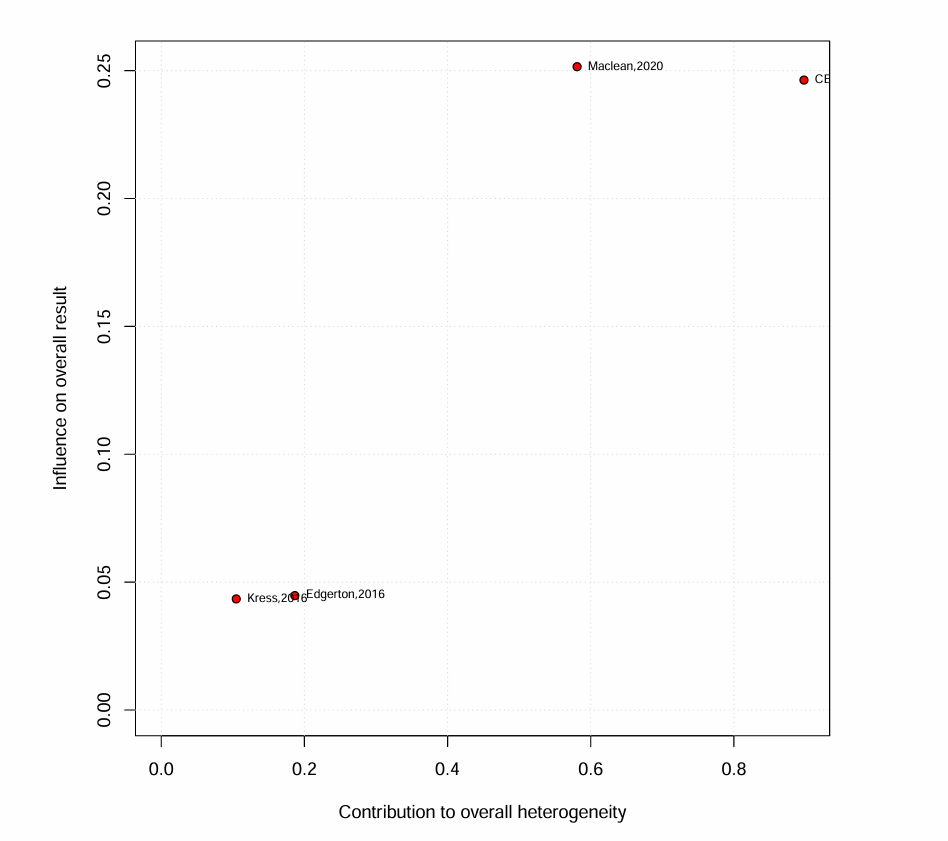


Supplemental Figure 12B. Baujat Plot for Cardioversion


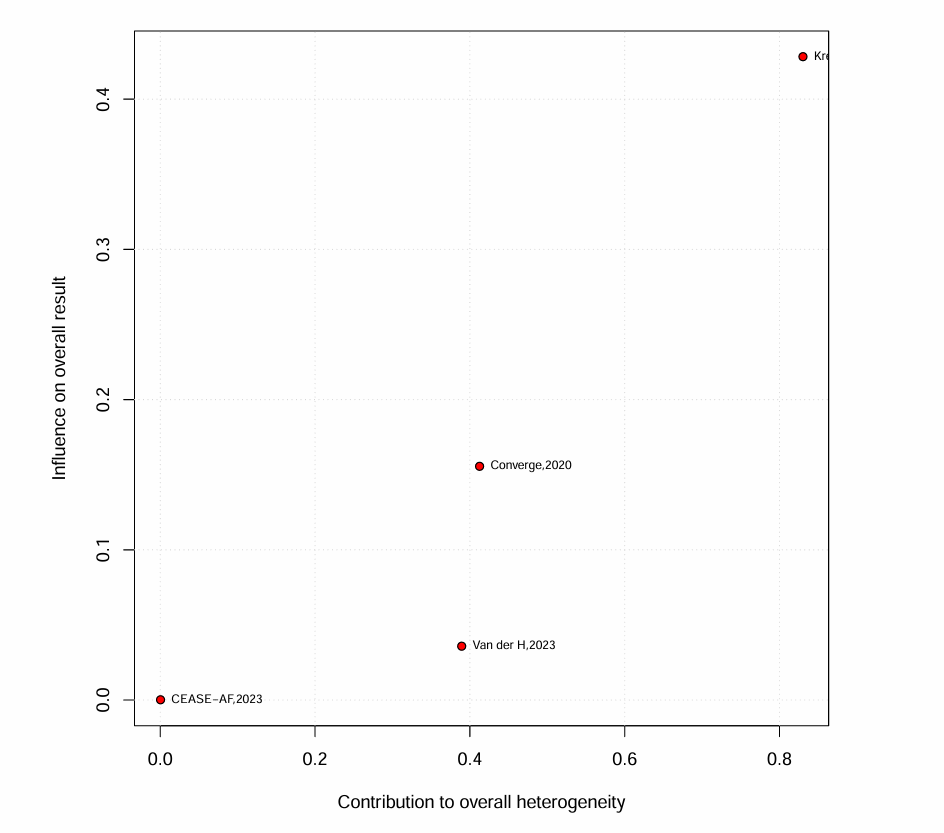


Supplemental Figure 12C. Baujat Plot for Death


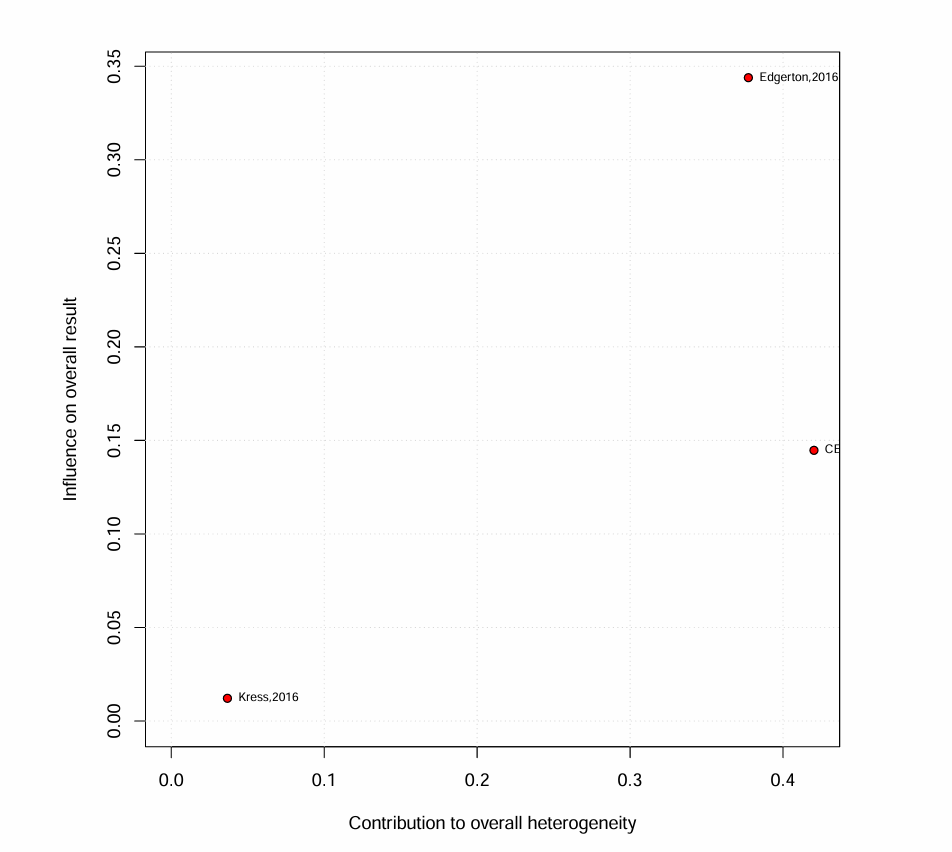


Supplemental Figure 12D. Baujat Plot for Major Complications


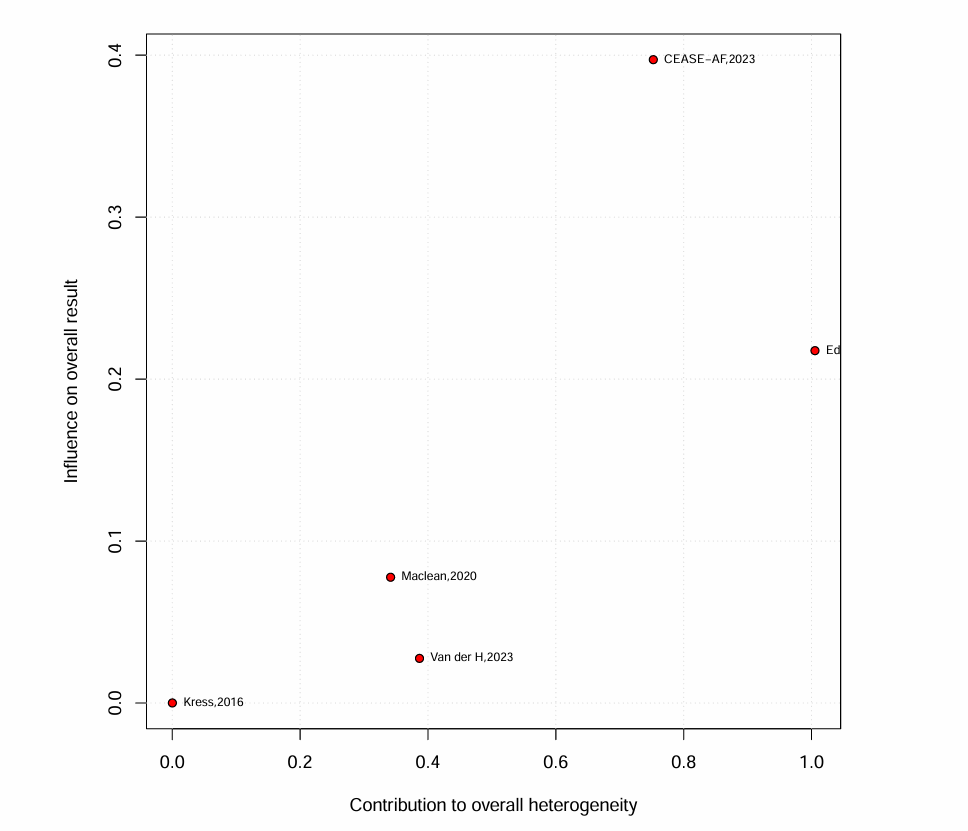


Supplemental Figure 12E. Baujat Plot for Phrenic nerve paralysis


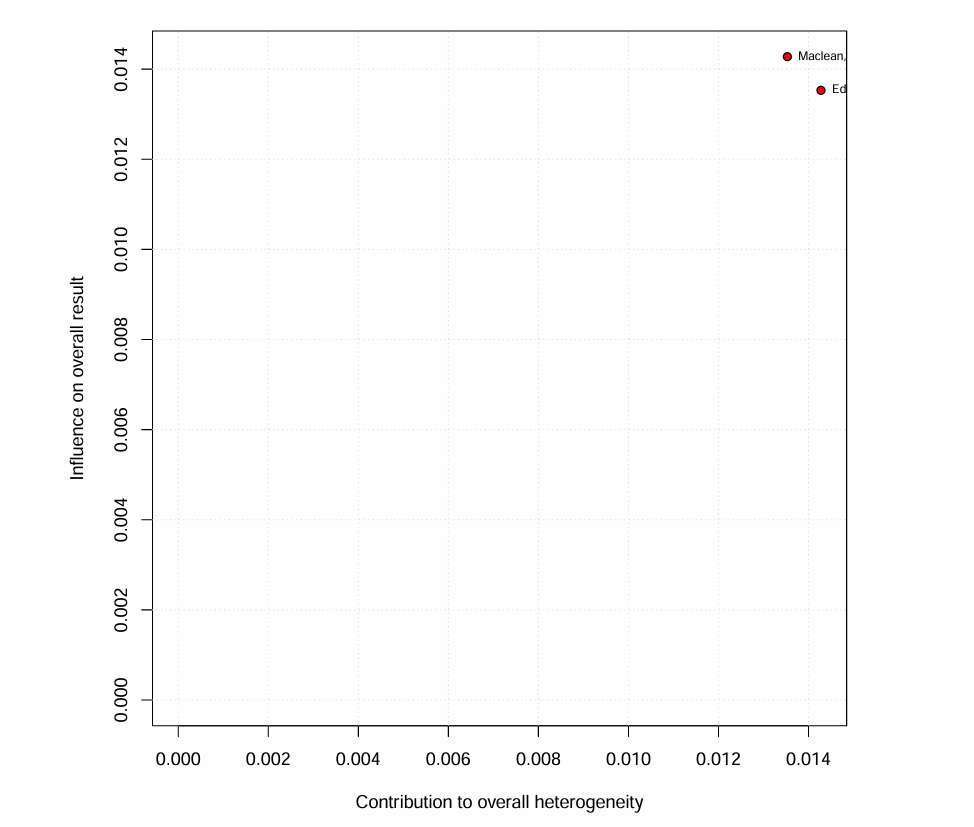


Supplemental Figure 12F.Baujat Plot for Stroke


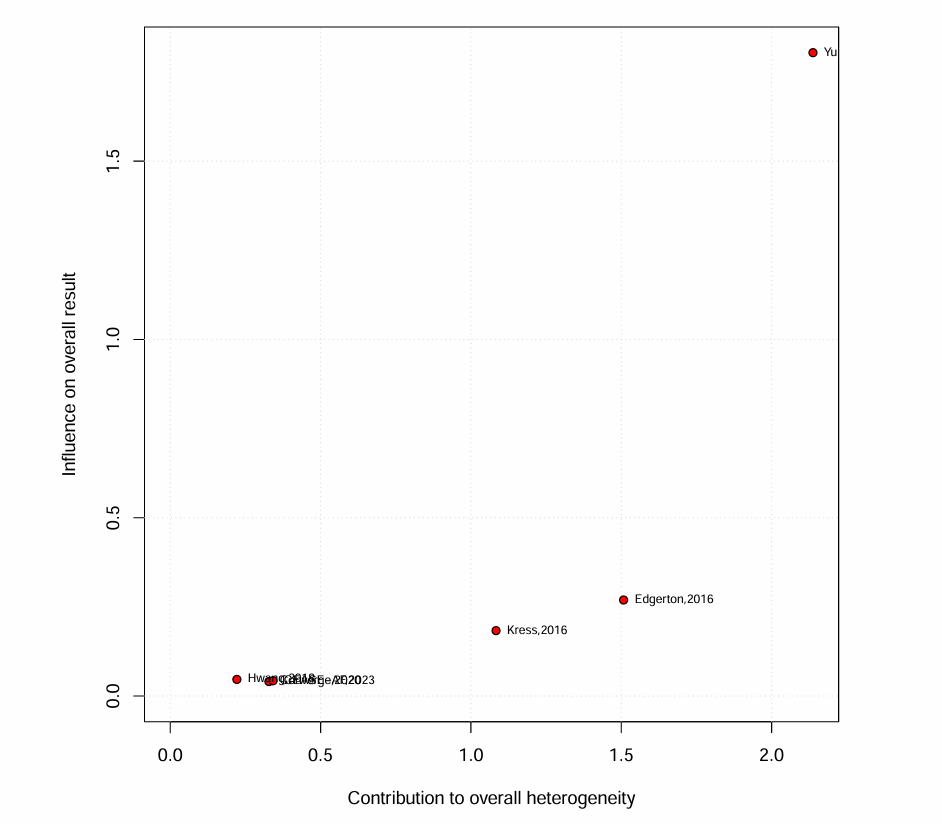


Supplemental Figure 13A. Funnel Plot for Cardiac Tamponade


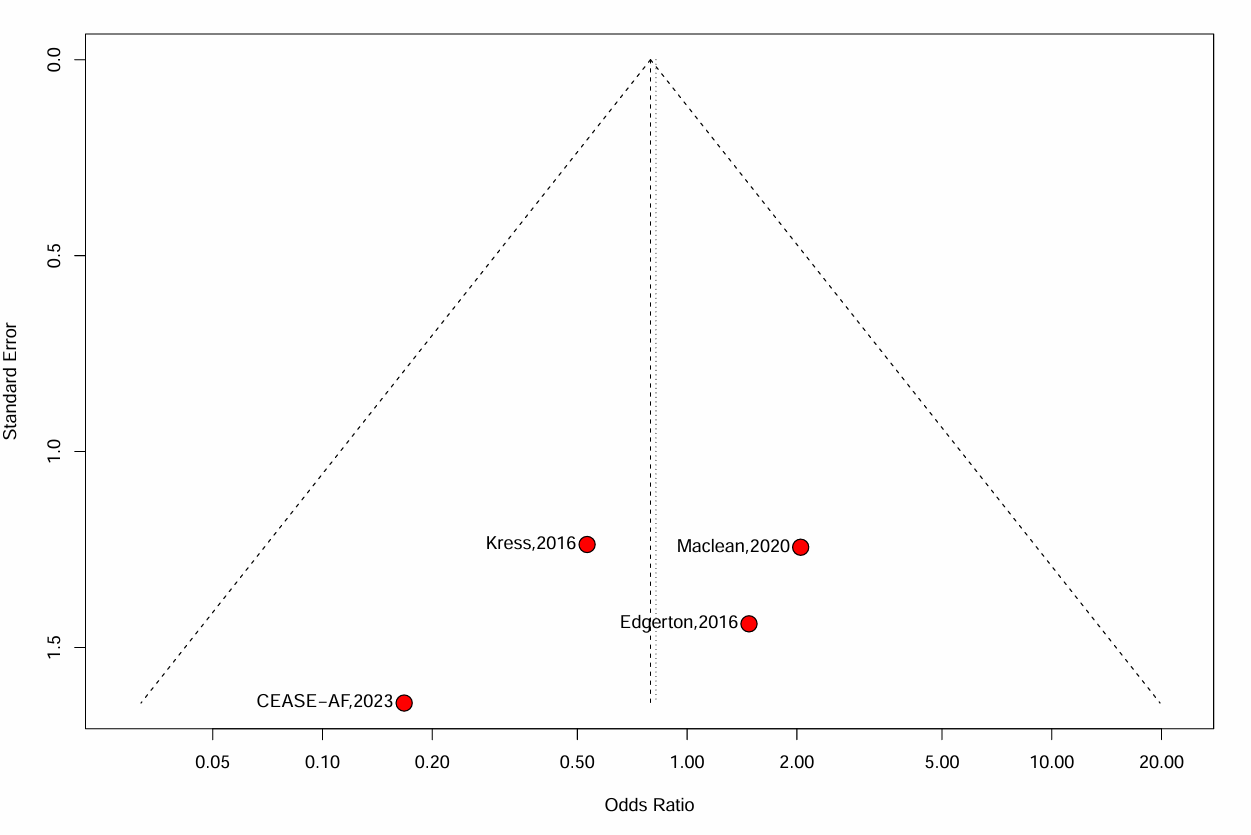


Supplemental Figure 13B. Funnel Plot for Cardioversion


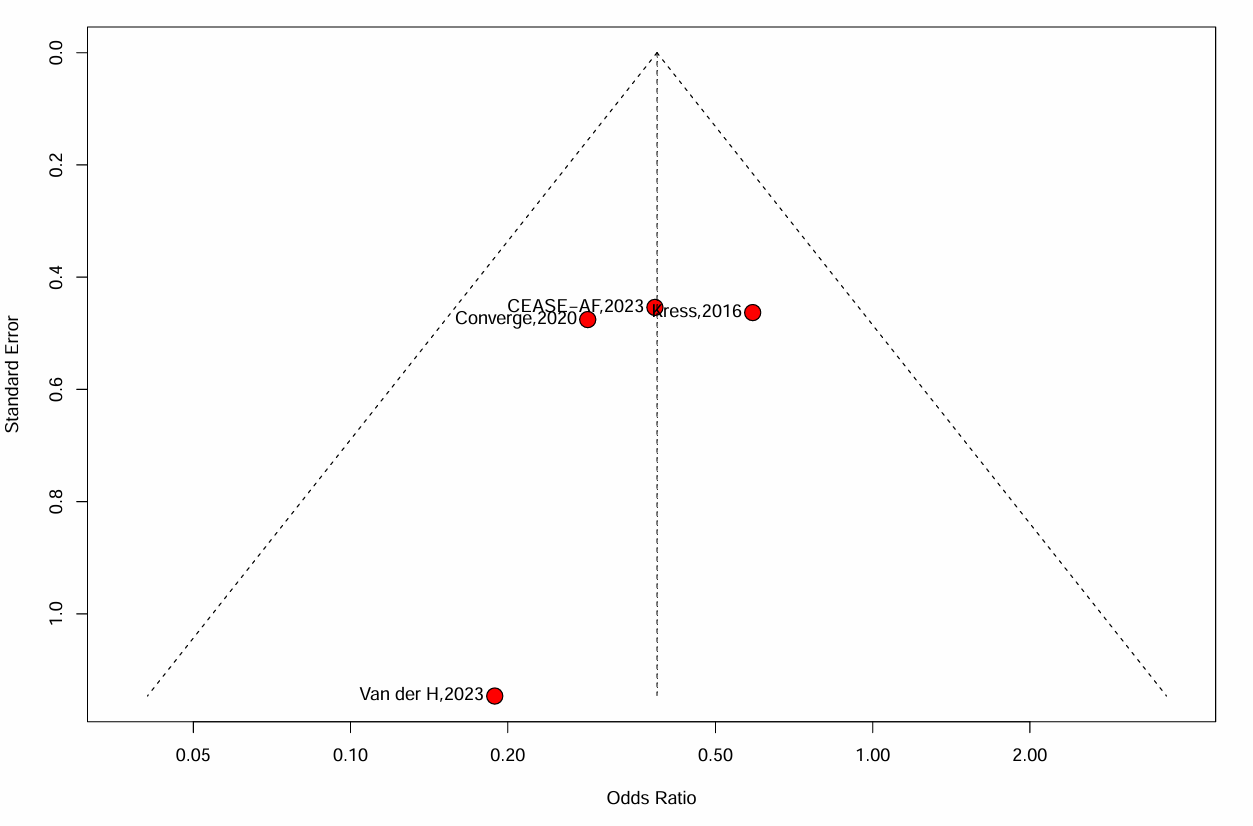


Supplemental Figure 13C. Funnel Plot for Death


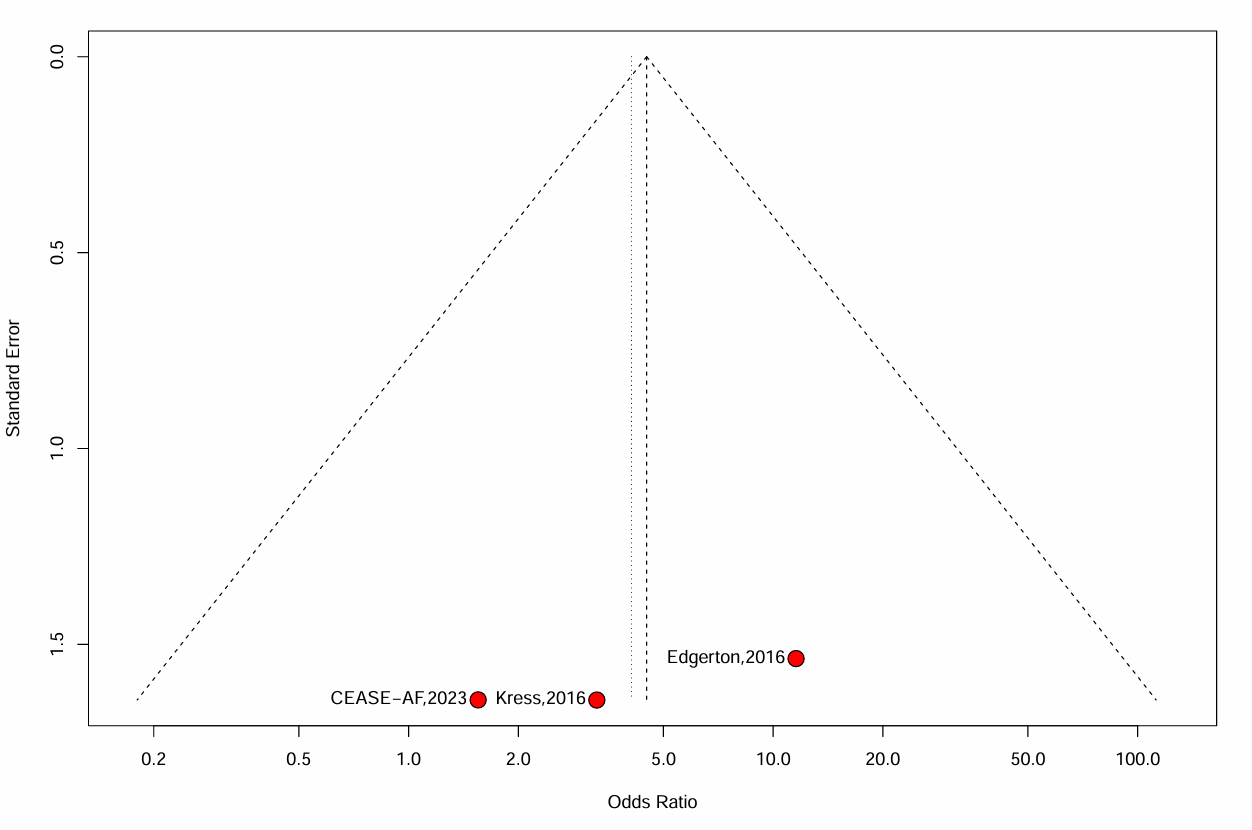


Supplemental Figure 13D. Funnel Plot for Major Complications


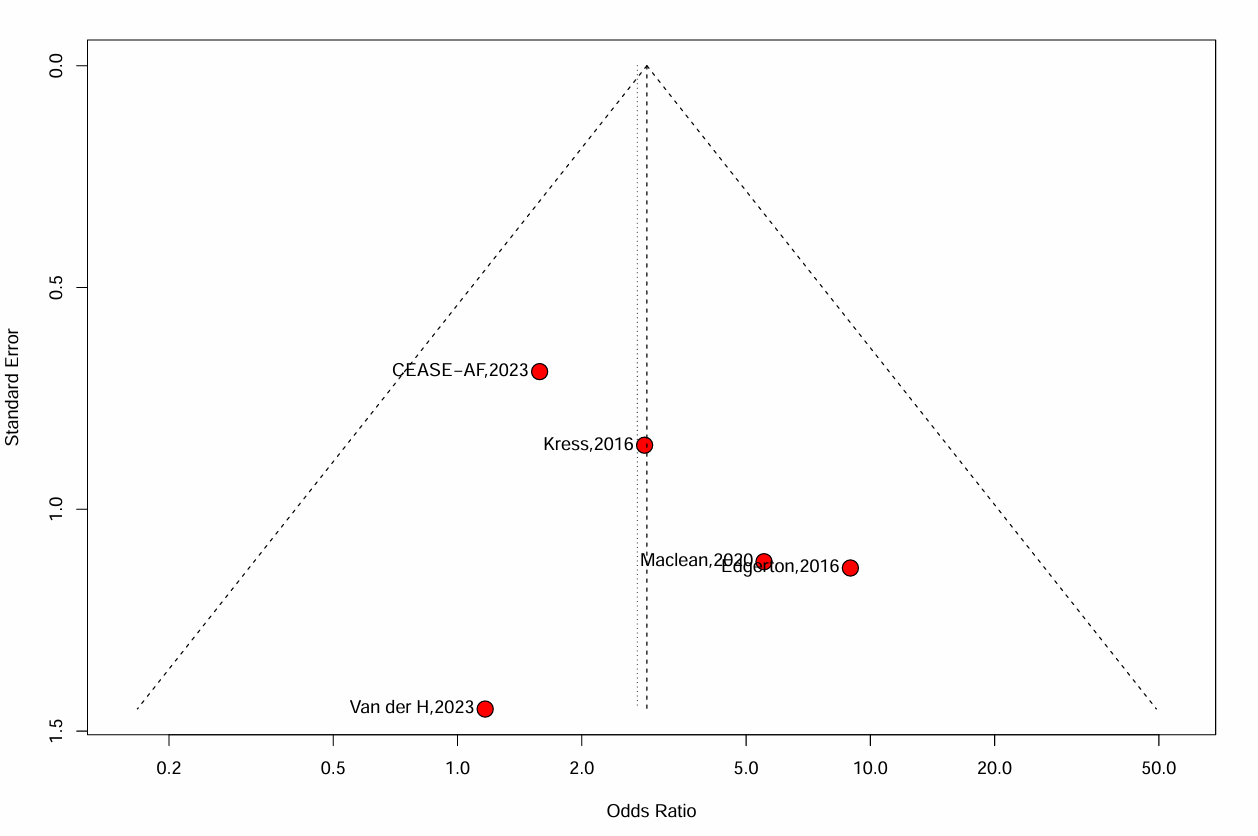


Supplemental Figure 13E. Funnel Plot for Phrenic nerve paralysis


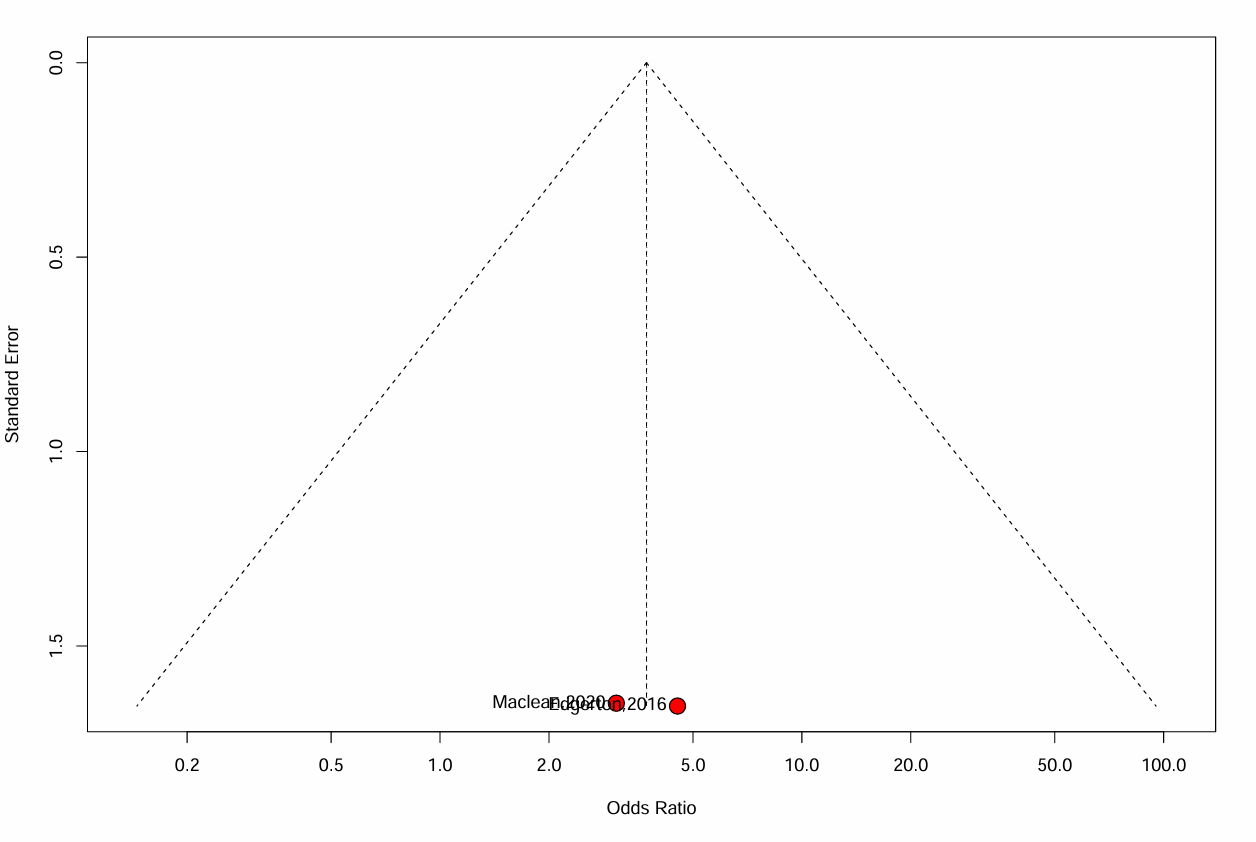


Supplemental Figure 13F. Funnel Plot for Stroke


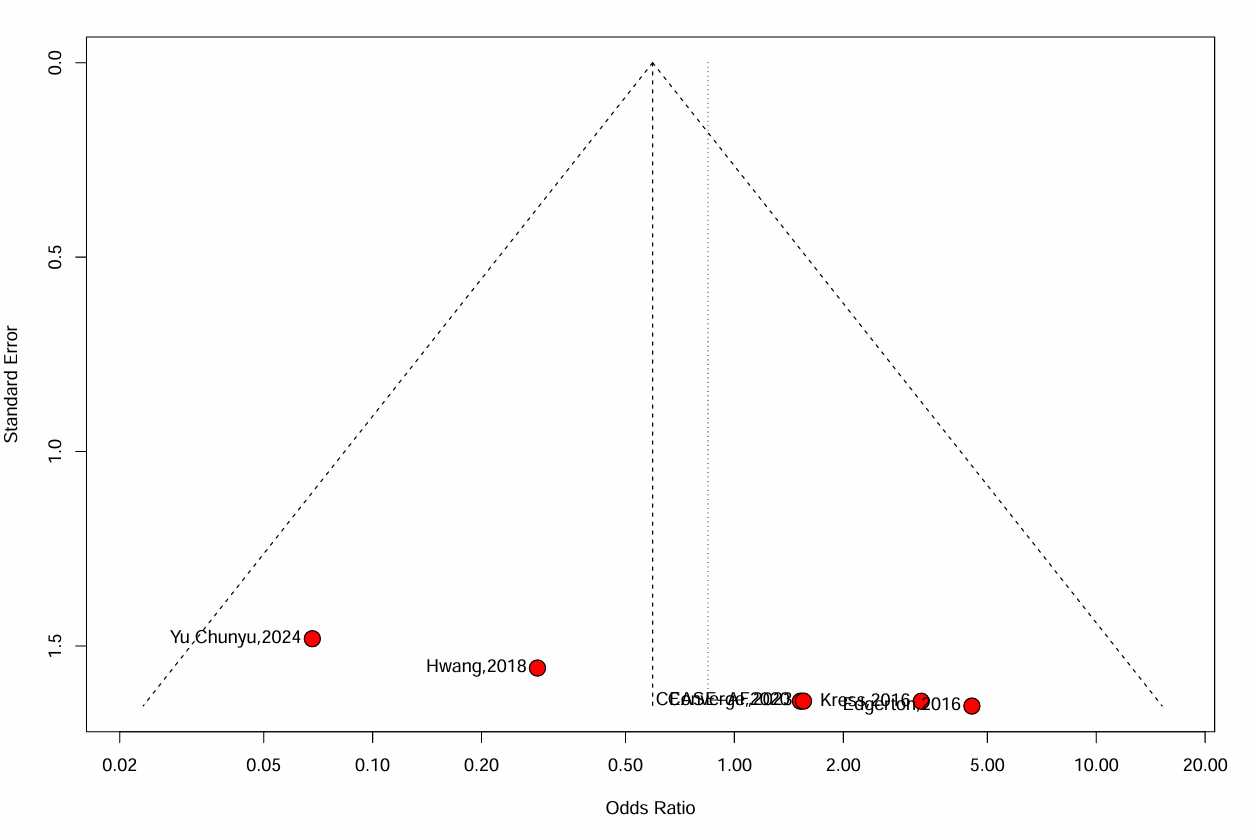


Supplemental Figure 14A.Forest Plot for Fluoroscopy time, minutes


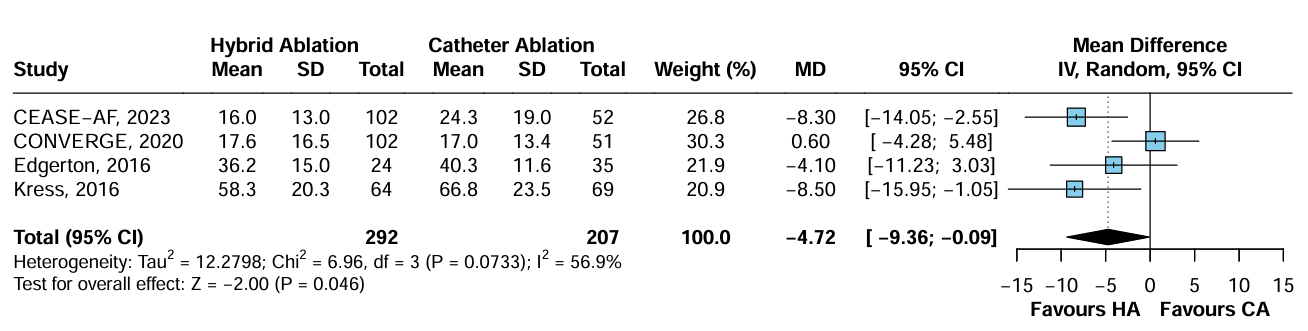


Supplemental Figure 14B.Forest Plot for Length of stay, days


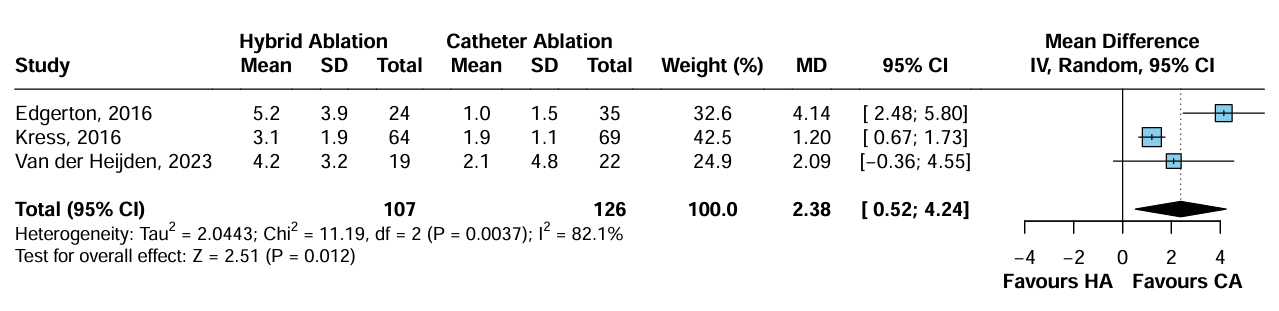


Supplemental Figure 14C.Forest Plot for Procedure time for the endocardial ablation,minutes


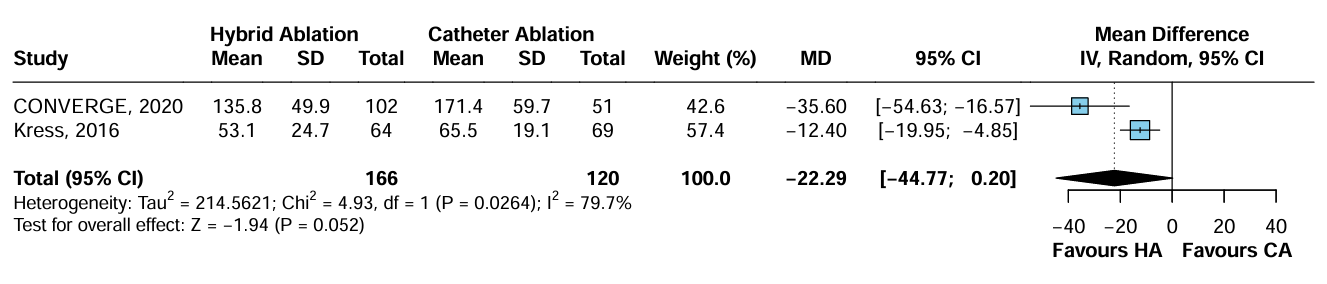


Supplemental Figure 14D.Forest Plot for Total procedure duration (minutes)


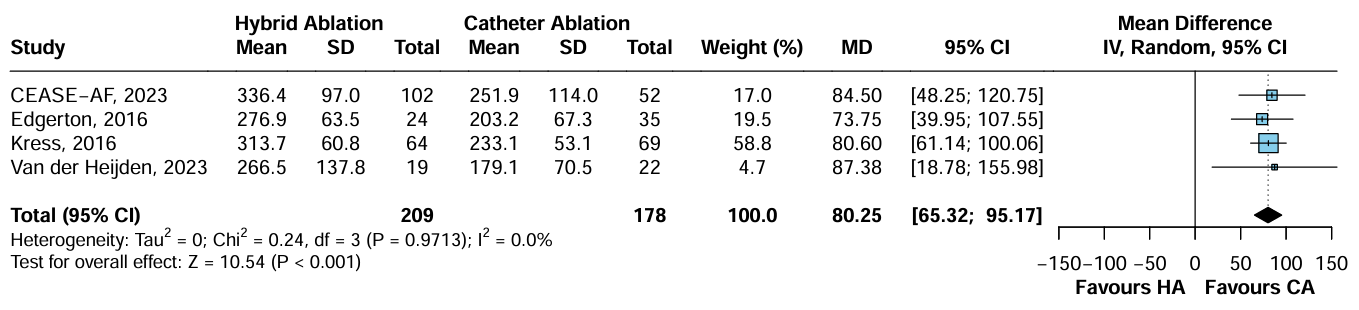


Supplemental Figure 15A. Leave-One-Out Sensitivity Analysis for Fluoroscopy time, minutes


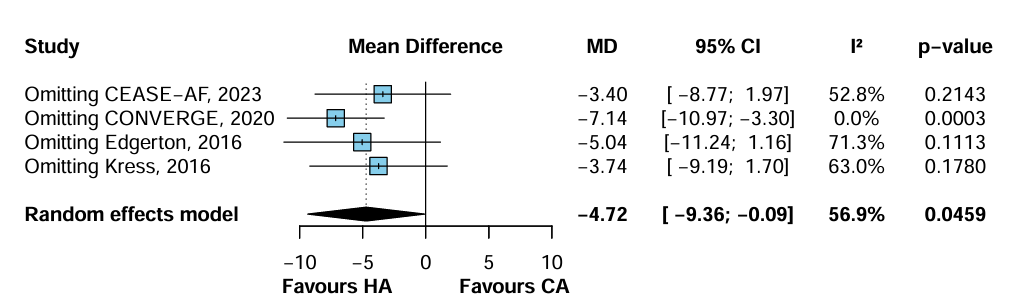


Supplemental Figure 15B. Leave-One-Out Sensitivity Analysis for Length of stay, days


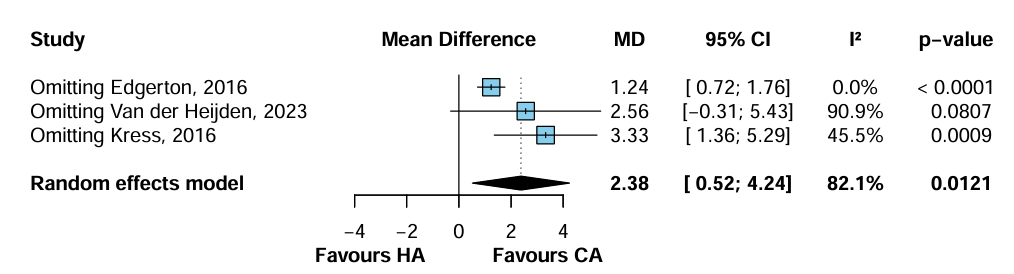


Supplemental Figure 15C. Leave-One-Out Sensitivity Analysis for Total procedure duration (minutes)


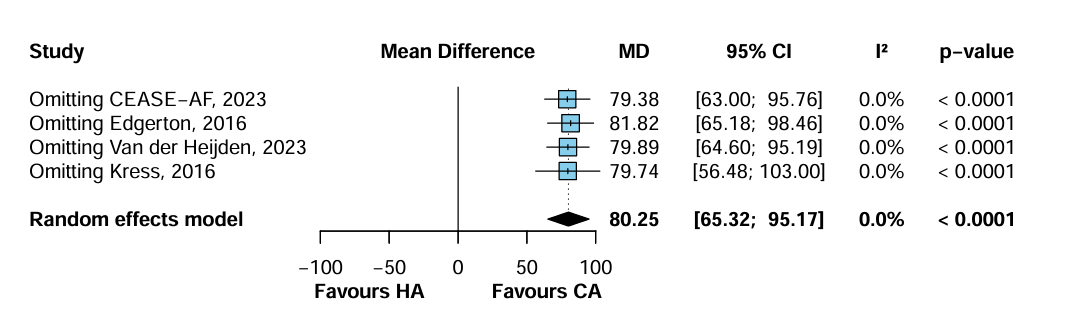


Supplemental Figure 16A. Subgroup Analysis of type of study for Fluoroscopy time,minutes


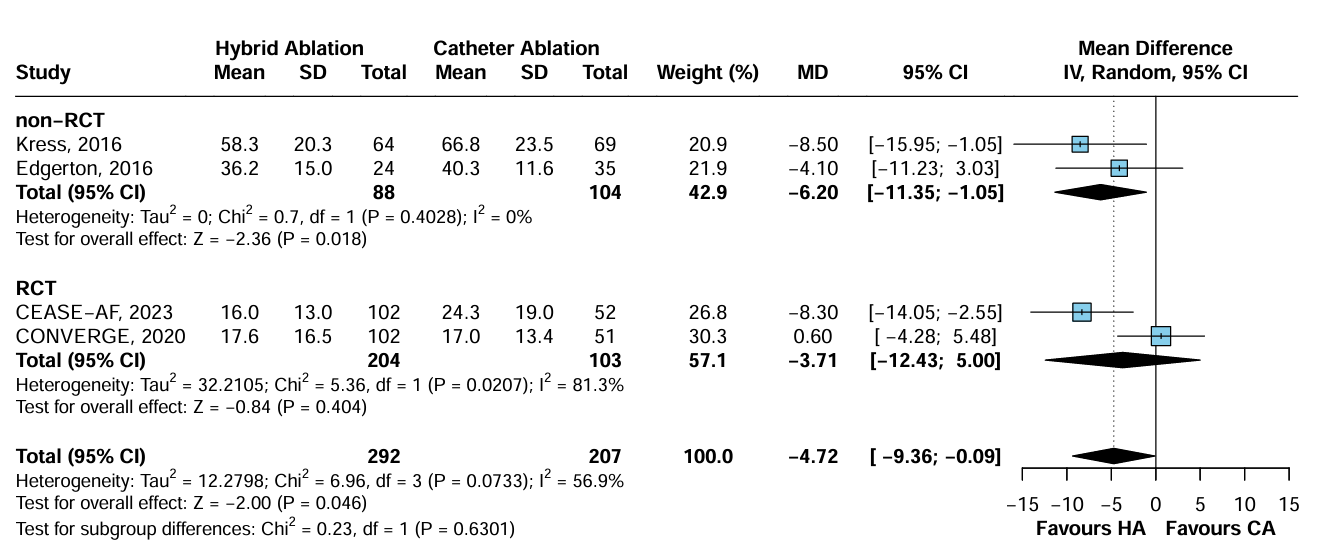


Supplemental Figure 16B. Subgroup Analysis of type of study for Length of stay, days
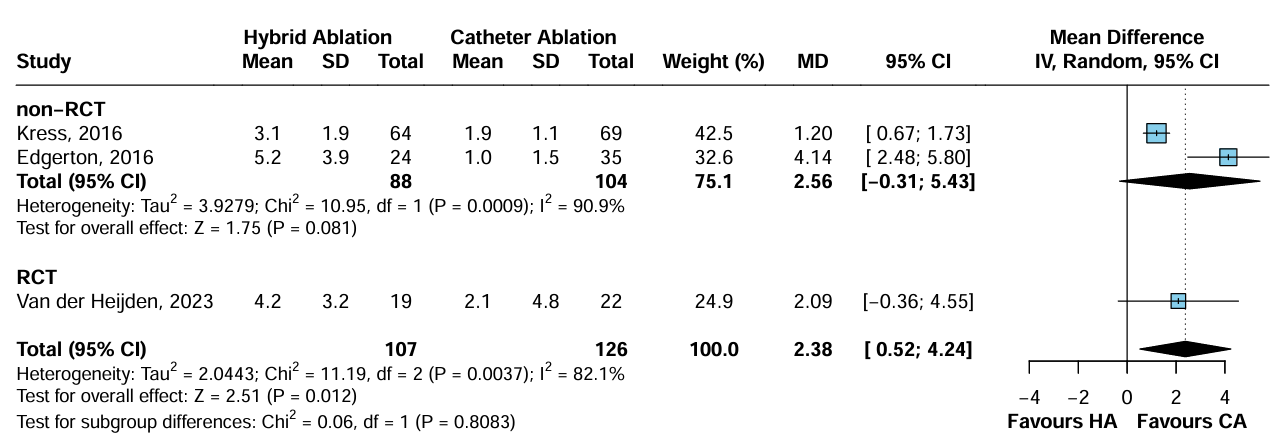


Supplemental Figure 16C. Subgroup Analysis of the type of study for Total procedure duration (minutes)


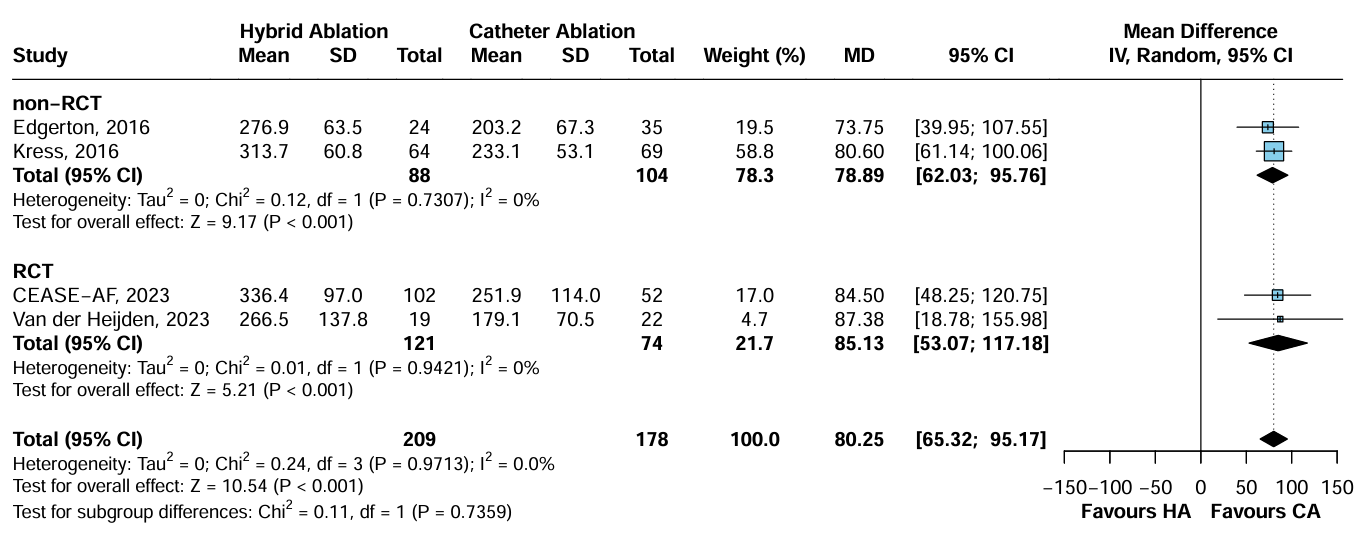


Supplemental Figure 17A. Baujat Plot for Fluoroscopy time, minutes


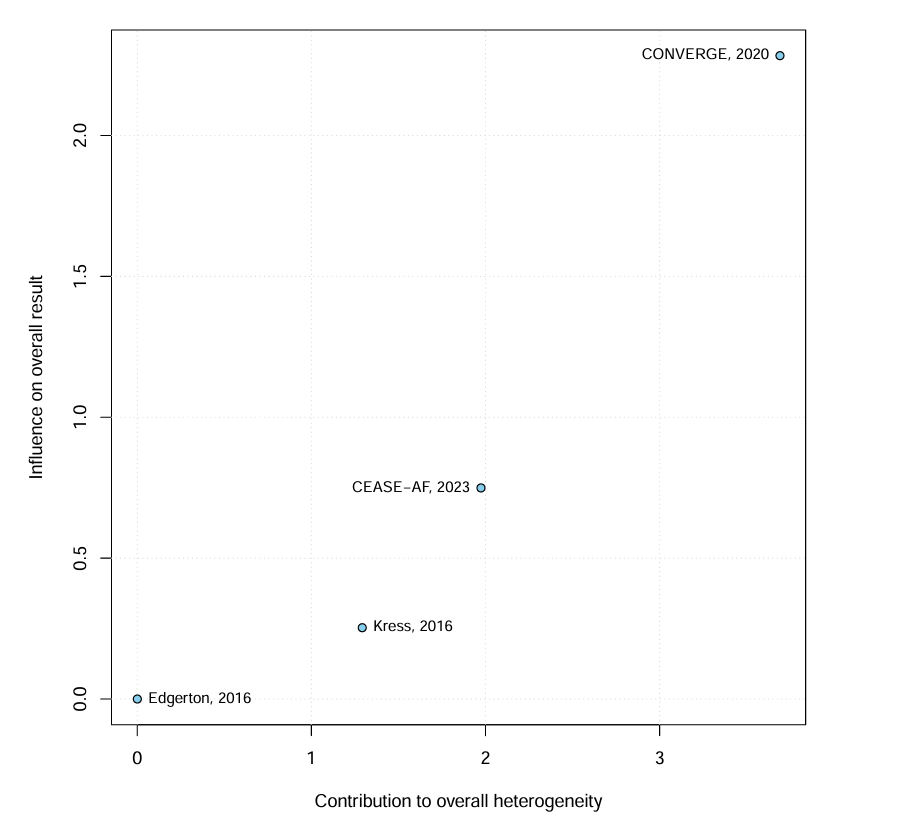


Supplemental Figure 17B. Baujat Plot for Length of stay, days


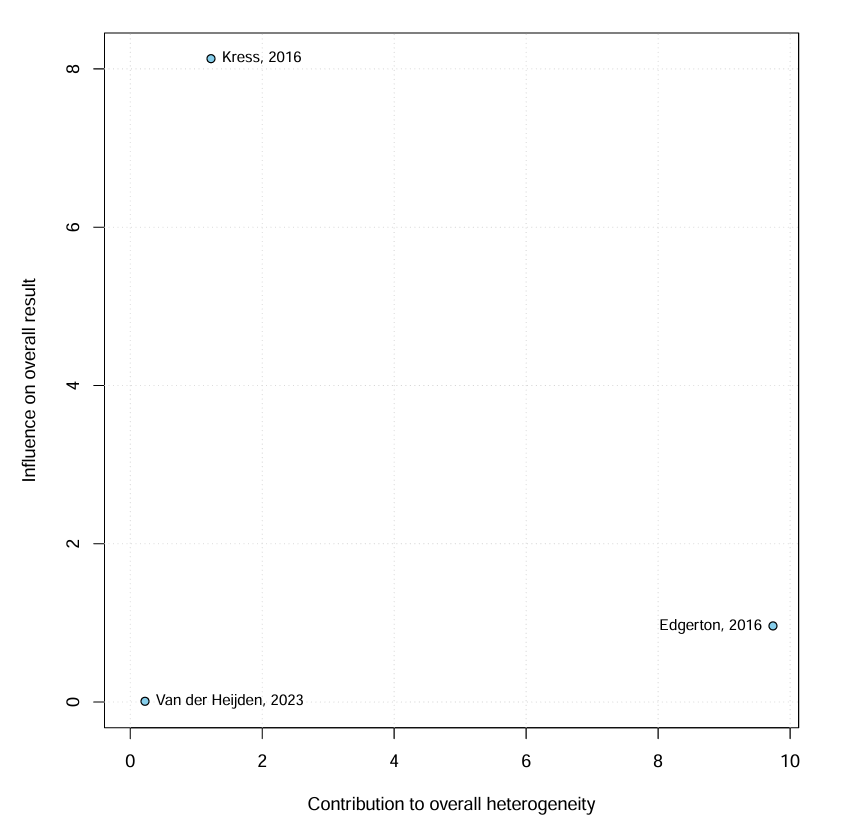


Supplemental Figure 17C. Baujat Plot for Total procedure duration (minutes)


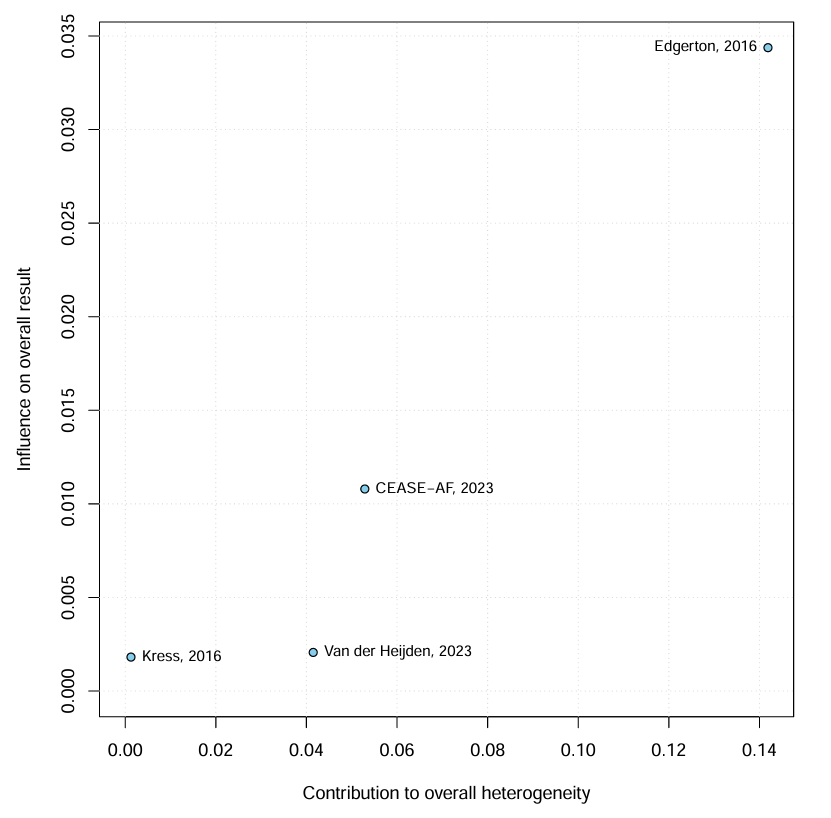


## Supplemental Results. Risk of Bias Assessment of Included Studies

Supplemental Figure 18A. Traffic Light Plot of ROBINS-I Assessment


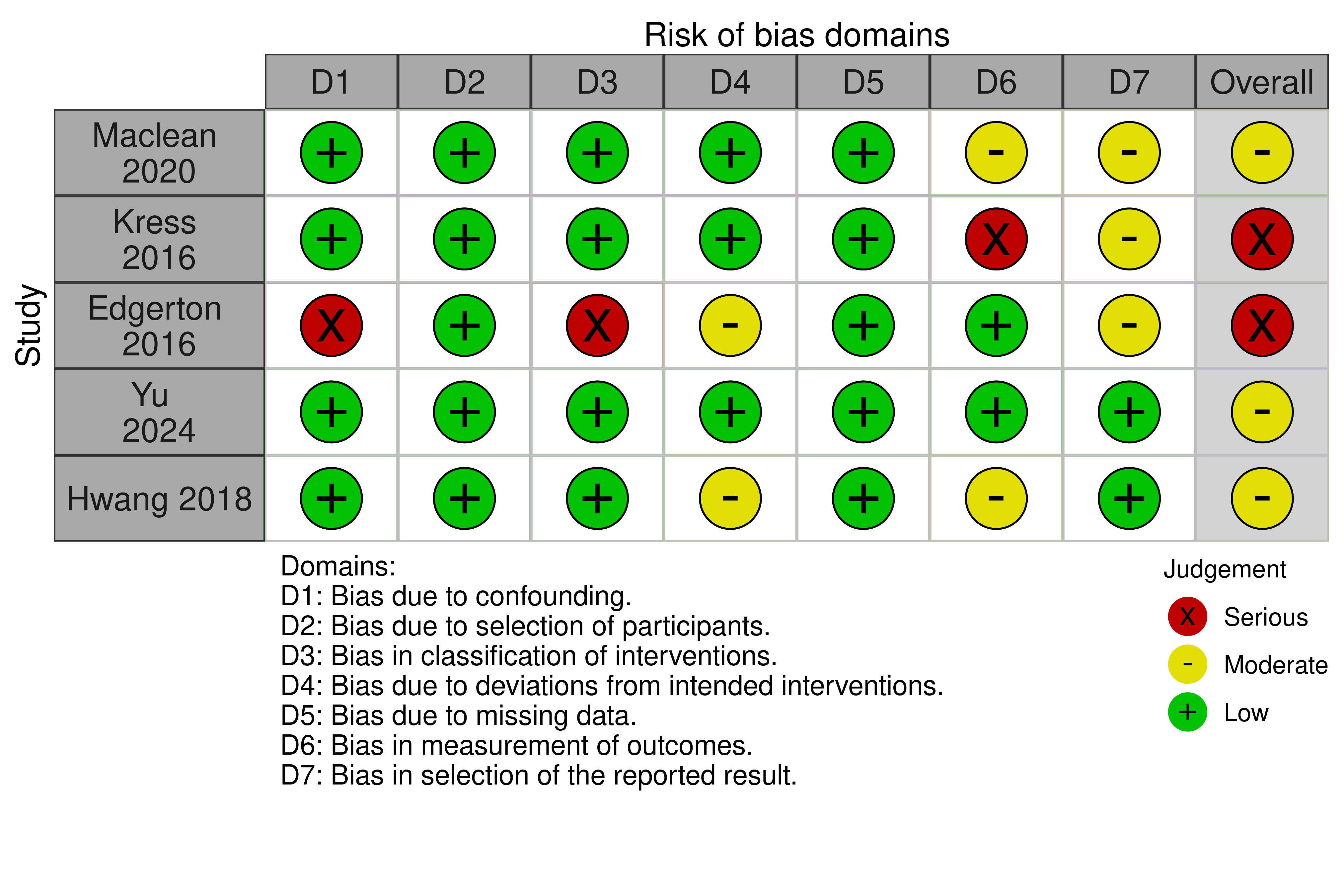


Supplemental Figure 18B. Summary Bar Plot of ROBINS-I Assessment


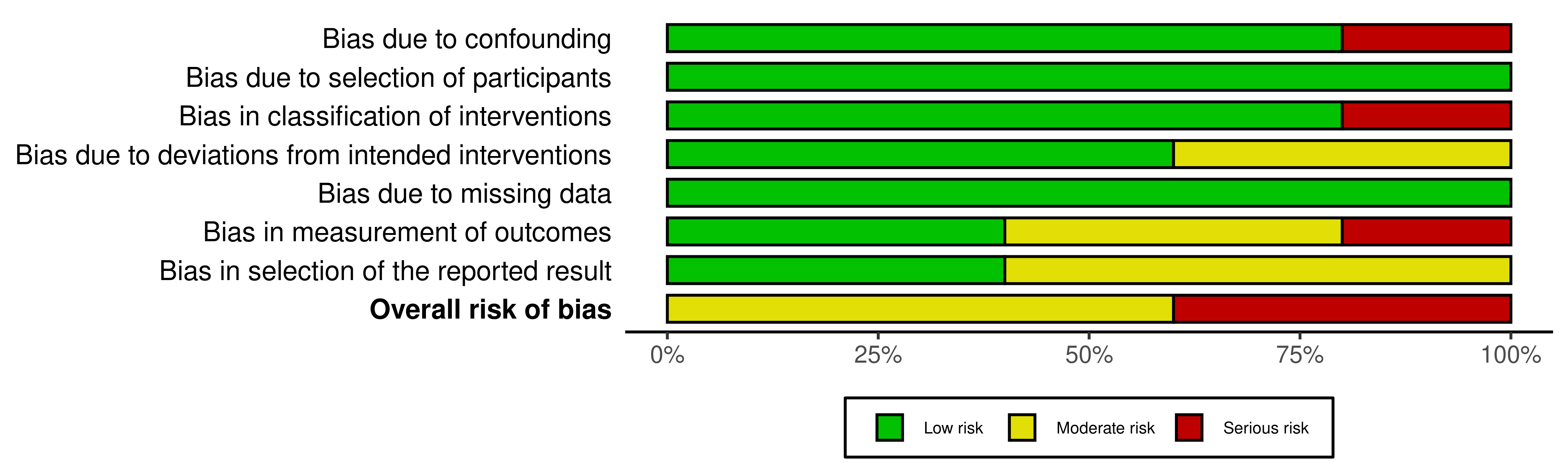


Supplemental Figure 19C. Traffic Light Plot of RoB2 Assessment


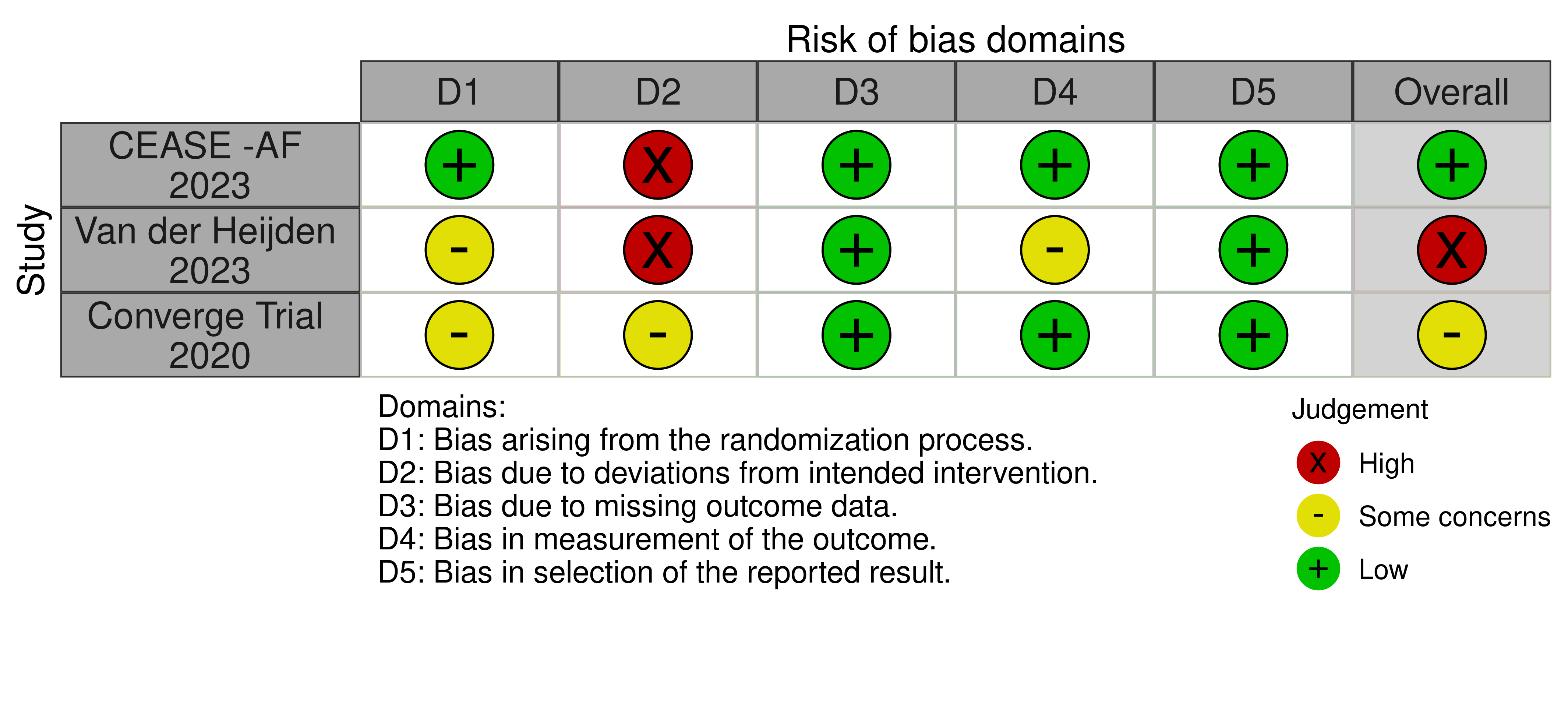


Supplemental Figure 19D. Summary Bar Plot of ROBINS-2 Assessment


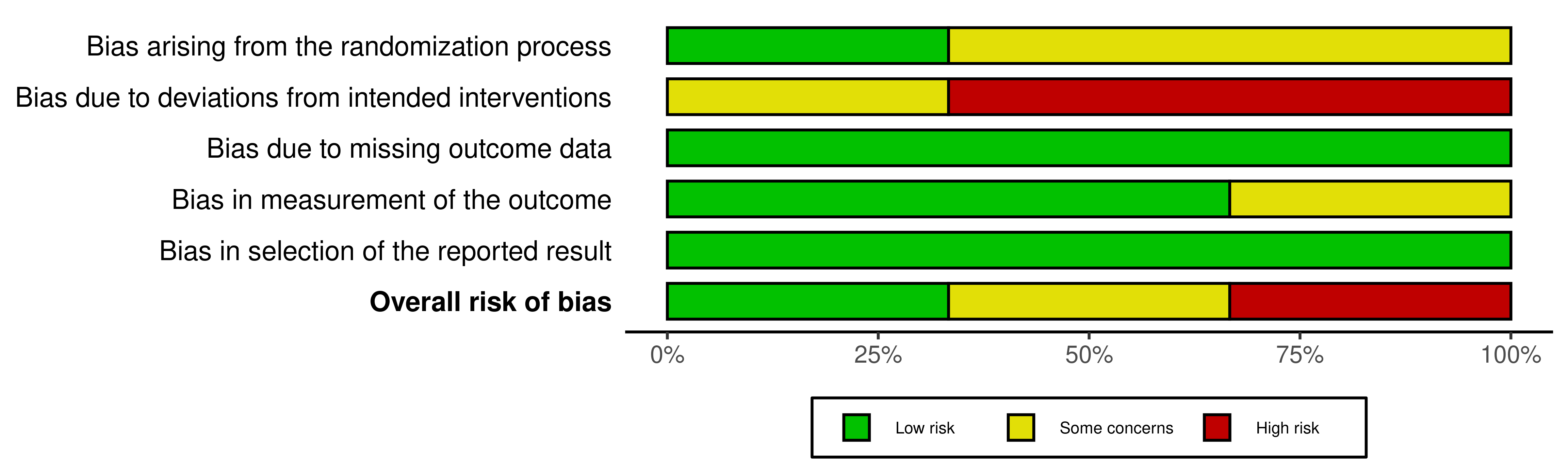

Supplement: Supplementary file 1 — Figure S1: Flow chart of selected studies. Table S1: Main Inclusion and Exclusion Criteria of Included Studies. Table S2: Definitions of Hybrid Ablation and Endocardial Ablation of Included Studies. Methods S1: PRISMA 2020 Checklist. Methods S2: PRISMA 2020 Checklist for Abstracts. Methods S3: Details of Search Strategies. Figure S1A: Leave‐One‐Out Sensitivity Analysis for Freedom from Atrial Fibrillation. Figure S1B: Leave‐One‐Out Sensitivity Analysis for Freedom from Anti‐Arrhythmic Drug (AAD). Figure S1C: Leave‐One‐Out Sensitivity Analysis for Freedom from Arrhythmia (Regardless of AADs). Figure S1D: Leave‐One‐Out Sensitivity Analysis for Repeat Ablation. Figure S1E: Leave‐One‐Out Sensitivity Analysis for Arrhythmia Recurrence. Figure S2A: Baujat Plot for Freedom from Atrial Fibrillation. Figure S2B: Baujat Plot for Freedom from Anti‐Arrhythmic Drug (AAD). Figure S2C: Baujat Plot for Freedom from Arrhythmia (Regardless of AADs). Figure S2D: Baujat Plot for Arrhythmia Recurrence. Figure S2E: Baujat Plot for Repeat Ablation. Figure S3A: Funnel Plot for Freedom from Atrial Fibrillation. Figure S3B: Funnel Plot for Freedom from Anti‐Arrhythmic Drug (AAD). Figure S3C: Funnel Plot for Freedom from Arrhythmia (Regardless of AADs). Figure S3D: Funnel Plot for Arrhythmia Recurrence. Figure S3E: Funnel Plot for Repeat Ablation. Figure S4A: Subgroup Analysis of Type of energy for Freedom from Atrial Fibrillation. Figure S4B: Subgroup Analysis of Type of Energy for Freedom from Anti‐Arrhythmic Drug (AAD). Figure S4C: Subgroup Analysis of Type of energy for Freedom from Arrhythmia (Regardless of AADs). Figure S4D: Subgroup Analysis of the Type of Energy for Arrhythmia Recurrence. Figure S4E: Subgroup Analysis of Type of Energy for Repeat Ablation. Figure S5A: Subgroup Analysis of the Type of Study for Freedom from Atrial Fibrillation. Figure S5B: Subgroup Analysis of the Type of Study for Freedom from Anti‐Arrhythmic Drug (AAD). Figure S5C: Subgroup Analysis of Type of Study [file JOA3-42-e70416-s001.zip › joa370416-sup-0001-Supinfo1@Supplemental Material JA (3).docx]
